# Insights into LSD1 and quorum sensing inhibitory potential of phytoconstituents isolated from *Ardisia elliptica* Thunb aerial parts

**DOI:** 10.1039/d5ra02005k

**Published:** 2025-09-08

**Authors:** Ereny M. Abdelmalek, Lourin G. Malak, Wesam S. Qayed, Mai A.M. Ahmed

**Affiliations:** a Department of Pharmacognosy, Faculty of Pharmacy, Assiut University Assiut 71526 Egypt mai.abdelhadi@pharm.aun.edu.eg; b Department of Medicinal Chemistry, Faculty of Pharmacy, Assiut University Assiut 71526 Egypt

## Abstract

*Ardisia elliptica* Thunb is endogenous to Southeast Asia and traditionally used for the treatment of bacterial and viral infections. Previous studies reported various pharmacological activities, including cytotoxic activity. The aim of this work was to identify phytoconstituents of the ethanolic extract of *Ardisia elliptica* aerial parts using extensive 1D- and 2D-NMR analysis and HR-MS. Additionally, computational techniques were utilized in drug discovery to explore the Lysine-specific demethylase 1 (LSD1) and quorum sensing (QS) inhibitory activity of the identified compounds *via* molecular docking studies. Twelve structurally diverse compounds were isolated and identified, including two undescribed phenolic *C*-glycosides (2 and 5). Furthermore, our *in silico* studies on the isolates identified the LSD1 and QS inhibitory potential against *Chromobacterium violaceum* strain. The study revealed that soulieana acid (4) possess a significantly high docking score to LSD1 (*S* −12 kcal mol^−1^), while the megastigmane (8) exhibited the best binding interactions to CviR QS protein (*S* ≈ −13 kcal mol^−1^). Taken together, these findings contribute to justifying the pharmacological and traditional use of the plant, demanding further studies on their suitability as candidates for the development of anticancer and antibacterial drugs.

## Abstract 2

Isolation of two new phenolic *C*-glucosides and *in silico* LSD1 and quorum sensing inhibitory potential of phytoconstituents isolated from *Ardisia elliptica* Thunb aerial parts.

## Graphical abstract

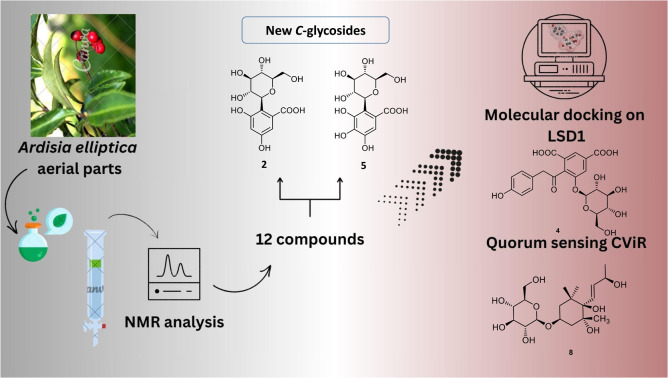

## 1. Introduction

*Ardisia* is the largest pantropical genus belonging to the family Primulaceae.^1^ Most species are distributed in Southeast Asia; a few are found in Africa, including two species categorized as endangered in Southern Madagascar.^2,3^ Phytochemical investigations of *Ardisia* species have reported the isolation of various phytoconstituents such as phenolics, triterpenoids, coumarins, quinones, and flavonoids.^4–6^ Additionally, several previous studies have reported that *Ardisia* species possess cytotoxic effects.^7–12^

*Ardisia elliptica* Thunb. (Syn. *Ardisia squamulose*), commonly known as duck's-eyes or shoe-button ardisia.^13^ It is a tropical shrub indigenous to Southeast Asia which frequently creates dense stands that result in exclusion of native plants.^14^ A comprehensive review of existing research revealed a scarcity of phytochemical investigations involving *A. elliptica* aerial parts extract. According to these studies, a mixture of triterpenoids *i.e.*, *α*-amyrin, *β*-amyrin, and bauerenol was isolated, together with two alkenylresorcinol derivatives named 5-(*Z*-heptadec-4′-enyl) resorcinol and 5-pentadecylresorcinol.^15,16^ In contrast, several biological studies on the leaf extract have been conducted, revealing promising antidiabetic effect and inhibitory action on collagenase, tyrosinase, and *α*-glucosidase enzymes.^17–19^ In addition to angio-suppressive and platelet-activating factor antagonist activities attributed to the triterpenoid and alkenyl-resorcinol content of the leaves, respectively.^20,21^ Also, the leaves are widely consumed in Southeast Asia for the treatment of bacterial^22–26^ and viral infections.^27^ Nevertheless, there are limited studies regarding the correlation of its phytoconstituents with these bioactivities.

Phytocompounds are a valuable source of drug discovery due to their remarkable chemical and functional diversities. Numerous phytocompounds have been used to date in cancer and antibacterial therapies to design and develop new drugs.^28^ Moreover, computational approaches like virtual screening, molecular dynamics, pharmacophore modeling, network biology, and machine learning (ML) have grown significantly as they are more efficient, less time-consuming, and less expensive.^29^ A major benefit of structure-based drug design is the ability of molecular docking simulation to provide the ligand-protein binding configurations for the prediction of the optimal conformation and favorable binding sites.^30^

Numerous phenolic and alcoholic substances originating from plant materials have exhibited the ability to impede Lysine-specific demethylase 1 (LSD1). In this regard, natural polyphenol compounds, such as resveratrol, curcumin, quercetin, and baicalin are reported to have the ability to inhibit LSD1 *in vitro* and/or *in vivo*.^31^ LSD1 has received substantial and expanding interest as a therapeutic target in human cancers. Recent studies have found that this demethylase is essential for both the normal growth of tissues and the preservation of tissue homeostasis.^32^ Therefore, the overexpression of LSD1 is associated with the development and progression of many diseases like cancer. Further research has proven that LSD1 inhibition can have an anticancer effect, making LSD1 a potentially effective antitumor target.^33,34^

Finding novel alternatives to established antimicrobial drugs is prompted by the rise of multidrug-resistant bacteria, particularly those with anti-virulence characteristics s quorum sensing (QS) inhibition.^35^ Recently, substantial research regarding the ability of plant extracts and phenolic compounds to inhibit QS has been published. Throughout the plant world, phenolic compounds are secondary metabolites that are extensively dispersed. Because of their antibacterial, antioxidant, and anti-inflammatory properties, they are among the most researched class of bioactive chemicals. They are also reported in the literature as QS inhibitors in *pseudomonas aeruginosa* and *Chromobacterium violaceum*. For instance, compounds like gingerol, vanillic acid, rosmarinic acid, and naringenin act as QS inhibitors through various mechanisms.^35^ Although there are a limited number of publications devoted to elucidating the mechanisms behind QS inhibition, these publications are still relatively scarce compared to those that evaluate the inhibitory effects of plants or isolated natural compounds on QS-regulated phenotypes.^36^

**Fig. 1:**
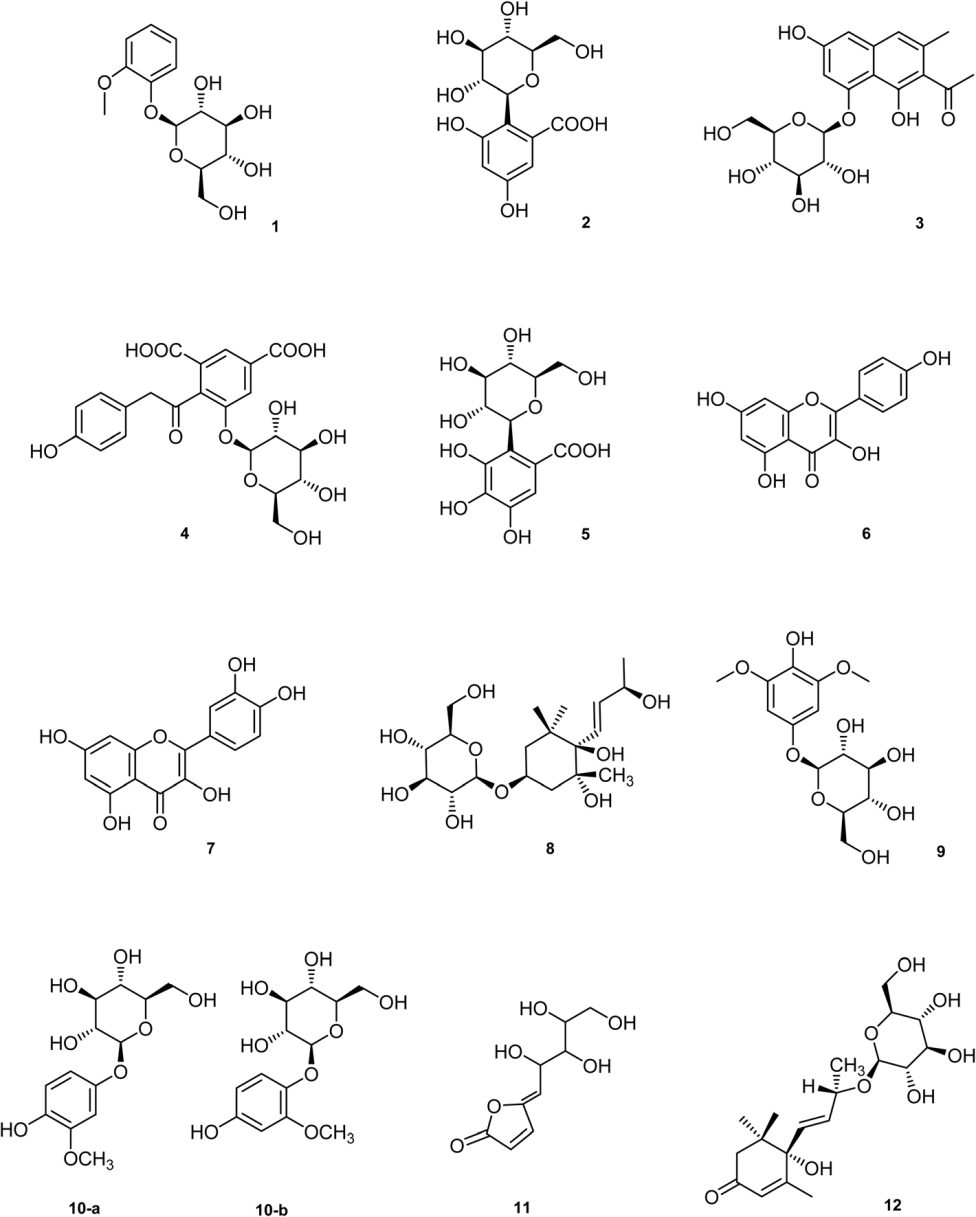
Chemical structures of compounds (1–12).

Herein, our ongoing investigation into active specialized metabolites describes the isolation and structure elucidation of 12 compounds (1–12) as seen in Fig. 1, among them 2 and 5 are undescribed phenolic *C*-glycosides. We then utilized molecular docking for virtual anticancer and anti-quorum sensing screening. This involved predicting the LSD1 and QS inhibitory activity of the isolates obtained from the aerial parts of *A. elliptica* Thunb. to justify their reported traditional use.

## 2. Experimental

### 2.1. General experimental procedures

Column chromatography (CC) was performed on silica gel (0.04–0.063 mm, Merck), Diaion® (HP-20 resin, Sigma-Aldrich), Solid phase extraction (SPE) cartridge (C18) reversed-phase silica gel (Polarbond, JT Baker), and Sephadex LH-20 (0.25–0.1 mm, Aldrich), with analytical grade solvents from Fisher Scientific. TLC was carried out on silica gel 60 F_254_ (0.2 mm, Merck) pre-coated aluminum sheets. The visualization was done by spraying with 5% vanillin (Sigma) solution in conc. H_2_SO_4_–EtOH (5 : 95) followed by heating. NMR experiments were conducted on 500 (^1^H) and 125 MHZ (^13^C) on a BrukerAvance DRX-500 instrument and at 400 (^1^H) and 100 MHz (^13^C) on a Mercury 400 MHz variant spectrometer using CD_3_OD or DMSO-*d*_6_ (Sigma-Aldrich). HR-ESI-MS were performed on BrukerBioApex-FTMS with electrospray ionization (ESI) using loop injection method. IR spectra were recorded using the Thermo Scientific Nicolet 6700 Fourier Transform Infrared Spectrometer (FTIR). Optical rotations were acquired at room temperature using a PerkinElmer automatic precision polarimeter 341.

### 2.2. Plant material

The aerial parts of *Ardisia elliptica* Thunb (https://www.worldfloraonline.org/taxon/wfo-0000544255) were collected during its flowering stage in August 2019, from Mazhar's Botanical Garden, Nahia, Imbaba, Giza, Egypt. It was identified by the Agricultural engineer Terase Labib, consultant of plant taxonomy at the Ministry of Agriculture and director of El-Orman Garden, Giza, Egypt who was a specialist in Plant Taxonomy. A voucher specimen (AE-2019) was deposited at the Herbarium of Pharmacognosy Department, Faculty of Pharmacy, Assiut University, Assiut, Egypt.

### 2.3. Extraction and isolation

The air-dried plant material of *A. elliptica* (2.5 kg) was ground and macerated in 70% ethanol (5 × 5 L, 24 h each) at 25 °C. The combined ethanolic extracts were concentrated on a Büchi Rotavapor under reduced pressure at 45 °C to give a dry residue (80 g) which was dissolved in distilled water (1 L) and partitioned against *n*-hexane (5 × 1 L) followed by dichloromethane (DCM) (5 × 1 L), then ethyl acetate (EtOAc) (5 × 1 L) to yield three major fractions; *n*-hexane fraction (35 g), dichloromethane fraction (15 g) and EtOAc fraction (6.5 g). The remaining aqueous fraction was dried (Büchi Rotavapor under high vacuum, 5 g). Then the residue was dissolved in distilled water (500 mL), filtered through a cotton-wool pad, and passed through a Diaion® HP-20 resin column (5 × 150 cm, 600 g) eluted successively with H_2_O, 25% methanol (MeOH) (in water), 50% MeOH, 75% MeOH and 100% MeOH.

The EtOAc fraction (6.5 g) was chromatographed over Sephadex LH-20 (100 × 2.7 cm, 75 g) using MeOH [1 L] to yield ten subfractions I–X. Subfraction III (900 mg) was applied on silica gel CC (105 × 1.7 cm, 25 g) and eluted with DCM-MeOH [10 : 0, 9.5 : 0.5 and 9 : 1, (1 L, each)] to give five subfractions III.1–III.5. Subfraction III.3 (25 mg) was purified by solid phase separation using an SPE cartridge (C18, 2 g) under vacuum eluted with H_2_O–MeOH [10 : 0, 9.5 : 0.5 and 9 : 1, (100 mL, each)] to yield compound 1 (5 mg). Subfraction IV (1.2 g) was applied on silica gel CC (120 × 1.7 cm, 30 g) and eluted with EtOAC-DCM-MeOH–H_2_O [15 : 8:4 : 1, and 10 : 6 : 4 : 1 (1 L, each)] to give ten subfractions IV.1–IV.10. Subfraction IV.4 (100 mg) was purified by solid phase separation using an SPE cartridge (C18, 10 g) under vacuum eluted with H_2_O–MeOH [10 : 0, 9.5 : 0.5 and 9 : 1 (100 mL, each)] to yield compound 2 (2 mg) and compound 3 (3 mg). Subfraction IV.6 (230 mg) was purified by solid phase separation using an SPE cartridge (C18, 10 g) under vacuum eluted with H_2_O–MeOH [10 : 0, 9 : 1, and 4 : 1 (100 mL, each)] to yield compound 4 (5 mg). Subfraction IV.7 (120 mg) was further purified by solid phase separation using an SPE cartridge (C18, 10 g) under vacuum eluted with H_2_O–MeOH [10 : 0, 9 : 1, and 4 : 1 (100 mL, each)] to yield compound 5 (20 mg). Subfractions VIII and X were crystallized from MeOH to give compound 6 (7 mg) and compound 7 (9 mg), respectively.

The 25% MeOH (in water) fraction (2 g) was chromatographed over Sephadex LH-20 (100 × 2.7 cm, 50 g) using MeOH [1 L] to yield eight subfractions I–VIII. Subfraction II (40 mg) was purified by solid phase separation using an SPE cartridge (silica gel, 2 g) under vacuum eluted with DCM-MeOH [10 : 0, and 9 : 1 (100 mL, each)] to yield compound 8 (7 mg). Subfraction III (50 mg) was applied on normal silica gel CC (70 × 1.2 cm, 1.5 g), eluted with EtOAC-DCM-MeOH–H_2_O [15 : 8:4 : 1, and 10 : 6:4 : 1 (1 L, each)] to yield compound 9 (12 mg). Subfraction V (25 mg) was subjected to solid phase separation using an SPE cartridge (silica gel, 2 g) under vacuum eluted with DCM-MeOH [9.5 : 0.5, 9 : 1, and 8.5 : 1.5 (500 mL, each)] to give three subfractions V.1–V.3. Subfraction V.3 (6 mg) was purified on sephadex LH-20 (20 × 1 cm, 1.8 g) and eluted with MeOH (500 mL) to yield a mixture of compounds 10-a and 10-b (6 mg). Subfraction VI (30 mg) was purified on normal silica gel CC (70 × 1.2 cm, 1 g), eluted with EtOAC-DCM-MeOH–H_2_O [15 : 8 : 4 : 1, and 10 : 6 : 4 : 1 (500 mL, each)] to yield compound 11 (12 mg).

The 50% MeOH (in water) fraction (2.5 g) was chromatographed over Sephadex LH-20 (100 × 2.7 cm, 50 g) using MeOH [1 L] to yield ten subfractions I–X. Subfraction II (700 mg) was applied on normal silica gel CC (100 × 1.8 cm, 18 g), eluted with EtOAC-DCM-MeOH–H_2_O [15 : 8:4 : 1, and 10 : 6:4 : 1 (1 L, each)] to yield compound 12 (10 mg).

### 2.4. Molecular docking

Molecular modeling docking simulations were conducted using Molecular Operating Environment (MOE, 2020.09) software. The X-ray crystallographic structure of LSD1 (PDB, ID: 5YJB) and the quorum regulator protein of *Chromobacterium violaceum*, CviR (PDB, ID: 3QP5) was downloaded from the RCSB protein data bank. The method is detailed in the supplementary material.

## 3. Results and discussion

### 3.1. Structural elucidation of isolated compounds

**Fig. 2:**
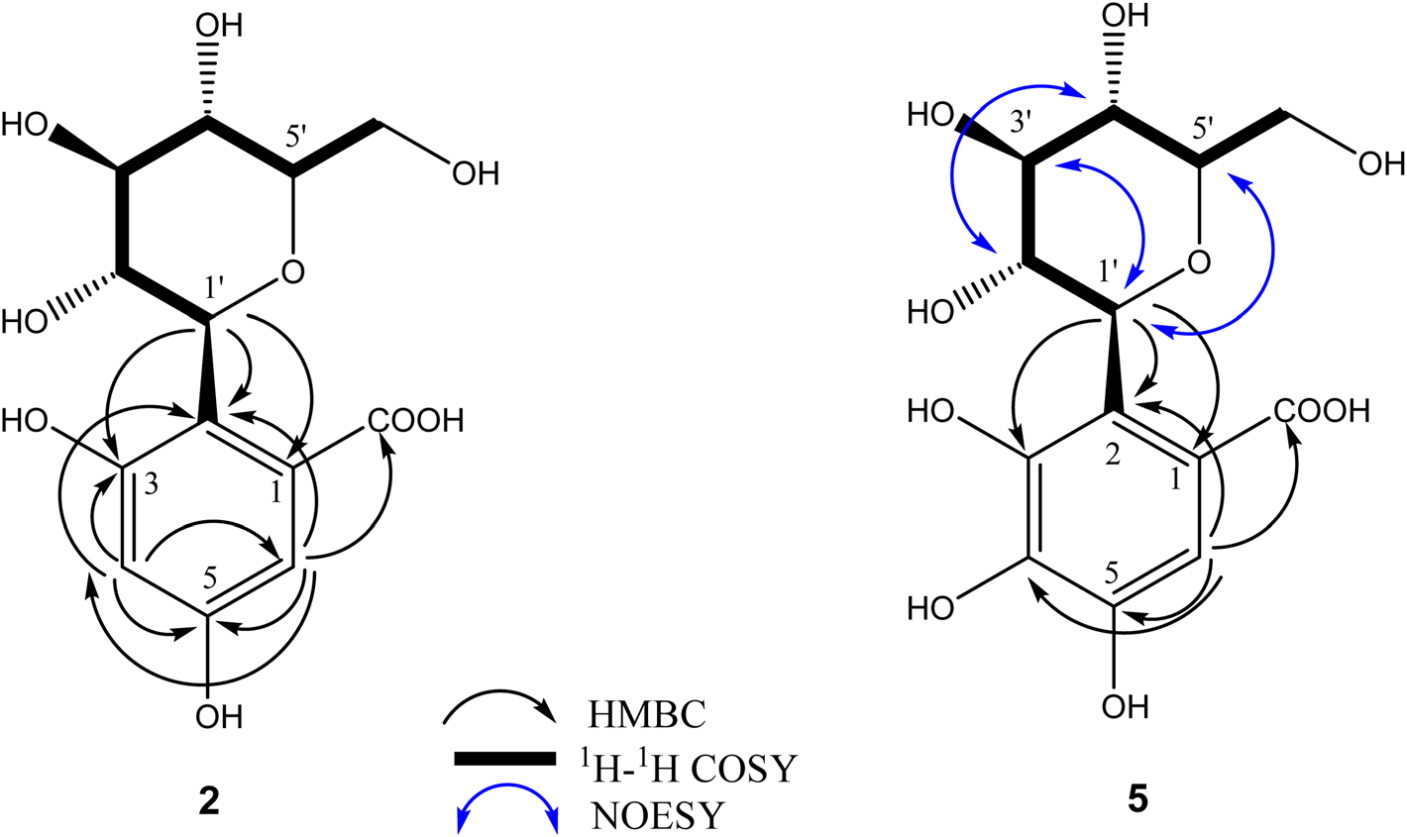
Key HMBC, ^1^H–^1^H COSY, and NOESY correlations of compounds 2 and 5.

Compound 2 was obtained as yellowish residue, [α]^20^_D_-17 (c 1.0, MeOH) and displayed a deprotonated pseudo-molecular ion [M–H]^−^ at *m*/*z* 315.0706 (calcd: 315.0716) in the negative mode HR-ESI-MS, corresponding to a molecular formula of C_13_H_15_O_9_, error = 3.2 ppm. IR analysis (KBr disc) 3531 (–OH phenolic), 3345 (–OH carboxylic), 1701 (C=O carboxylic), and 1621 (C=C aromatic) (Fig. 40S). The ^1^H and ^13^C NMR data (Table 1) were characterized by the presence of two aromatic proton signals at *δ*_H_ 6.48 (d, *J* = 2 Hz)/*δ*_C_ 108.8 and *δ*_H_ 6.87 (d, *J* = 2 Hz)/*δ*_C_ 108.4 and one carboxylic carbon at *δ*_C_ 163.5 suggesting the presence of trisubstituted benzoic acid moiety. In addition to the presence of two signals attributed to oxygenated quaternary carbons at *δ*_C_ 155.5 and 158.7. Additionally, the spectra revealed the presence of a *C*-glucosyl moiety, characterized by an anomeric proton signal at *δ*_H_ 4.93 (d, *J* = 10.4 Hz) and its corresponding carbon signal at *δ*_C_ 72.0. Unlike *O*-glycosides, which typically exhibit anomeric proton signals at lower chemical shifts (*δ*_H_ 4.0–4.5) with smaller coupling constants (*J* ≈ 7–9 Hz) and anomeric carbon signals around *δ*_C_ 100–105, *C*-glycosides display higher anomeric proton shifts and larger coupling constants, along with upfield-shifted anomeric carbon signals (∼*δ*_C_ 70–80) owing to the direct C–C bond between the sugar and aglycone.^37^ The attachment of the glucose unit was suggested to be at C-2 revealed from the ^3^*J* HMBC correlations (Fig. 2 and 4S) of H-1′ at *δ*_H_ 4.93 with C-1 at *δ*_C_ 124.8, C-3 at *δ*_C_ 155.5 and ^2^*J* HMBC correlation with C-2 at *δ*_C_ 114.3. The full assignment of sugar protons was confirmed by the ^1^H ^1^H COSY spectrum (Fig. 5S) and comparison with reported data of closely related *C*-glucosides.^38^ The structure was further confirmed by the ^2^*J* and ^3^*J* HMBC correlations; H-4 (*δ*_H_ 6.48) showed ^3^*J* correlations with C-6 (*δ*_C_ 108.4), C-2 (*δ*_C_ 114.3), and showed ^2^*J* correlations with C-3 (*δ*_C_ 155.5), and C-5 (*δ*_C_ 158.7); and H-6 (*δ*_H_ 6.87) showed ^3^*J* correlations with C-4 (*δ*_C_ 108.8), C-2 (*δ*_C_ 114.3), and carboxylic carbon (*δ*_C_ 163.5) (Fig. 2). Thus, the structure of compound 2 was established and named as *α*-resorcylic acid-2-*C-β*-d-glucopyranoside.

**Table 1:** ^1^H and ^13^C NMR data of compounds 2 and 5 (DMSO-*d*_6_, *δ* in ppm, *J* in Hz)

Position	2		5	
	*δ*_C_	*δ*_H_	*δ*_C_	*δ*_H_
1	124.8	—	113.0	—
2	114.3	—	116.4	—
3	155.5	—	142.7	—
4	108.8	6.48 (d, 2.0)	140.0	—
5	158.7	—	146.2	—
6	108.4	6.87 (d, 2.0)	109.7	7.00 (s)
1'	72.0	4.93 (d, 10.4)	72.5	4.93 (d, 10.4)
2'	79.8	3.97 (t, 9.9)	80.1	3.94 (t, 9.9)
3'	73.7	3.65 (t, 8.9)	74.0	3.67 (t, 8.9)
4'	70.7	3.19 (m)	71.1	3.22 (t, 9.0)
5'	81.7	3.55 (m)	81.8	3.57 (d, 8.9)
6'	61.1	3.83 (d, 11.0)	61.5	3.85 (d, 11.0)
		3.43 (dd, 11.8, 7.5)		3.45 (dd, 11.5, 7.5)
CO	163.5	—	164.2	—

Compound 5 was obtained as yellowish residue, [α]^20^_D_-12 (c 0.5, MeOH), showed a dehydrated pseudo-molecular ion peak [M–H_2_O + Na]^+^ at *m*/*z* 337.0537 (calcd 337.0536) in the positive ion mode, corresponding to a molecular formula of C_13_H_14_O_9_Na, error = 0.3 ppm. IR analysis (KBr disc) 3600 (–OH phenolic), 3303 (–OH carboxylic), 1708 (C=O carboxylic), and 1621 (C=C aromatic) (Fig. 41S).

The ^1^H and ^13^C NMR spectra differed only from the previous compound 2 in the presence of one singlet aromatic proton H-6 at *δ*_H_ 7.00 correlated by HSQC to a methine carbon at *δ*_C_ 109.7. In addition to the appearance of a quaternary carbon at *δ*_C_ 140.0 corresponding to C-4 in the DEPTQ-135 NMR spectrum (Table 1 and Fig. 8S). The position of the aromatic proton was confirmed to be at C-6 from the HMBC correlations between H-6 (*δ*_H_ 6.97) with the carboxylic carbon at *δ*_C_ 164.2 and all the aromatic carbons C-1, C-2, C-4, and C-5 (*δ*_C_ 113.0, 116.4, 140.0, and 146.2, respectively) except C-3 (*δ*_C_ 142.7) (Fig. 2). Moreover, the anomeric proton at *δ*_H_ 4.93 (d, *J* = 10.4 Hz) displayed HMBC correlations with C-1 (*δ*_C_ 113.0), C-2 (*δ*_C_ 116.4), and C-3 (*δ*_C_ 142.7) (Fig. 2 and 10S). Additionally, NOESY spectrum revealed correlations from the α- oriented H-1′ to both H-3′ and H-5′ and from H-4′ to H-2′, confirming the presence of a *β*-glucosyl moiety (Fig. 2 and 11S). Therefore, compound 5 was identified to be gallic acid-2-*C-β*-d-glucopyranoside.

Ten known compounds were identified (Fig. 1) as 2-methoxyphenyl *β*-d-glucopyranoside (guaiacol *β*-glucoside) (1),^39^ 6-hydroxymusizin (3),^40^ soulieana acid (4),^41^ kaempferol (6),^42^ quercetin (7),^43^, (3R, 5R, 6R, 7E, 9S)-megastigman-7-ene-3,5,6, 9-tetrol-3-*O-β*-d-glucopyranoside. (8),^44^ 2, 6-dimethoxy-4-hydroxyphenol-1-*O-β*-d-glucopyranoside (9),^45^ the (3 : 2) mixture of tachioside (methoxyhydroquinone-4-*O-β*-d-glucopyranoside) (10a)/isotachioside (methoxyhydroquinone-1-*O-β*-d-glucopyranoside) (10b),^46^ litchiol B (11),^47^ and corchoionoside C (12),^48^ based on NMR data (Fig. 13S–22S), as well as by comparison to the spectral data already reported in literature.

### 3.2. Molecular docking

Natural products continue to provide innovative scaffolding for the development of new LSD1 inhibitors and quorum sensing inhibitors. This study virtually screens natural LSD1 and QS-inhibitors, compounds (1–12), for their potential anticancer and anti-quorum sensing activities. Our research focuses on the co-crystal structures of the LSD1 and CviR QS regulator protein/natural isolates complex and explores their mechanisms of action.

#### 3.2.1. Docking as lysine-specific demethylase 1 (LSD1/KDM1A) inhibitors

Based on previous findings, molecular docking studies were performed to virtually assess their potentiality as antitumor by predicting the possible binding mode between isolated compounds and LSD1 using the crystal structure of LSD1 (PDB code: 5YJB) as a target protein. The docking information for the isolated compounds is displayed in Table 2, together with the binding energy score (S), types of ligand-5YJB amino acid interactions, and intermolecular distances (Å). Binding interactions of isolated compounds with key residues showed comparable binding pattern to reference ligand with docking affinities ranging from −12 to −9.5 kcal mol^−1^ (co-crystalized substrate −11.4 kcal mol^−1^).

**Table 2:** *In silico* outcomes of the isolated compounds (1–12) on LSD1

Compounds	*S* (kcal mol^−1^)	Sites of interactions	Interaction types distance (Å)
Cocrystalized ligand	−11.4	LYS 661	H-acceptor (2.2)
		ASP 555	H-donor (2.01)
		MET 332	Ionic (3.41)
		VAL 333	H-donor (2.44) pi–H (3.66)
1	−9.7	ALA 539	H-donor (3.82)
		ASP 555	H-donor (3.93)
			H-donor (3.98)
2	−10.3	ALA 539	H-donor (2.97)
		LYS 661	H-acceptor (3.03)
		MET 332	H-acceptor (3.37)
		VAL 333	H-acceptor (3.23)
3	−11.2	VAL 811	H-acceptor (2.05)
		LYS 661	H-acceptor (2.22)
4	−12	ARG 316 ALA 331	H-acceptor (2.19) pi–H (3.08)
		MET 332	H-donor (2.25)
		VAL 333	H-donor (2.15)
		LYS 661	H-acceptor (2.78)
			H-acceptor (2.93)
5	−9.5	ASP 555	H-donor (2.75)
			H-donor (3.49)
6	−10.2	ALA 539	H-donor (2.84)
		ASP 555	H-donor (2.89)
		VAL 333	pi–H (3.42)
7	−10	MET 332	H-donor 3.59
		ASP 555	H-donor 2.93
		HIS 564	H-acceptor 3.23
8	−10.9	ASP 555	H-donor (2.30)
		MET 332	H-acceptor (2.25)
		VAL 333	H-acceptor (2.3)
9	−10.6	ASP 555	H-donor (2.88)
		VAL 811	H-acceptor (2.02)
		MET 332	H-acceptor (2.14)
		VAL 333	H-acceptor (2.98)
10-a	−10.8	ASP 555	H-donor (2.97)
			H-donor (2.88)
		MET 332	H-acceptor (3.20)
		VAL 333	pi–H (3.74)
10-b	−10	MET 332	H-donor (2.41)
		MET 332	H-donor (2.57)
		LYS 661	H-acceptor (2.30)
		THR 810	pi–H (3.95)
11	−10.2	MET 332	H-donor (3.61)
		MET 332	H-acceptor (3.14)
		VAL 333	H-acceptor (3.23)
		LYS 661	H-acceptor (3.25)
12	−10.4	ASP 555	H-donor (2.18)
		SER 762	H-acceptor (3.22)
		Val 764	H-acceptor (2.63)

**Fig. 3:**
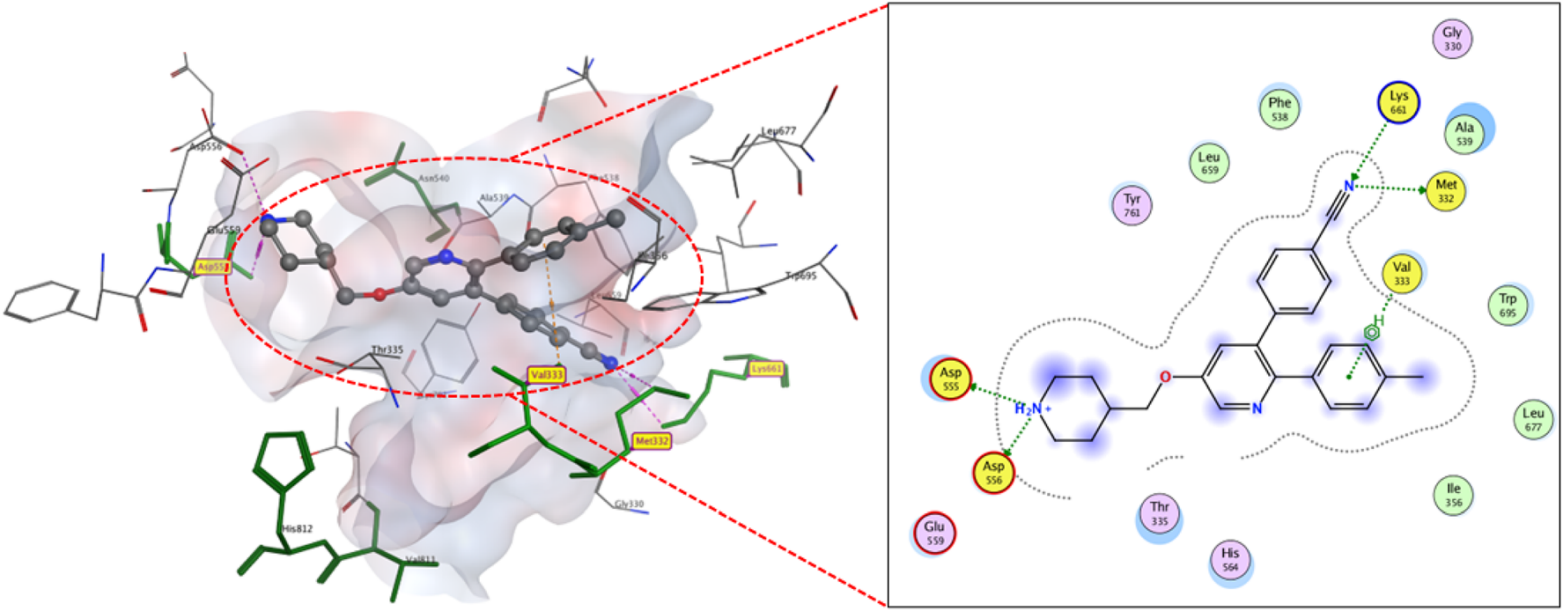
2D and 3D display of docking interactions of native ligand against LSD1.

Fig. 3 emphasizes the most valuable sites of the targeted protein and provides the corresponding primary docking parameters of the control native ligand; 4-[5-(piperidin-4-ylmethoxy)-2-(*p*-tolyl) pyridin-3-yl] benzonitrile. The docking score (*S*) values and the number of hydrogen bonds are selected as fundamental indicators for inhibitory efficacy. The former correlates to Gibbs free energy of the inhibition, whereas the latter indicates strong intermolecular bindings.

**Fig. 4:**
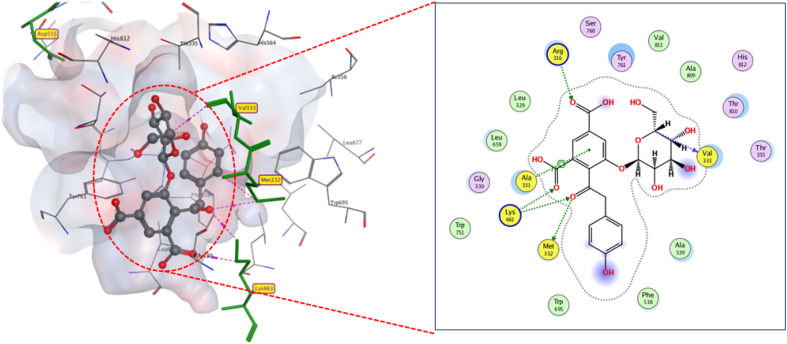
2D and 3D display of docking interactions of compound 4 against LSD1.

In general, isolated compounds exhibit different affinities towards the protein active sites (either *S* values or number of hydrophilic interactions). LSD inhibitors can be ranked concerning their predicted potency on average in the following order: compound 4 (*S* −12 kcal mol^−1^) > reference inhibitor and compound 3 (*S* −11.2 kcal mol^−1^) > compounds 8, 9 and 10-a (*S* −10.9–10.6 kcal mol^−1^) > compounds 10-b, 2, 12, 11, 6 and 7 (*S* ≈ −10.3 kcal mol^−1^) > compounds 1 and 5 (*S* ≈ −9.5 kcal mol^−1^). According to docking interpretation, compound 4 exhibited the best binding affinity to the LSD-active site expressed through the highest binding score −12 kcal mol^−1^ and its binding mode. As depicted in (Fig. 4) the increased activity of compound 4 could be correlated to the favorable interactions with the active site residue LYS661 which is a key residue in the lysine demethylation catalytic reaction of LSD1. Compound 4 acidic carbonyl groups interacted with ARG316 and LYS661 through hydrogen bonds with bond lengths 2.19 and 2.78, respectively. Additionally, ketonic carbonyl forms two 2.33 and 2.25 Å hydrogen bonds with the side chain of LYS661 and MET332. Moreover, bounding is supported by Val333-hydrogen bonding (2.15 Å) and hydrophobic stacking with ALA331. These interactions with the LSD1-binding region are extensive and type-rich, potentially explaining the boosted docking score.

Compound 3 fits the active site (docking score −11.2 kcal mol^−1^) with two firmly hydrogen bonds, one between the ketonic carbonyl and LYS661 (2.05 Å). The other lies between the phenolic hydroxyl moiety and VAL811 (2.2 Å), as shown in (Fig. 24S).

**Fig. 5:**
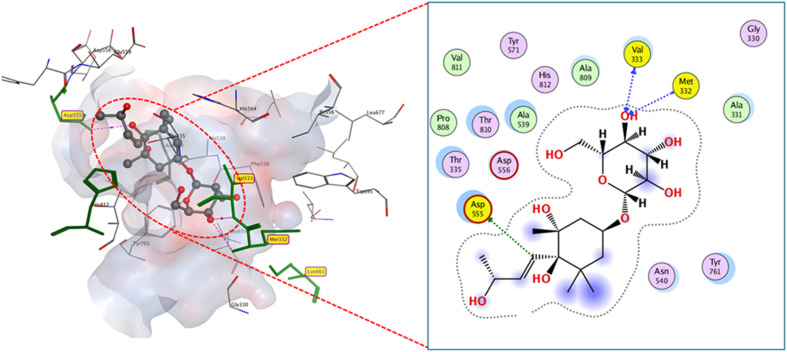
2D and 3D display of docking interactions of compound 8 against LSD1.

Compounds 8, 9 and 10-a docked with moderate binding scores relative to native ligand (≈10.8 kcal mol^−1^) despite their distinct binding modes. Concerning compound 8, Fig. 5, which reacted with ASP555, MET332, and Val333 to occupy the LSD1-active region through three hydrogen bonds with favorable bond lengths (2.30, 2.25, 2.3 Å). The two methoxy groups in compound 9 permit change in-site orientation and electron density phenolic moiety resulting in creating 2.88 Å hydrogen bond with ASP555 and sugar hydroxyl hydrogen bound to VAL811, MET332 and VAL333 (2.02, 2.14, and 2.98 Å consequently). In contrast, compound 10-a showed hydrophobic stacking with phenolic moiety and VAL333 in addition to phenolic hydroxyl being connected to MET332 (3.20 Å) and hydroxyl groups to ASP555 through two hydrogen bonds (2.57 and 2.38 Å), (Fig. 26S and 27S).

Compound 12 established three hydrogen bonds with key active site residues ASP555, SER762, and VAL762, exhibiting bond lengths of 2.18 Å, 3.22 Å, and 2.63 Å, respectively, with reduced docking affinity (*S* −10.4 kcal mol^−1^). Compound 11-side chain hydroxyl groups established four hydrogen bonds with MET 332, VAL333, and LYS 661 LSD1-active residues (Fig. 29S and 30S). It had a reduced binding score of (*S* −10.2 kcal mol^−1^) due to weak hydrogen bonds (3.61, 3.14, 3.23, and 3.25 Å, respectively) even with multiple hydrogen bonds. Compound 10-b revealed the binding of its hydroxyl entities to LYS661 and THR810 (2.3 and 3.95 Å), as well as the phenyl-MET332 hydrophobic interaction with unpredictable unbinding to the phenolic hydroxyl group as a result of the change in the orientation for energetic stability (*S* −10 kcal mol^−1^).

**Fig. 6:**
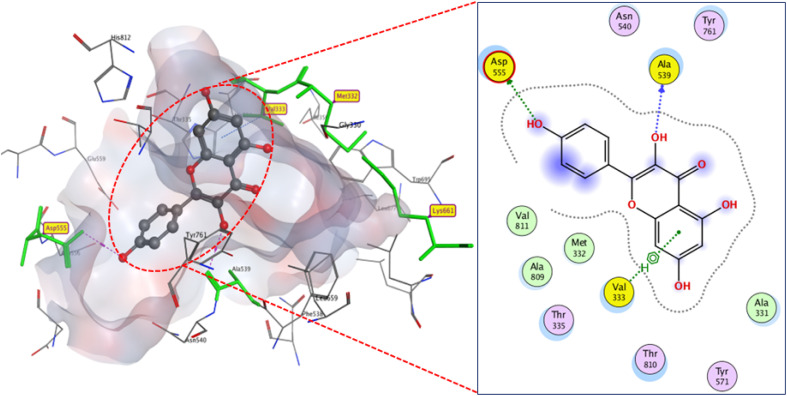
2D and 3D display of docking interactions of compound 6 against LSD1.

**Fig. 7:**
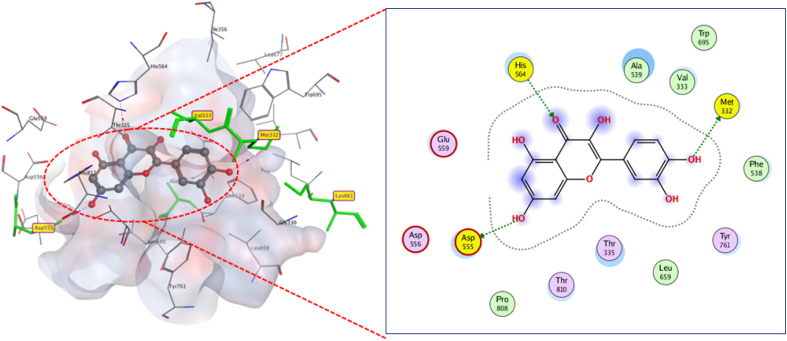
2D and 3D display of docking interactions of compound 7 against LSD1.

Flavonoids 6 and 7 docked to binding region with comparable binding scores (*S* −10.2 and −10 kcal mol^−1^) but distinct modes. Compound 6 connected to active residue ASP555 (2.89 Å) *via* hydrogen bonding and showed hydrophobic stacking to VAL333, with extra hydrogen bonding to ALA 539. Meanwhile compound 7 formed three hydrogen bonds: two between active residues (MET332 (3.5 Å) and ASP555 (2.9 Å)) and carbonyl and hydroxyl groups. The third one is formed between its hydroxyl group and HIS564 (3.2 Å) (Fig. 6 and 7).

**Fig. 8:**
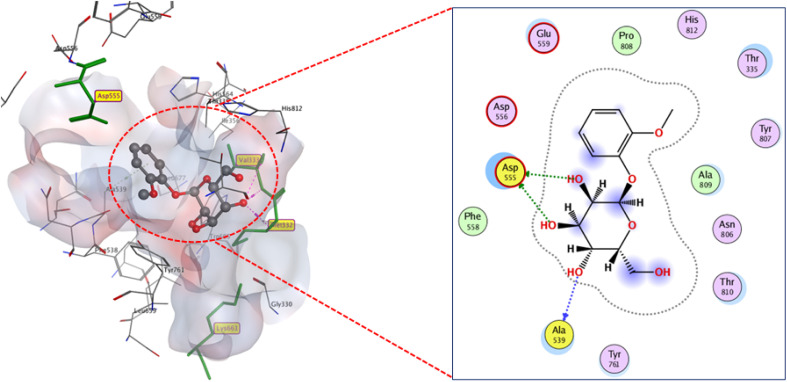
2D and 3D display of docking interactions of compound 1 against LSD1.

Despite having a carboxylic moiety, compounds 2 and 5 docked to the active site in different ways and with different scores. The reason behind this could be the hydroxyl groups found in compound 5, which promote intramolecular hydrogen bonding instead of intermolecular ones with the active residues. Thus, compound 5 formed only two weak hydrogen bonds (2.75 and 3.49 Å) with ASP55, resulting in a poor binding score of (*S* −9.5 kcal mol^−1^), comparatively, compound 2 fitted the binding site with a docking score −10.3 kcal mol^−1^ by forming four hydrogen bonds with ALA539, LYS661, MET332, and VAL333 (2.97, 3.03, 3.37, and 3.23 Å) (Fig. 23S). Compound 1 displayed docking score coherent to compound 5, which is virtually predicted as having the lowest LSD1 inhibitory activity among the isolated compounds. Compound 1 docked in the active pocket through two weak hydrogen bonds with ASP555 (3.93 and 3.98 Å, respectively), in addition to hydrogen bonding with ALA539 (3.83 Å), which permitted poor binding to active residues (Fig. 8).

#### 3.2.2. Docking as quorum sensing inhibitor

*Chromobacterium violaceum* is a Gram-negative bacterium frequently used in QS investigations. Upon the presence of autoinducers, which are small signal molecules, bacteria can employ QS to regulate the expression of specific genes. *N*-acyl homoserine lactones (AHL), such as C6-HSL, are the autoinducers in Gram-negative bacteria. When autoinducers reach a certain concentration threshold, they interact with the transcriptional regulators and alter gene expression patterns.^49^

The researchers focused on looking for chemical entities that mimic AHLs. These are predicted to block the signal receptor, synthase, or both. AHL-like substances can permeate across Gram-negative bacterial cell membranes and function primarily on QS. Natural products have garnered attention for their therapeutic effects in traditional medicine. They have the ability to function as anti-QS on pathogenic bacteria.^50^ Computational methods, such as molecular docking, can anticipate a biomolecular approach to the inhibitory interactions of antimicrobial drugs against certain enzymes in QS.

Our current work virtually highlighted the ability of isolated compounds to inhibit QS in a QS biosensor strain) *C. violaceum* using *in silico* molecular docking. Chlorolactone (CL), a potent QS antagonist, binds to the autoinducer active pocket and inhibits DNA binding by competing with C6-HSL. Simply, it binds to C6-HSL site on CviR QS regulator protein enhances its DNA binding and activating transcription, whereas binding to CL greatly enhances the locked, inactive conformation of CviR.^51^

Based on earlier research, 3QP5 is the closed conformation of the CviR protein. The homotetramer version of this protein (A, B, C, and D) and chain A were assigned for the docking study. Docking the isolated compounds into the active pocket of the CviR QS regulator protein (3QP5) revealed binding modes resembling that of CL, a potent protein inhibitor, as indicated in (Table 3).

**Table 3:** *In silico* results of the isolated compounds (1–12) on CviR QS regulator protein

Compounds	*S* (kcal mol^−1^)	Sites of interactions	Interaction types distance (Å)
Cocrystalized ligand	−13.3	ASP 97	H-donor (2.85)
		TRP 84	H-acceptor (2.94)
		TYR 80	H-acceptor (2.76)
		SER 155	H-acceptor (3.33)
1	−11.8	ASP 97	H-donor (3.04)
		TYR 80	H-acceptor (2.70)
		SER 155	H-acceptor (3.00)
		TYR 88	pi–pi (3.83)
2	−12.2	ASP 97	H-donor (2.09)
		SER 155	H-acceptor (2.70)
		TYR 80	H-acceptor (2.84)
		TRP 84	H-acceptor (3.00)
		TYR 88	pi–pi (3.65)
3	−10.3	ASP 97	H-donor (3.13)
			H-donor (2.66)
		TYR 80	H-acceptor (2.99)
		SER 155	H-acceptor (2.66)
		TYR 88	pi–pi (3.48)
4	−10.6	ASP 97	H-donor (2.34)
		TYR 80	H-acceptor (2.48)
		SER 155	H-acceptor (2.55)
5	−11.2	ASP 97	H-donor (3.16)
		TYR 80	H-acceptor (2.80)
		TRP 84	H-acceptor (3.15)
		TYR 88	pi–pi (2.98)
6	−8.5	TRP 84	H-acceptor (3.10)
		VAL 75	pi–H (4.19)
7	−8.8	ASP 97	H-donor (2.85)
		TYR 80	pi–H (3.96)
8	−13	ASP 97	H-donor (2.16)
		TRP 84	H-acceptor (2.22)
		SER 155	H-acceptor (2.07)
		MET 89	H-acceptor (2.19)
9	−8.4	ASP 97	H-donor (3.13)
		TYR 88	pi–pi (3.82)
10-a	−11.8	ASP 97	H-donor (2.03)
		TYR 80	H-acceptor (2.30)
		SER 155	H-acceptor (2.13)
		TYR 88	pi–pi (3.34)
10-b	−12.2	ASP 97	H-donor (3.15)
		TYR 80	H-donor (3.18)
		SER 155	H-acceptor (2.72)
		TYR 88	H-acceptor (2.99) pi–pi (3.82)
11	−9.5	SER 155	H-acceptor (3.22)
		TRP 84	H-acceptor (2.77)
12	−11	ASP 97	H-donor (2.99)
		TRP 84	H-acceptor (2.75)
		TYR 80	H-acceptor (2.75)
		SER 155	H-acceptor (2.58)

**Fig. 9:**
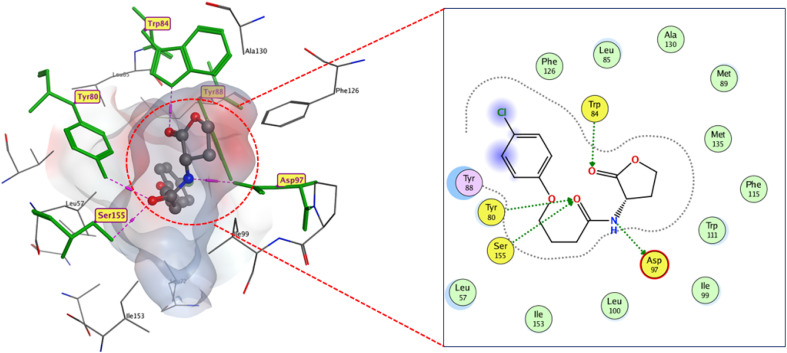
2D and 3D display of docking interactions of native inhibitor CL against CviR QS protein.

Analysis of docking outcomes revealed that CL has four critical interaction points: the lactone carbonyl group, the acyl amine group, and the lactone carbonyl oxygen, which connects with TRP84, ASP97, TYR80, and SER155, consequently, through H-bonds, which is consistent with earlier findings, with a binding score of −13.3 kcal mol^−1^ (Fig. 9).

Isolated compounds displayed docking mode in great agreement with the native inhibitor. It was found that the sugar part participates in most of the hydrogen bonding between the docked compounds and active residues. In terms of average anticipated potency, quorum sensing inhibitors can be ranked as follows: compound 8 and reference inhibitor (*S* ≈ −13 kcal mol^−1^) > compounds 1 = 10-a, and 2 (*S* ≈ −12 kcal mol^−1^) > compounds 5 and 12 (*S* ≈ −11 kcal mol^−1^) > compounds 3, 4, and 10-b (*S* ≈ −10.4 kcal mol^−1^) > Compound 6, 7, 9, and 11 (*S* ≈ −9 kcal mol^−1^).

**Fig. 10:**
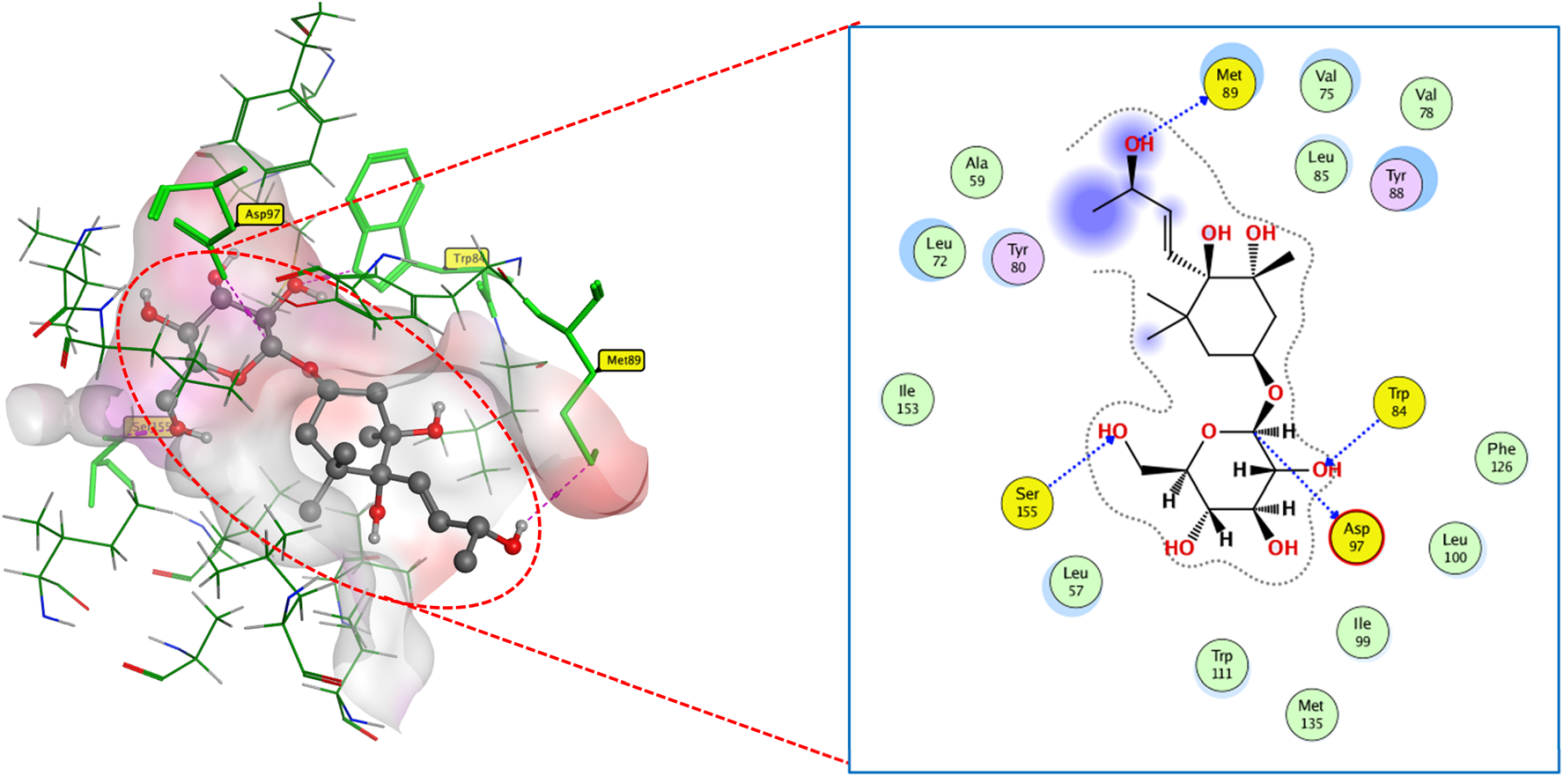
2D and 3D display of docking interactions of compound 8 against CviR QS protein.

As presented in (Fig. 10), compound 8 engages the active site residues in a binding mode comparable to that of the native ligand. It forms hydrogen bonds between its hydroxyl groups and residues ASP97, TRP84, and SER155, with an additional hydrogen bond with MET89. This binding configuration, supported by a docking score of −13.0 kcal mol^−1^, indicates a strong affinity for the target site. The hydrogen bond lengths reflect the elevated compound-protein affinity, resulting in a higher docking score.

**Fig. 11:**
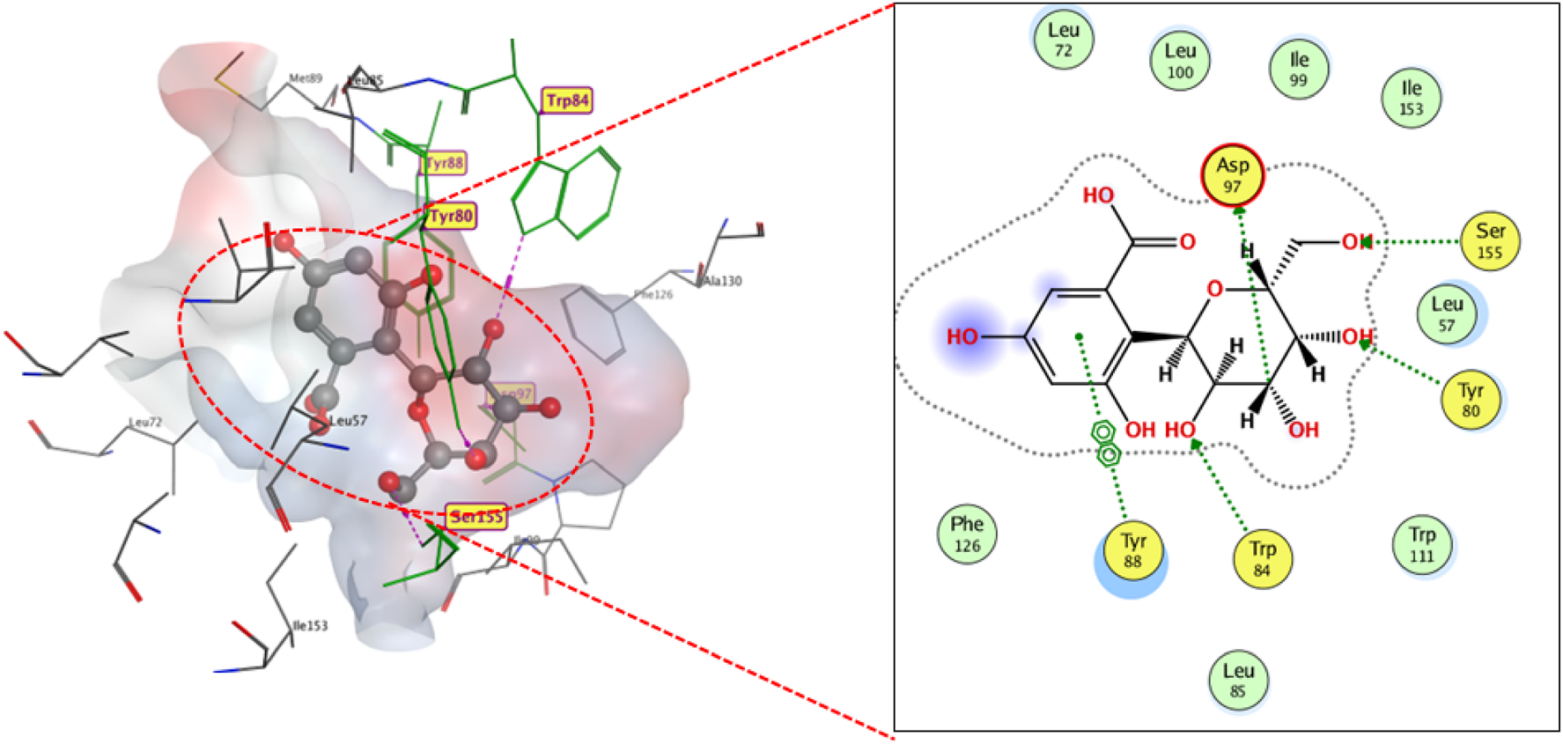
2D and 3D display of docking interactions of compound 2 against CviR QS protein.

Compound 2 demonstrated similar interactions with CviR QS-active residues as compound 8, with one notable difference (Fig. 11). An additional hydrogen bond formed between compound 8 and ASP97, characterized by a short bond length of 2.16 Å. This additional interaction may explain the lower docking score of compound 2 (−12.2 kcal mol^−1^) compared to compound 8 docking score (−13 kcal mol^−1^).

Even while exhibiting a comparable docking score to compound 2, compound 10-a implied distinct binding characteristics within the active site. The hydroxyl groups of compound 10-a linked to ASP97, TYR80, and SER155 with stronger hydrogen bonds (2.03, 2.3, and 2.13 Å, respectively). Furthermore, pi–pi interaction was observed between the hydrophobic phenyl groups and TYR88 (Fig. 35S).

**Fig. 12:**
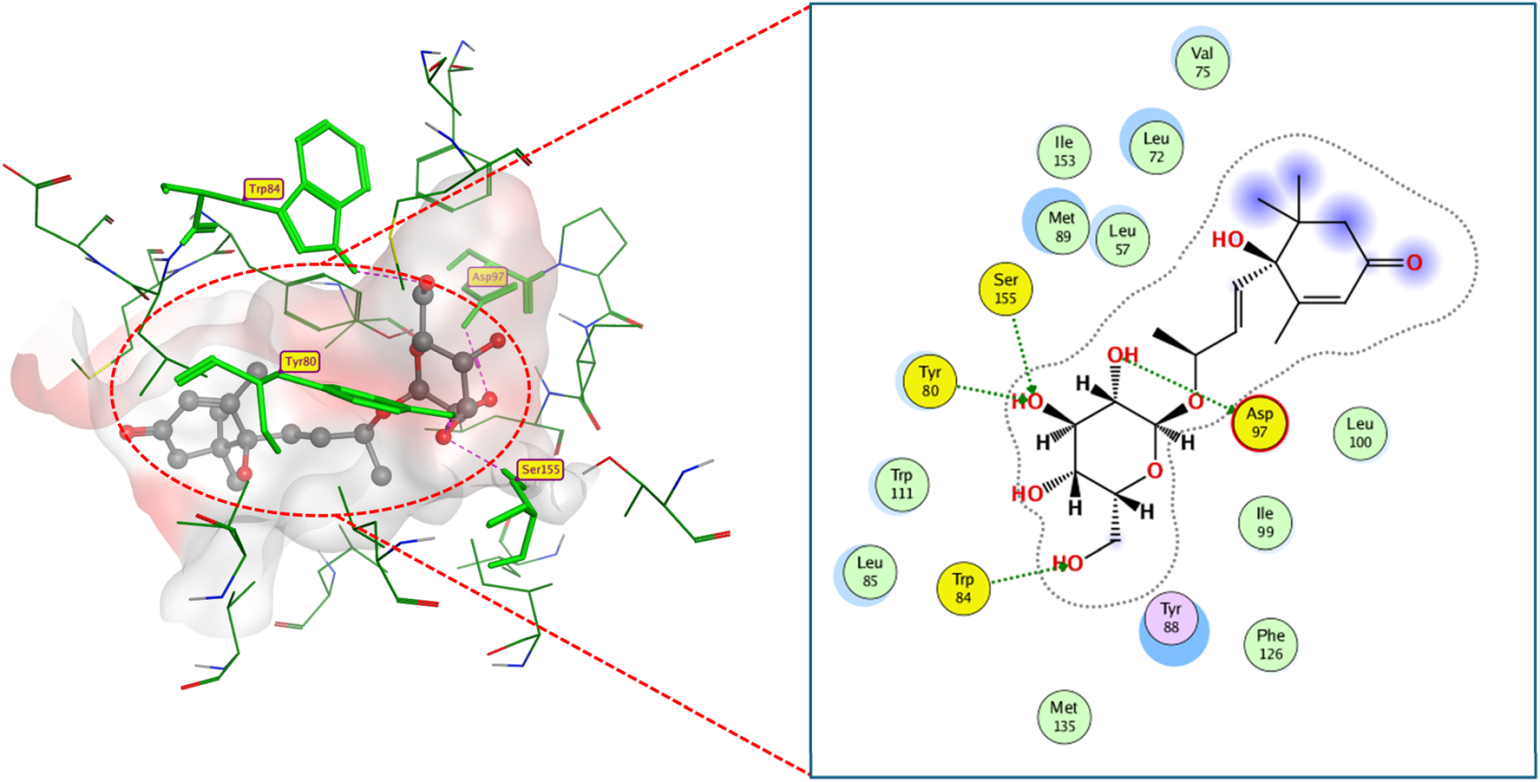
2D and 3D display of docking interactions of compound 12 against CviR QS protein.

Compound 12 interacted with the substrate-binding pocket through hydrogen bonding. Specifically, its hydroxyl groups formed bonds with ASP 97, TYR 80, TRP84, and SER155, with respective bond lengths of 2.99, 2.75, 2.75, and 2.58 Å. The ring carbonyl and hydroxyl groups were not engaged in the binding owing to their orientation away from the active pocket (Fig. 12).

Compound 5 hydroxyl groups were hydrogen-bonded to ASP97, TYR80 and TRP84 (3.16, 2.8, and 3.15 Å, respectively). Additionally, extra hydrophobic binding of a phenyl group to TYR88 led to a moderate docking score (−11.2 kcal mol^−1^). Its orientation resulted in the unexpected non-engagement of carboxylic and hydroxyl phenolic groups in binding (Fig. 34S).

Compound 4 docked only to three key residues: ASP97, TYR80, and SER155 with respective shorter hydrogen bond lengths of 2.34, 2.48, and 2.55 Å. This reflects an increased docking score −10.6 kcal mol^−1^, compared to compounds 3 and 10-b. Compounds 3 and 10-b showed analogous behavior to the native inhibitor and compound 8 toward active residues ASP97, TYR80, SER155, and TYR88. However, the shorter hydrogen bond lengths of compounds 3 and 10-b impacted their lesser binding score.

Despite having hydrogen bonding centers, compounds 6 and 7 exhibited an unexpected docking pattern because they attached loosely to the active residues. The carbonyl moiety of compound 6 only formed a hydrogen bond with TRP84 with a relatively long bond length of 3.3 Å and phenolic moiety was pi–H connected with VAL75 (4.19 Å), resulting in a poor docking score of −8.5 kcal mol^−1^. On the contrary, in the docking of compound 7, phenolic hydroxyl group was hydrogen bonded to ASP79 (2.85 Å) and pi–H connected with TYR80 (3.96 Å), with a docking affinity of −8.8 kcal mol^−1^, (Fig. 38S and 39S).

**Fig. 13:**
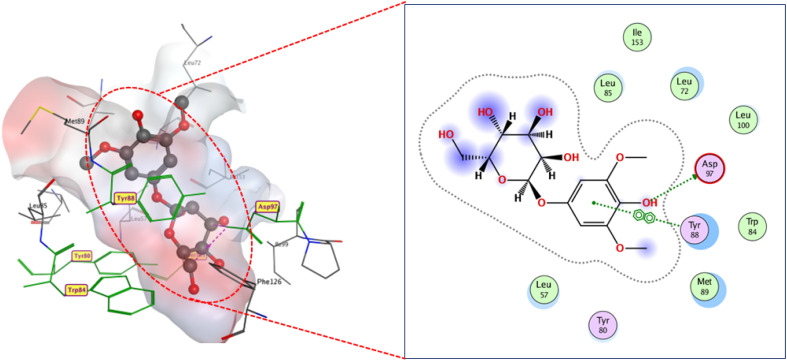
2D and 3D display of docking interactions of compound 9 against CviR QS protein.

Compounds 9 and 11 docked to the active site with lower binding scores (*S* −8.4 and −9.5 kcal mol^−1^, respectively) due to missing interactions with key residues, in contrast to native ligand-CviR QS regulator interactions. Compound 9 bound only to ASP97 key residue through 3.13 Å-hydrogen bonding with its phenolic hydroxyl and to TYR88 through pi–pi interaction with its hydrophobic phenyl moiety (Fig. 13). While compound 11 made two hydrogen bonds (3.22 and 2.77 Å away) with SER155 and TRP 84 (Fig.37S).

These findings support that compounds 4 and 8 could serve as potential leads for developing new antitumor and antibiotic agents. Furthermore, our *in silico* study findings strongly support the traditional use of the plant as an antibacterial and reinforce the results of some studies on *Ardisia* species reporting their cytotoxic effect on HepG2 and HCT116 cell lines, although the exact mechanism is still unknown.^11,52^ Moreover, the isolation and identification of compounds 2 and 5 as new *C*-glycosides derivatives with different interactions to the examined proteins offers a new scaffold for the development of antitumor and antimicrobial agents.

## 4. Conclusion

The ethanolic extract of *A. elliptica* Thunb aerial parts is rich in diverse groups of phytoconstituents, comprising phenolic glycosides, flavonoids, and megastigmanes. The current molecular docking investigation of the isolated compounds predicts the most effective candidate against LSD1 (as an anticancer) and CviR QS protein (as an antimicrobial adjuvant). Regarding LSD1, soulieana acid (4) showed the best docking results, with high affinity for the active pocket, as indicated by a high docking score and stable binding interactions surpassing those of the reference inhibitor. However, in the case of CviR QS protein, (3R, 5R, 6R, 7E, 9S)-megastigman-7-ene-3,5,6, 9-tetrol-3-*O-β*-d-glucopyranoside. (8) exhibited strong affinity for the docking site of the examined protein. Additional *in vitro* and *in vivo* studies are necessary to evaluate their potential applications as LSD1 and quorum sensing inhibitors. Moreover, these scaffolds could serve as valuable leads in the future design of novel and more potent LSD1 and quorum sensing inhibitors.

## Author contributions

All authors participated in the design of the study and helped to draft the manuscript. EMA, LGM, and MA carried out the experimental part (extraction, isolation, and structural elucidation). WSQ carried out the molecular docking part. All authors read and approved of the final manuscript.

## Conflicts of interest

The authors declare that they have no competing financial interests or personal relationships that could have influenced the work reported in this study.

## Abbreviations

AHL: *N*-Acyl homoserine lactones
CC: Column chromatography
CL: Chlorolactone
COSY: Correlation spectroscopy
CviR: Quorum sensing receptor of chromobacterium violaceum
DCM: Dichloromethane
DEPT: Distortionless enhancement by polarization transfer
DMSO: Dimethyl sulfoxide
EtOAC: Ethyl acetate
HMBC: Heteronuclear multiple bond correlation
HR-ESI-MS: High-resolution electrospray ionization mass spectrometry
HSQC: Heteronuclear single quantum coherence
LSD1: Lysine-specific demethylase 1
MeOH: Methanol
NMR: Nuclear magnetic resonance
QS: Quorum sensing
S: Docking score
SPE: Solid phase extraction
TLC: Thin layer chromatography

## Supplementary Material

RA-015-D5RA02005K-s001

## Data Availability

Supplementary information: The data supporting this article have been included as a part of the SI. See DOI: https://doi.org/10.1039/d5ra02005k.
