# Supplementary material for: Insights into LSD1 and quorum sensing inhibitory potential of phytoconstituents isolated from Ardisia elliptica Thunb aerial parts
Source: RSC Adv. 2025 Sep 8;15(39):32057–70. doi: 10.1039/d5ra02005k (PMC12415686; doi:10.1039/d5ra02005k)
Supplement: RA-015-D5RA02005K-s001 [file RA-015-D5RA02005K-s001.pdf]

Supplementary Information (SI) for RSC Advances.
This journal is © The Royal Society of Chemistry 2025

Supplementary materials

I- NMR data of the isolated compounds:

Compound 2

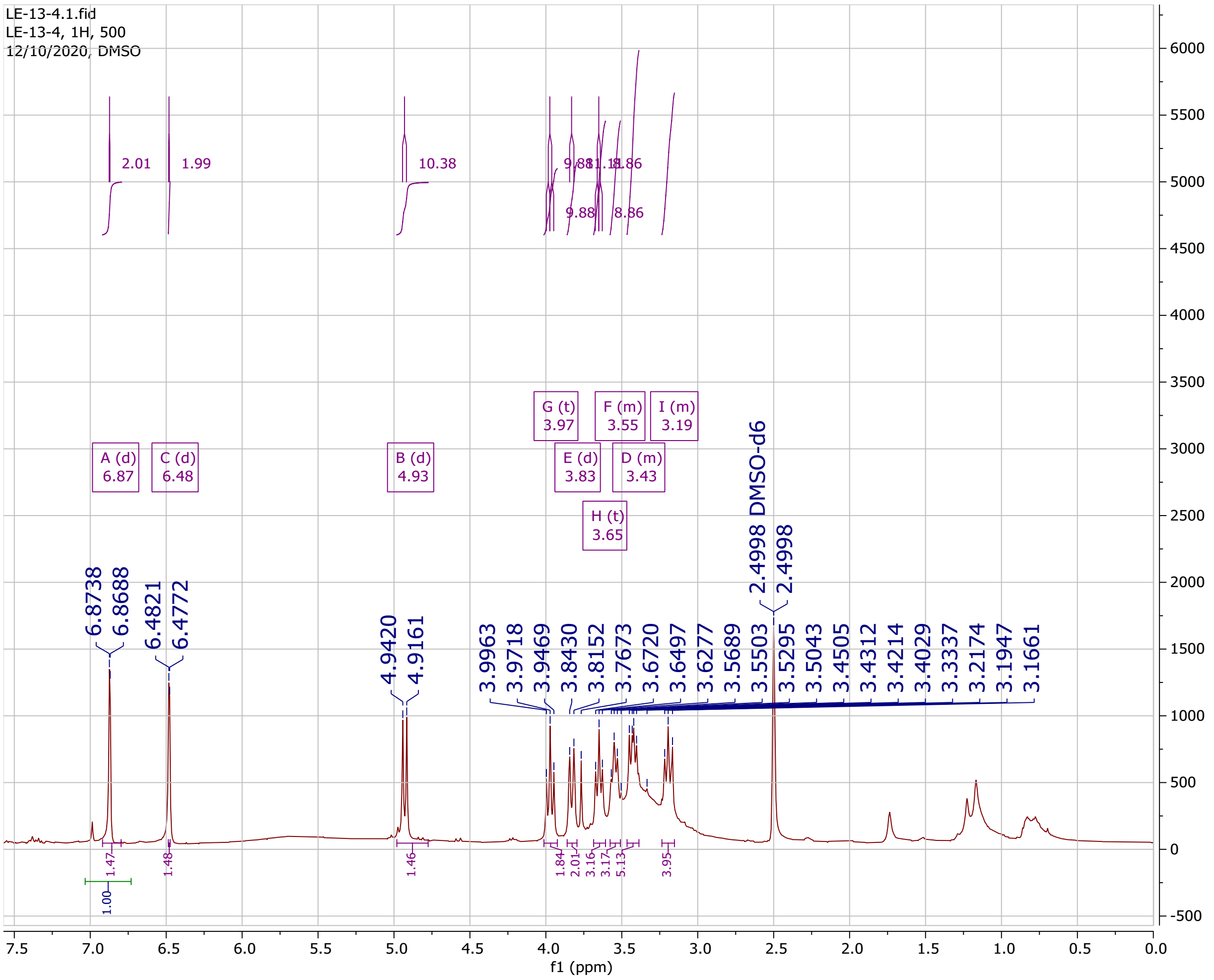

Fig. 1 ^{1}H NMR spectrum of compound 2

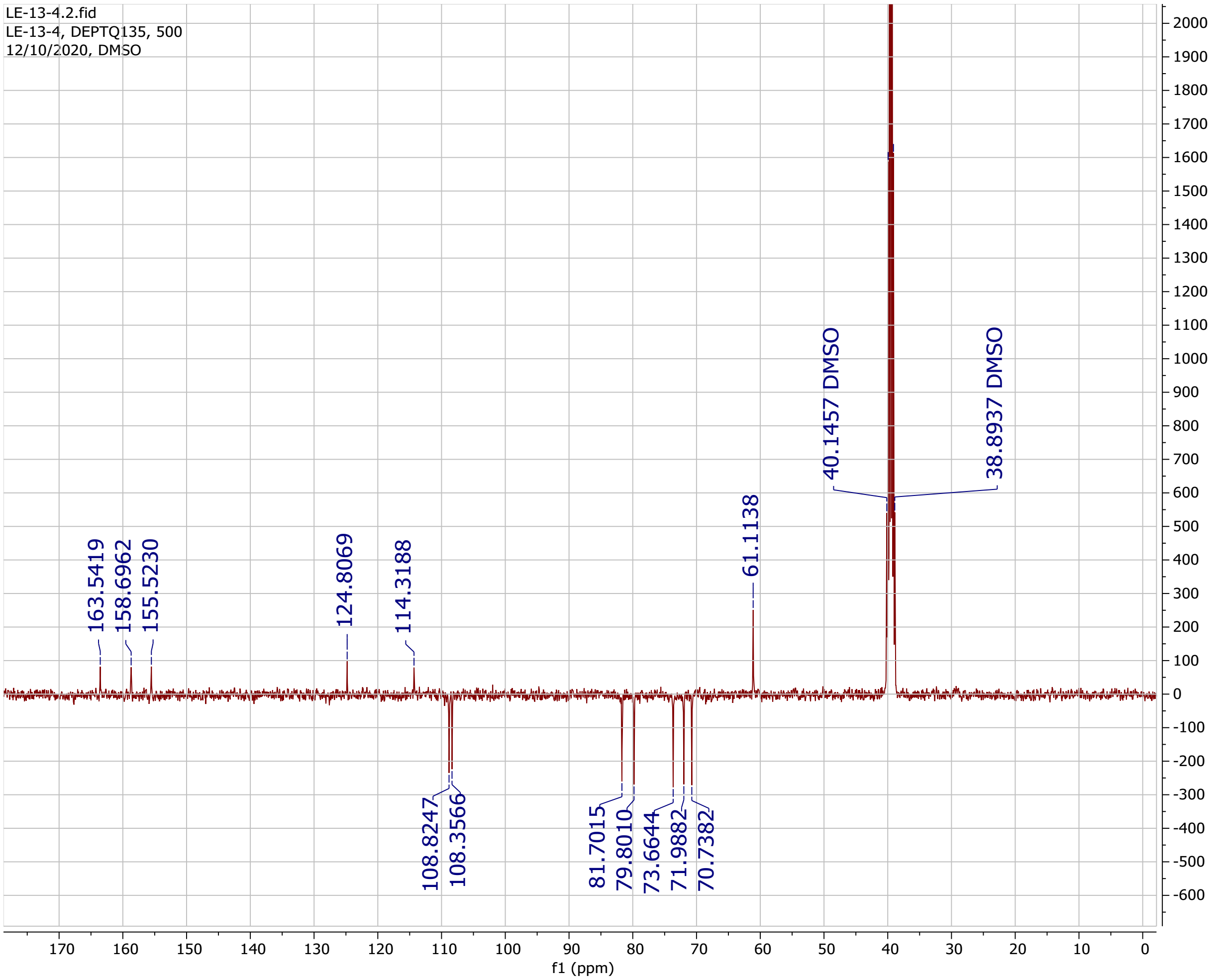

Fig. 2 DEPT Q-135 NMR spectrum of compound 2

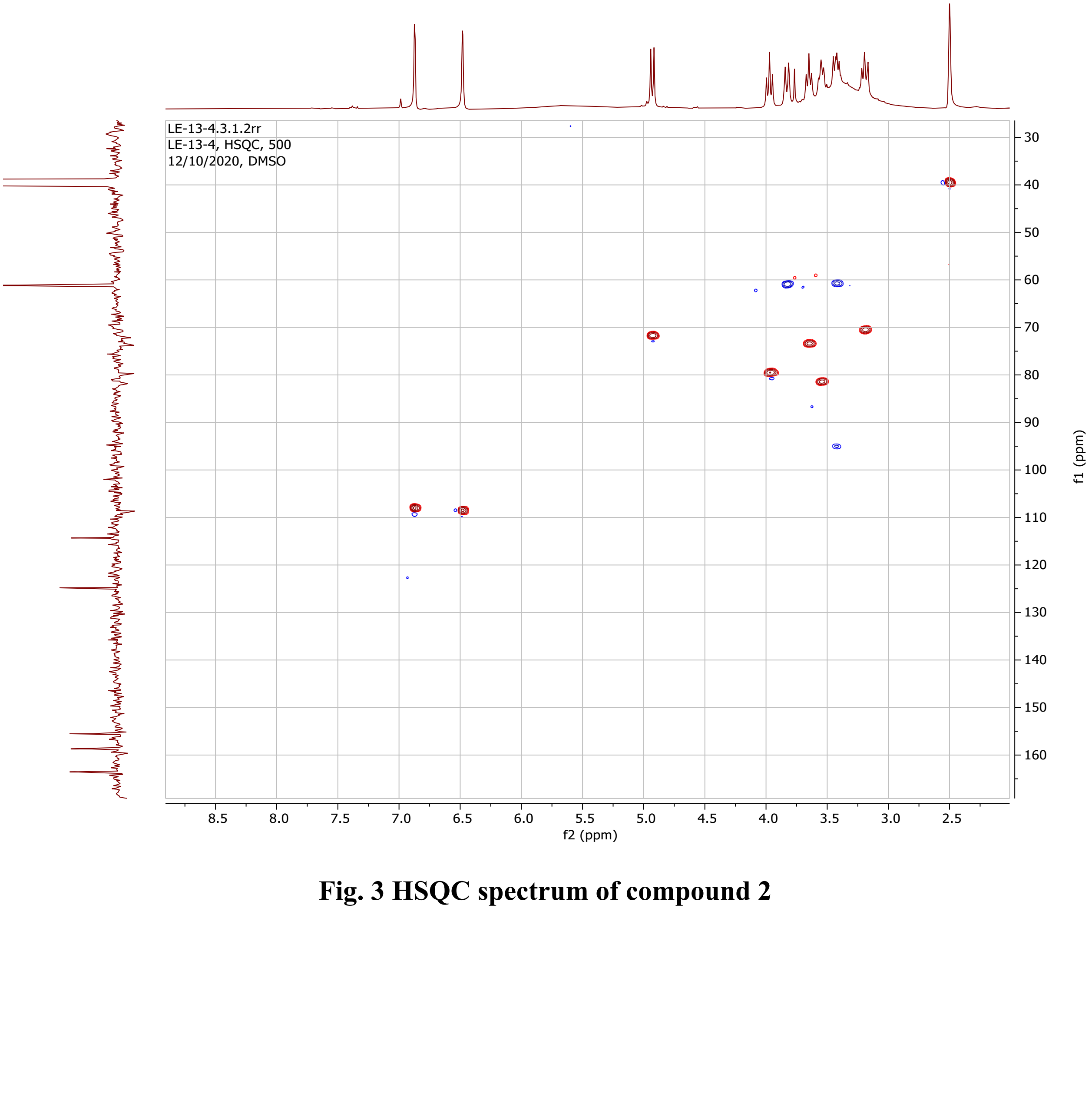

Fig. 3 HSQC spectrum of compound 2

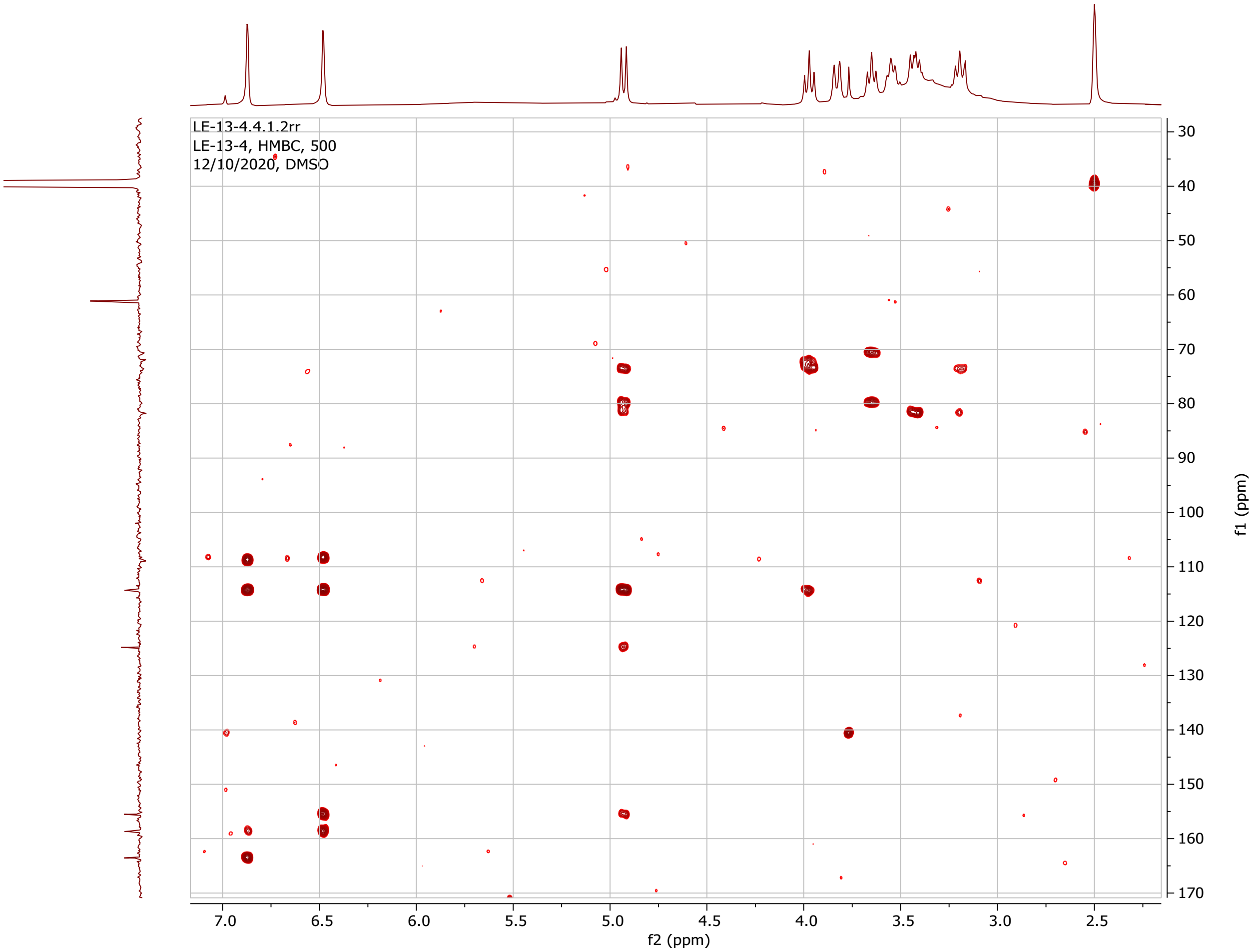

Fig. 4 HMBC spectrum of compound 2

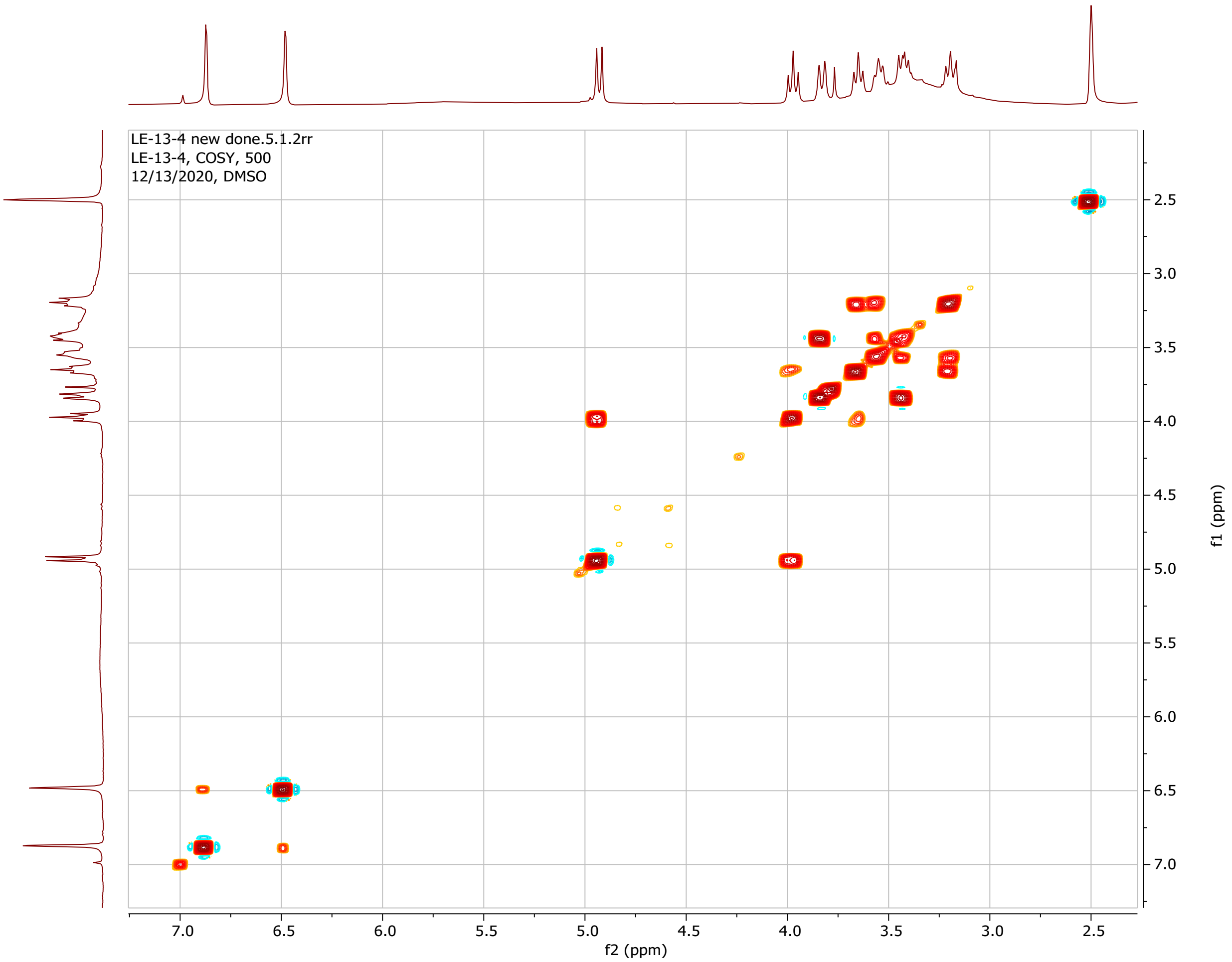

Fig. 5 ^{1}H ^{1}H COSY spectrum of compound 2

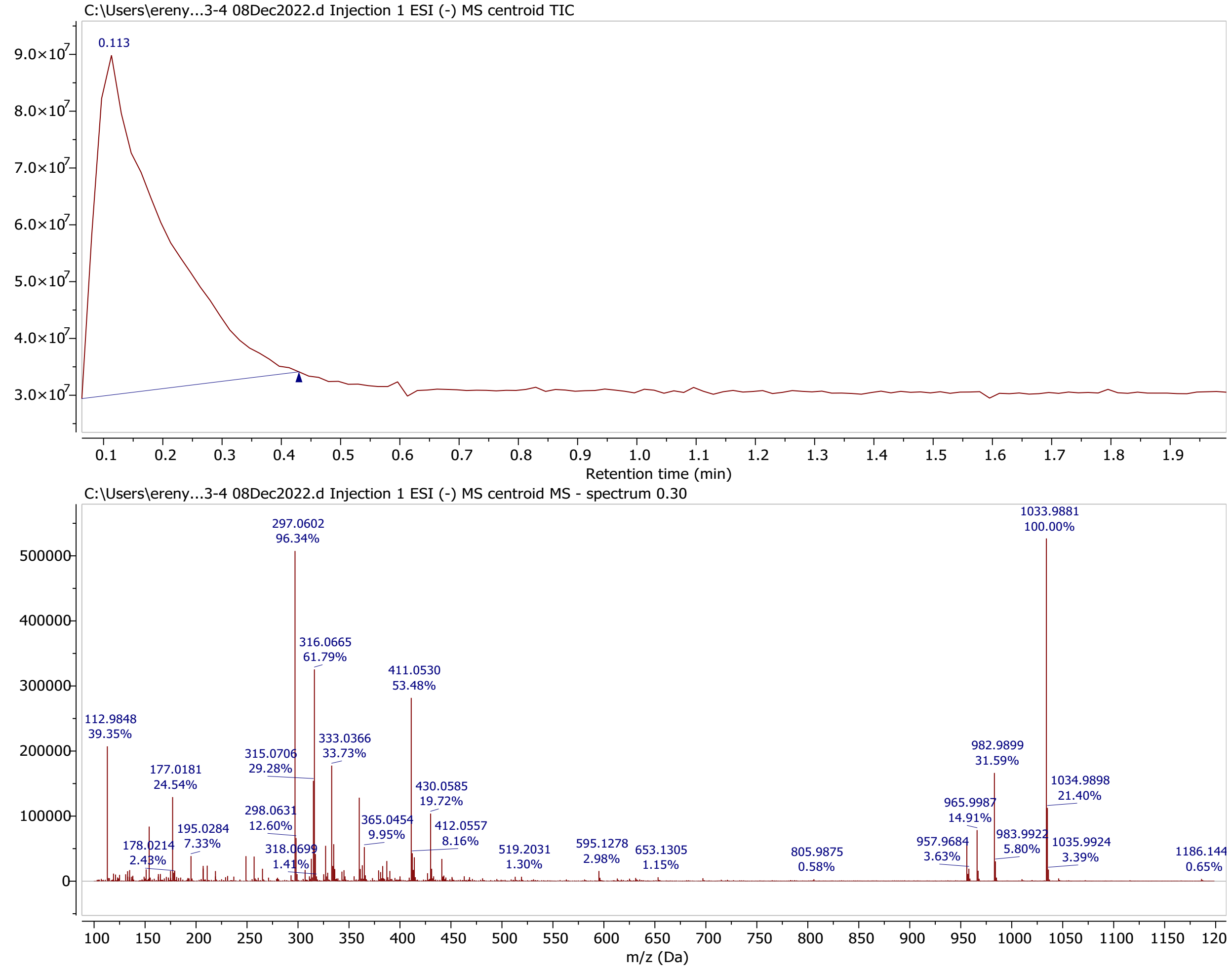

Fig. 6 HRESIMS spectrum of compound 2 in negative mode

Compound 5

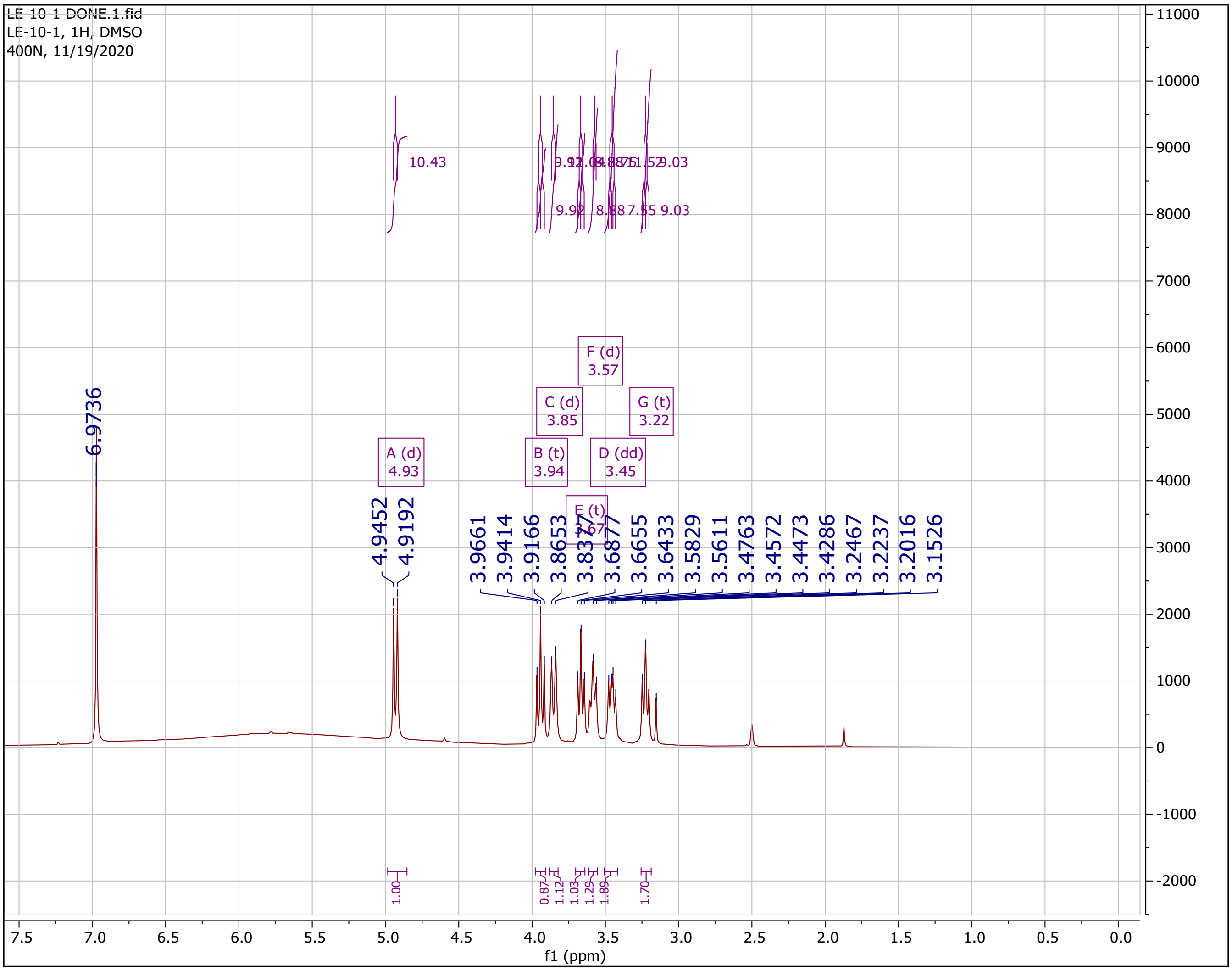

Fig. 7 ^{1}H NMR spectrum of compound 5

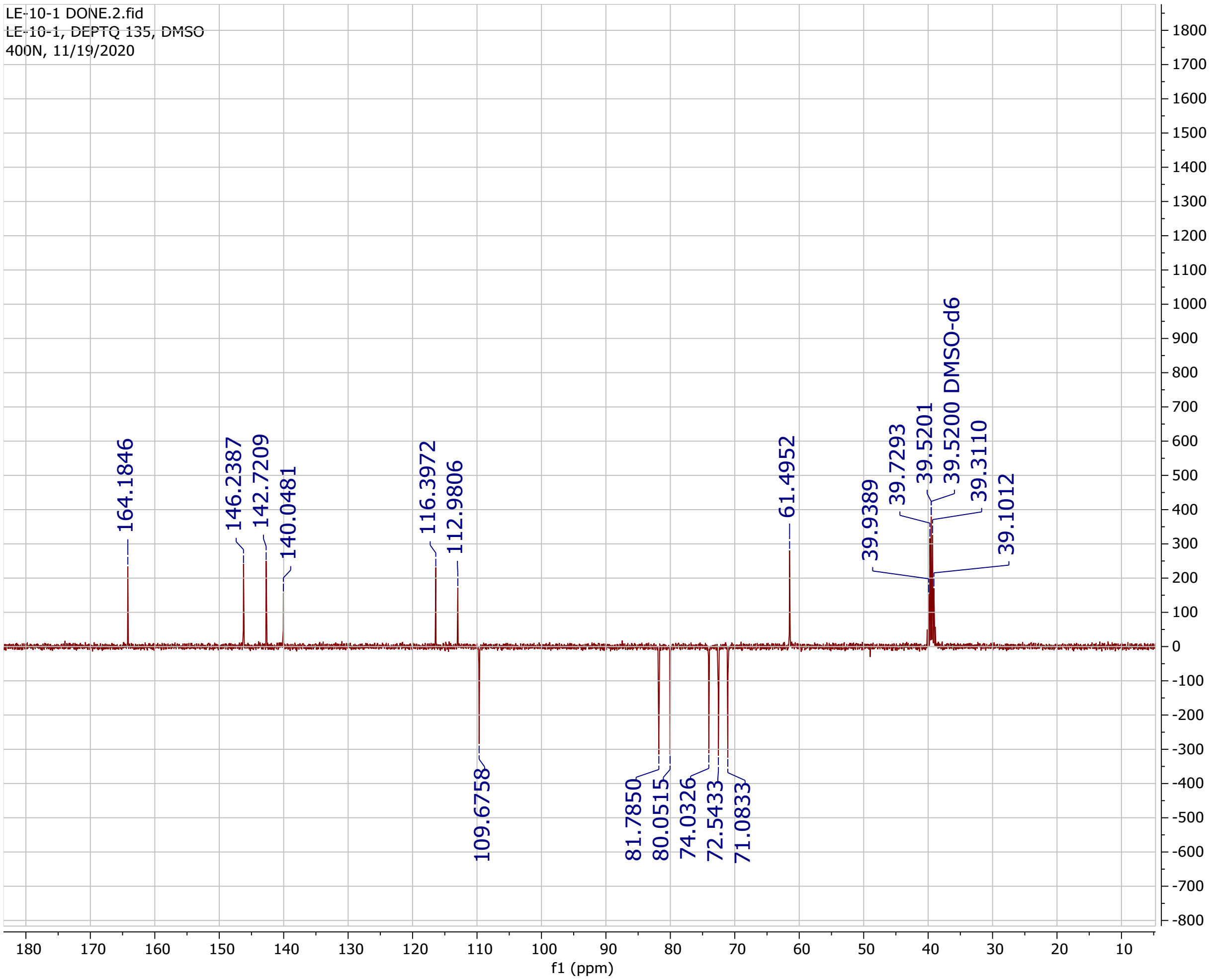

Fig. 8 DEPT Q-135 NMR spectrum of compound 5

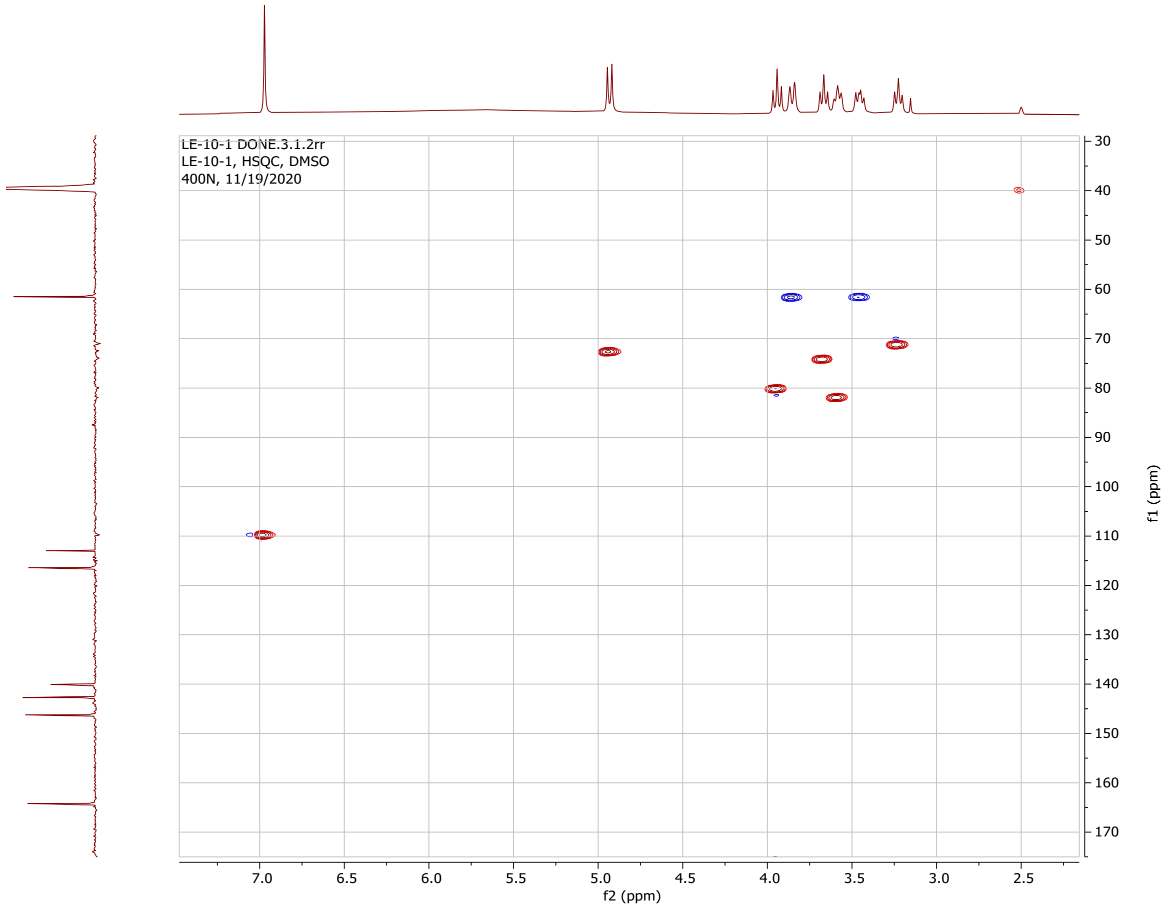

Fig. 9 HSQC spectrum of compound 5

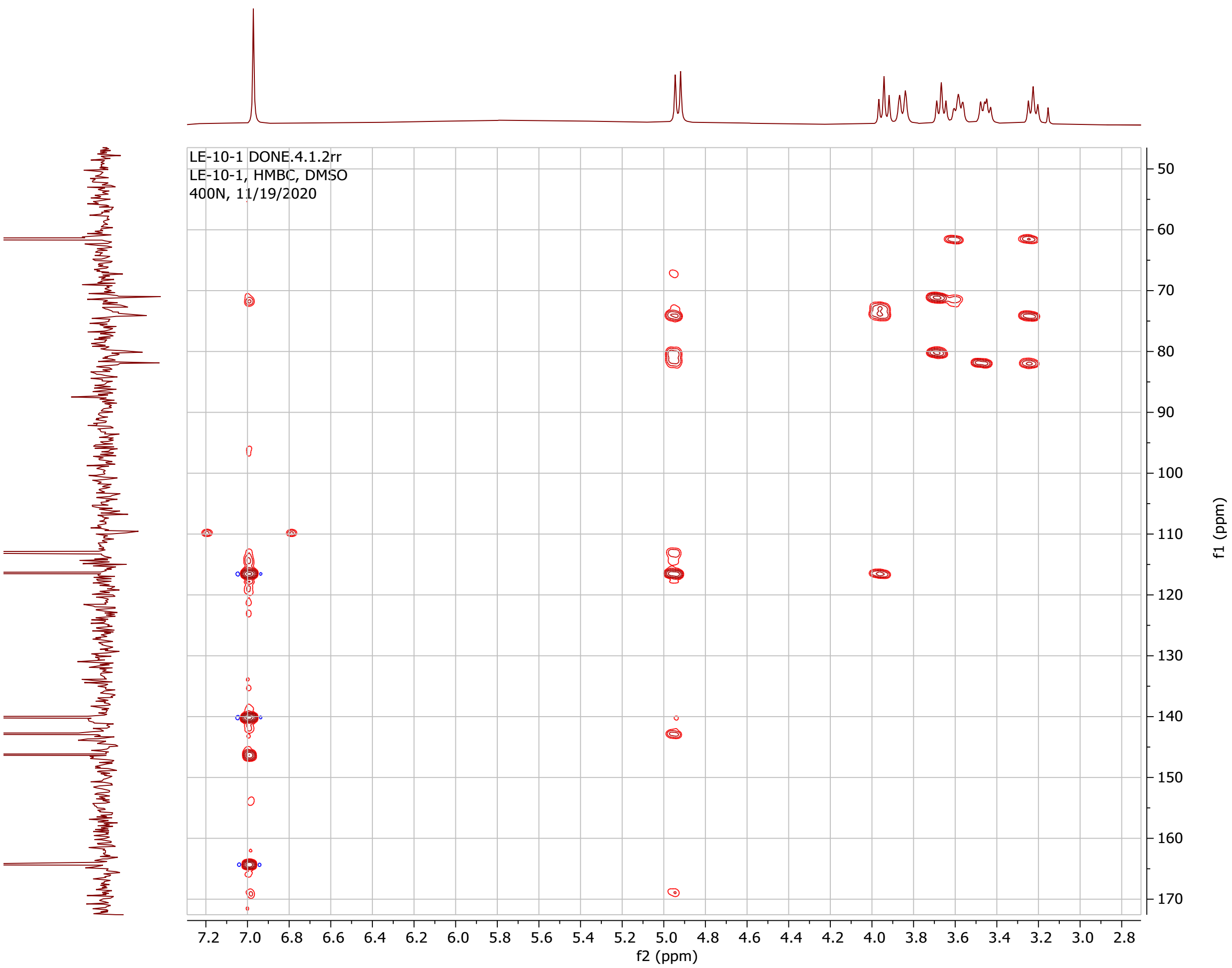

Fig. 10 HMBC spectrum of compound 5

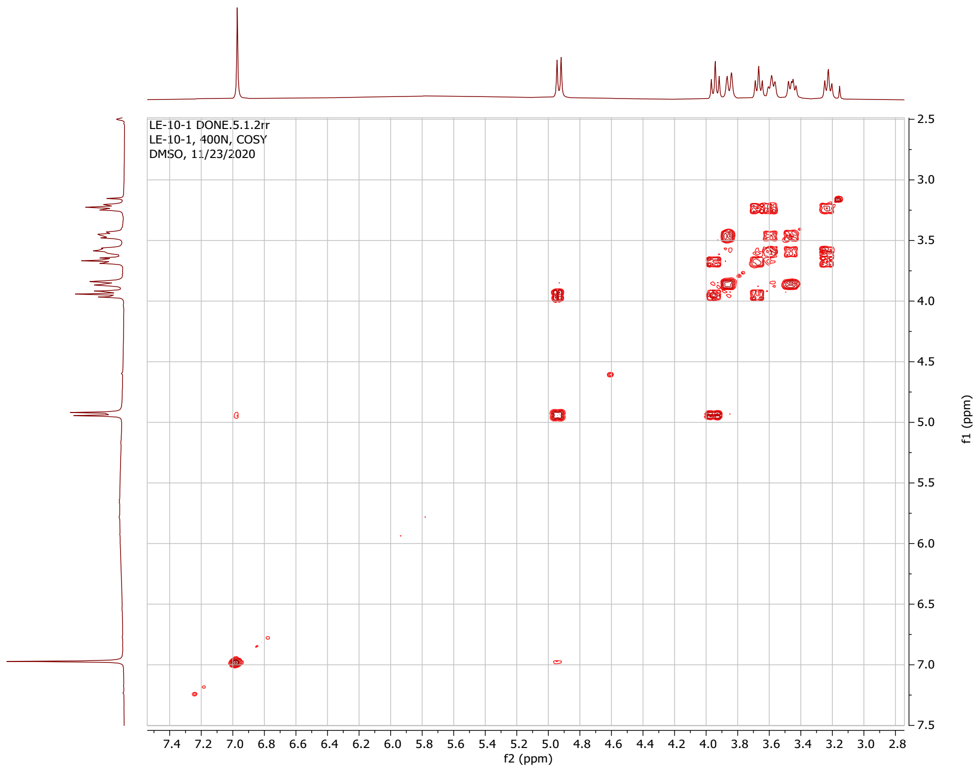

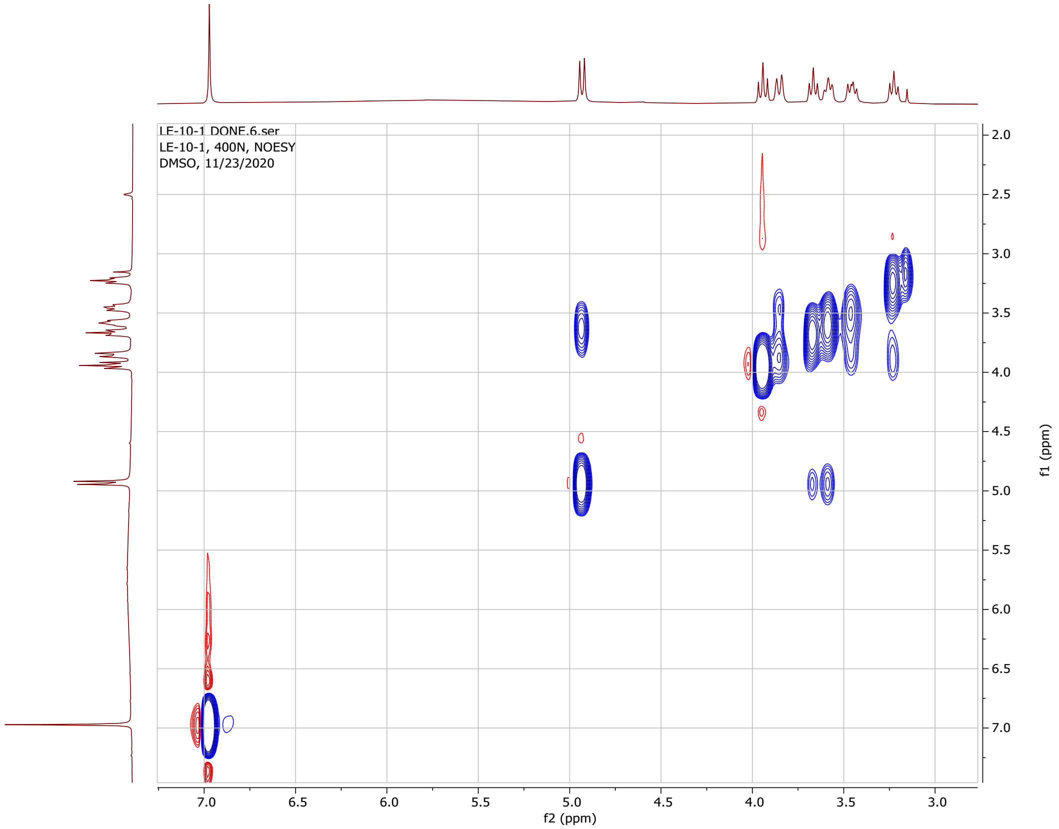

Fig. 11 ^{1}H ^{1}H COSY and ^{1}H ^{1}H NOESY spectra of compound 5

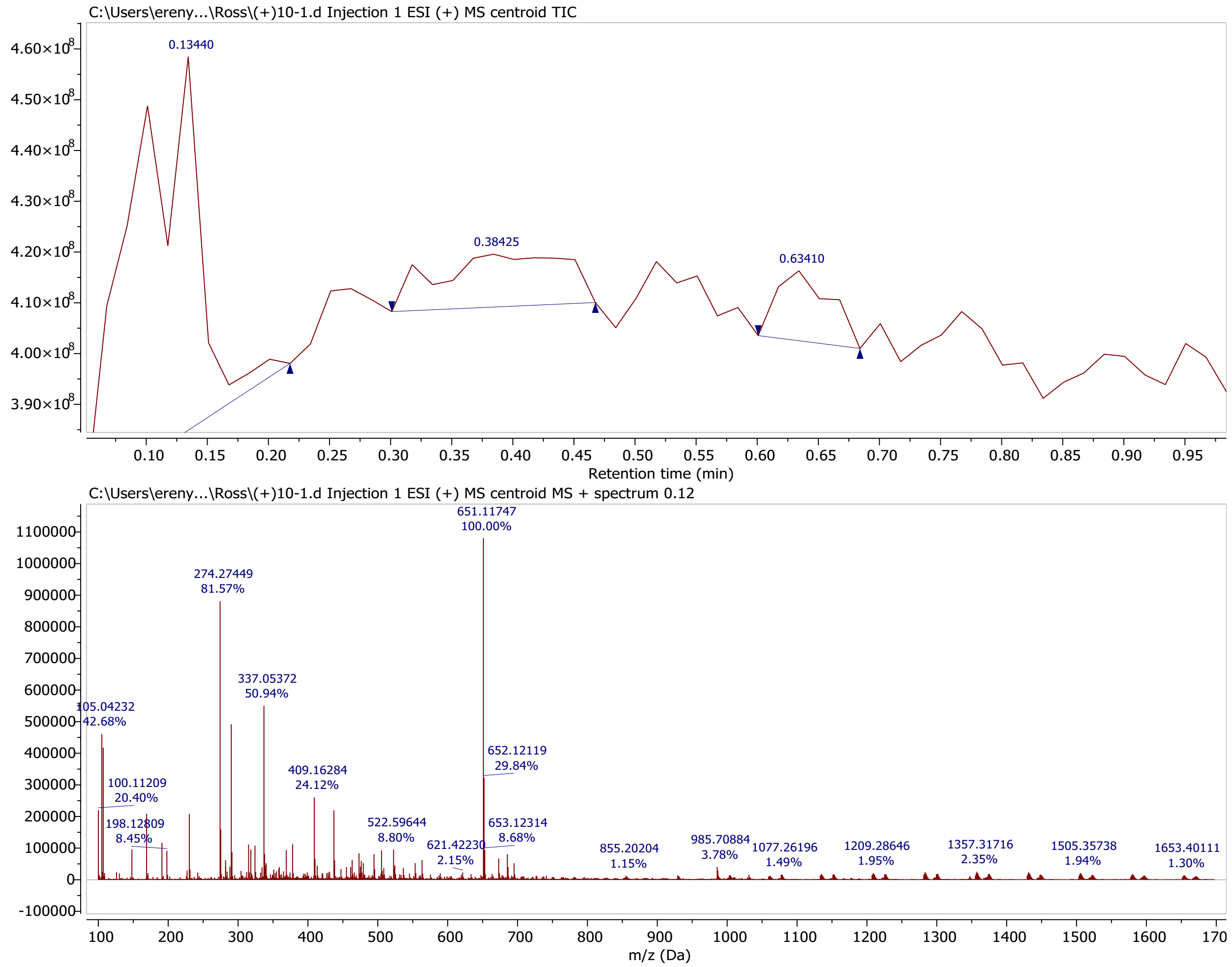

Fig. 12 HRESIMS spectrum of compound 5 in positive mode

Compound 1

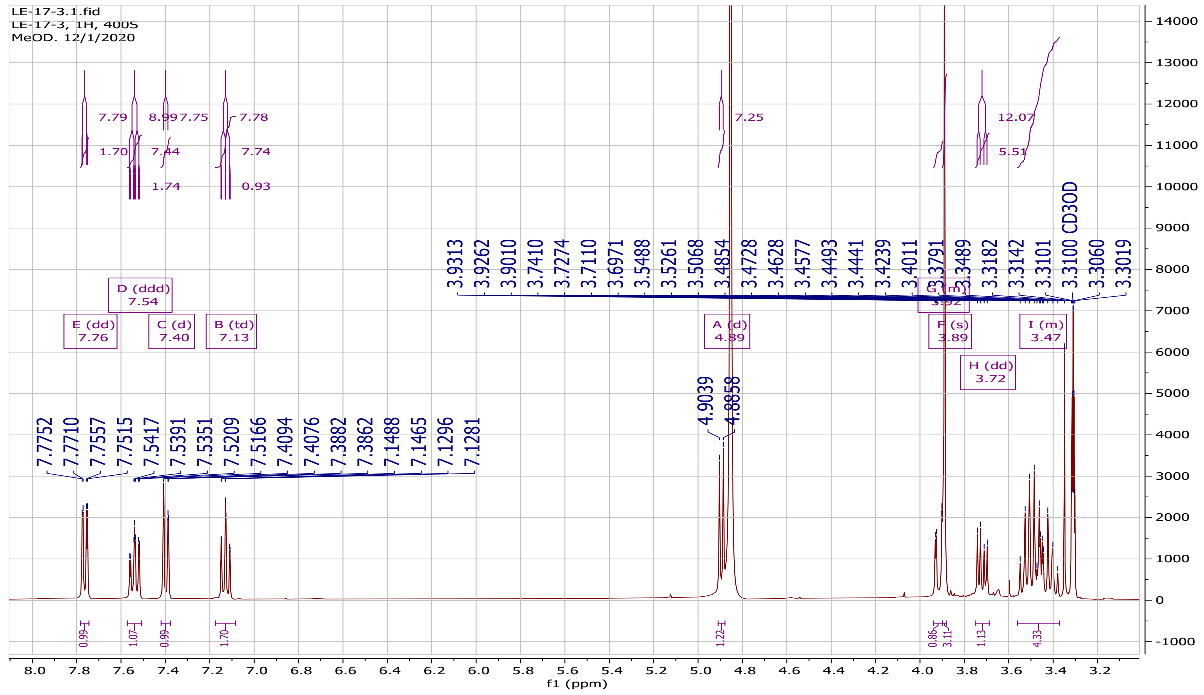

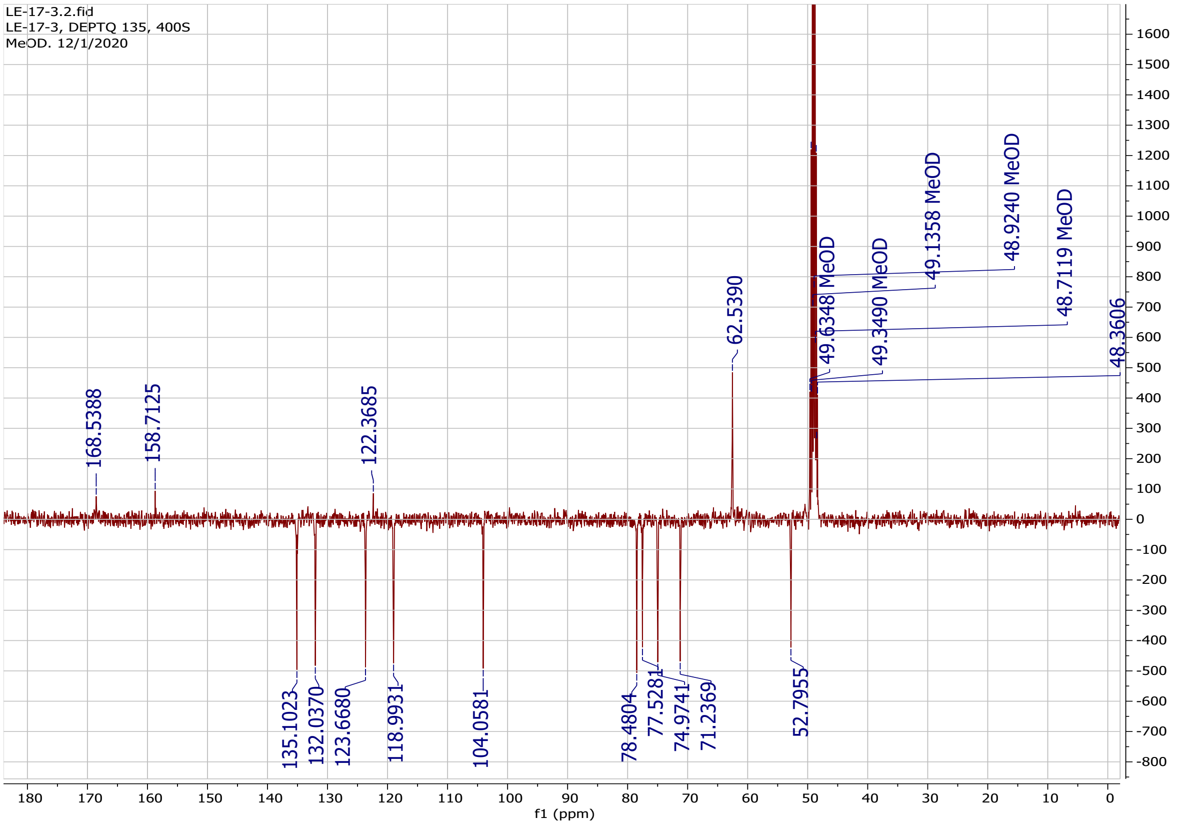

Fig. 13-a ^{1}H NMR and DEPT Q-135 spectra of compound 1

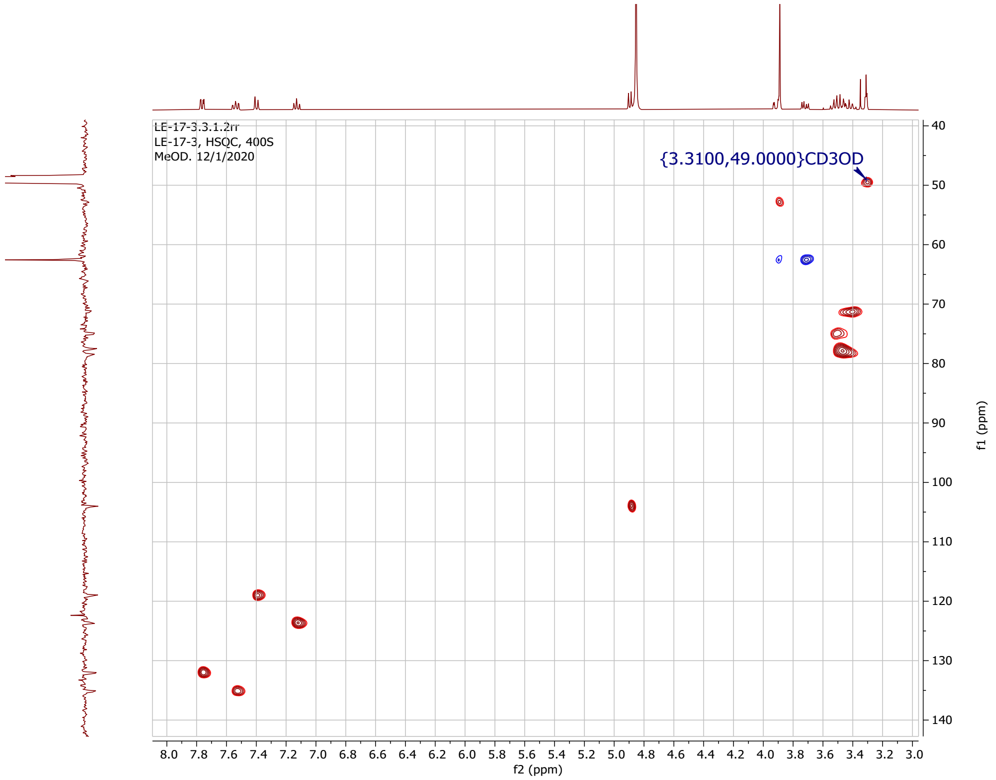

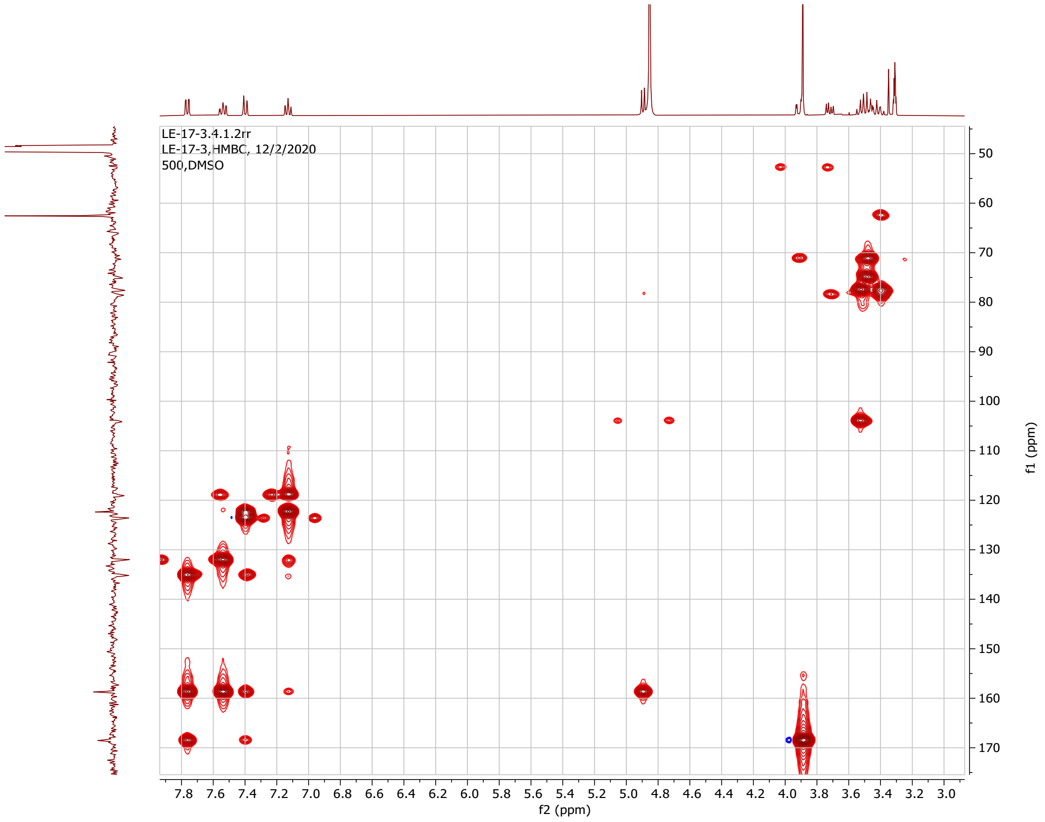

Fig. 13-b HSQC and HMBC spectra of compound 1

Compound 3

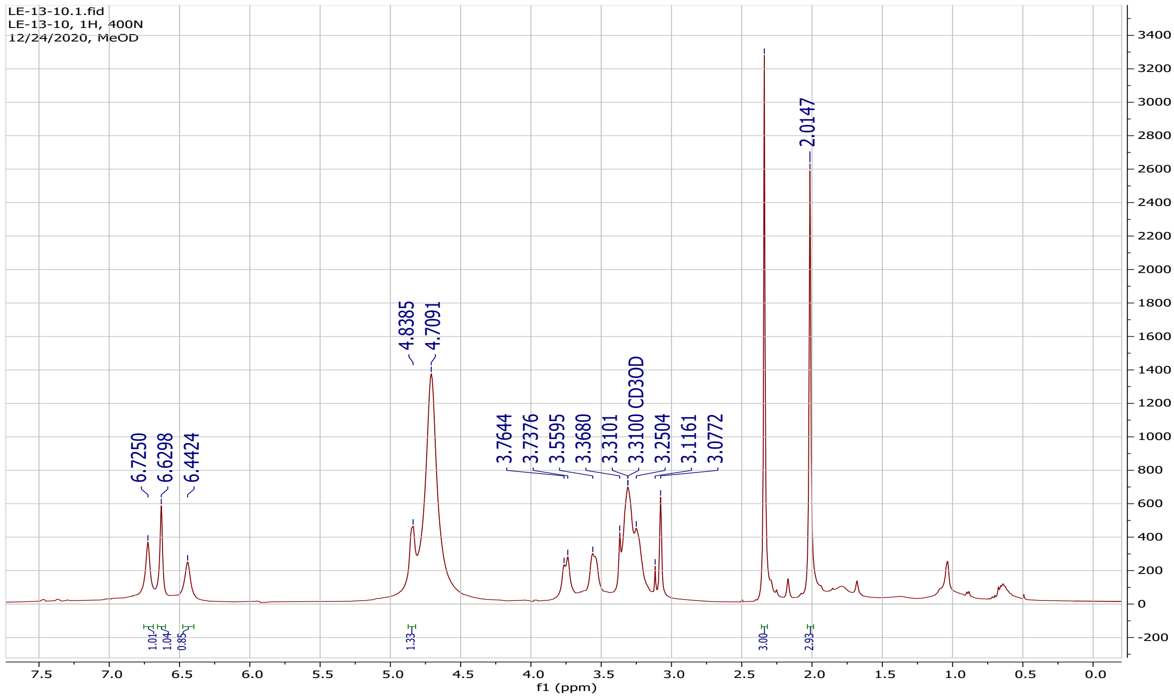

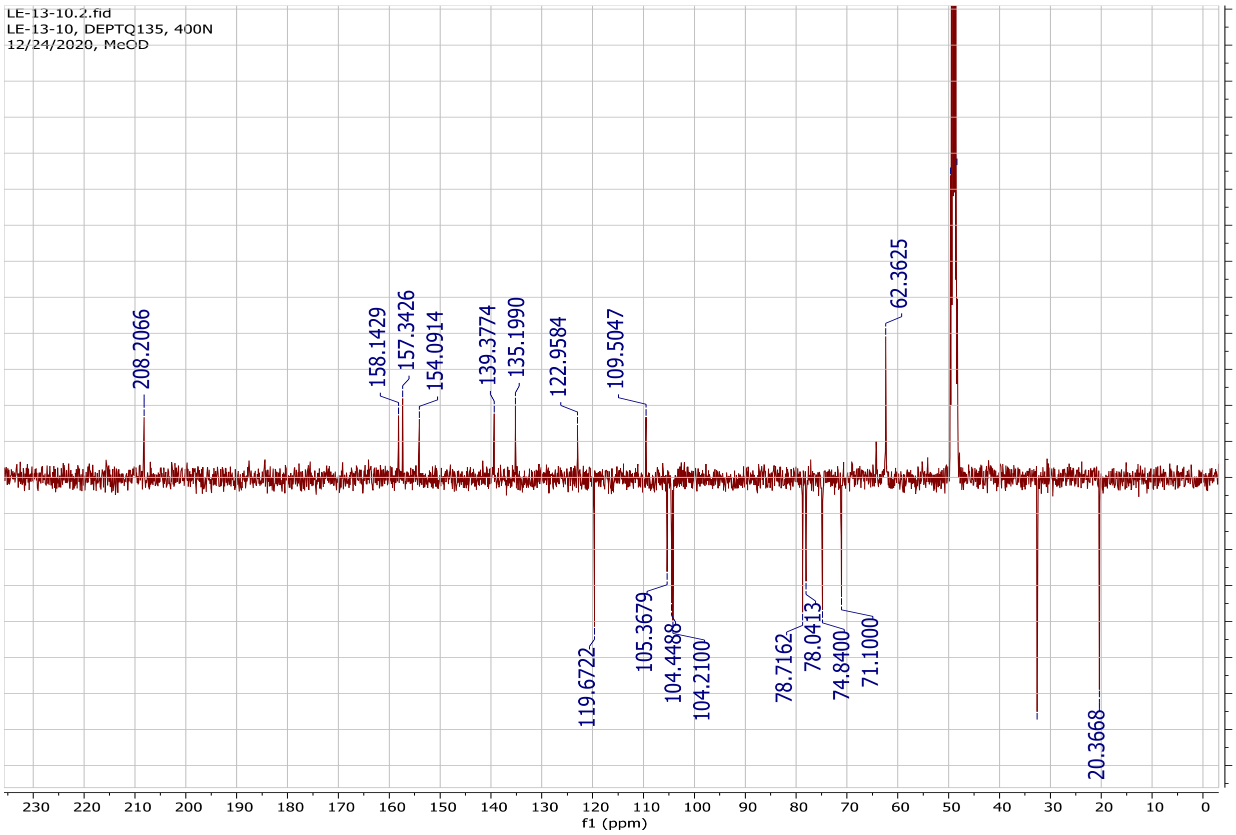

Fig. 14-a ^{1}H NMR and DEPT Q-135 spectra of compound 3

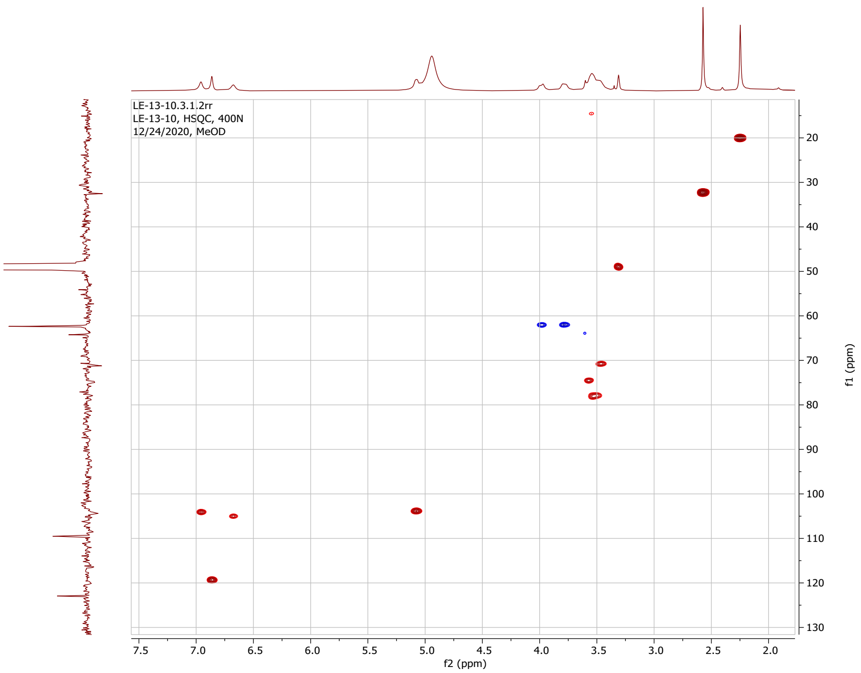

Fig. 14-b HSQC spectrum of compound 3

Compound 4

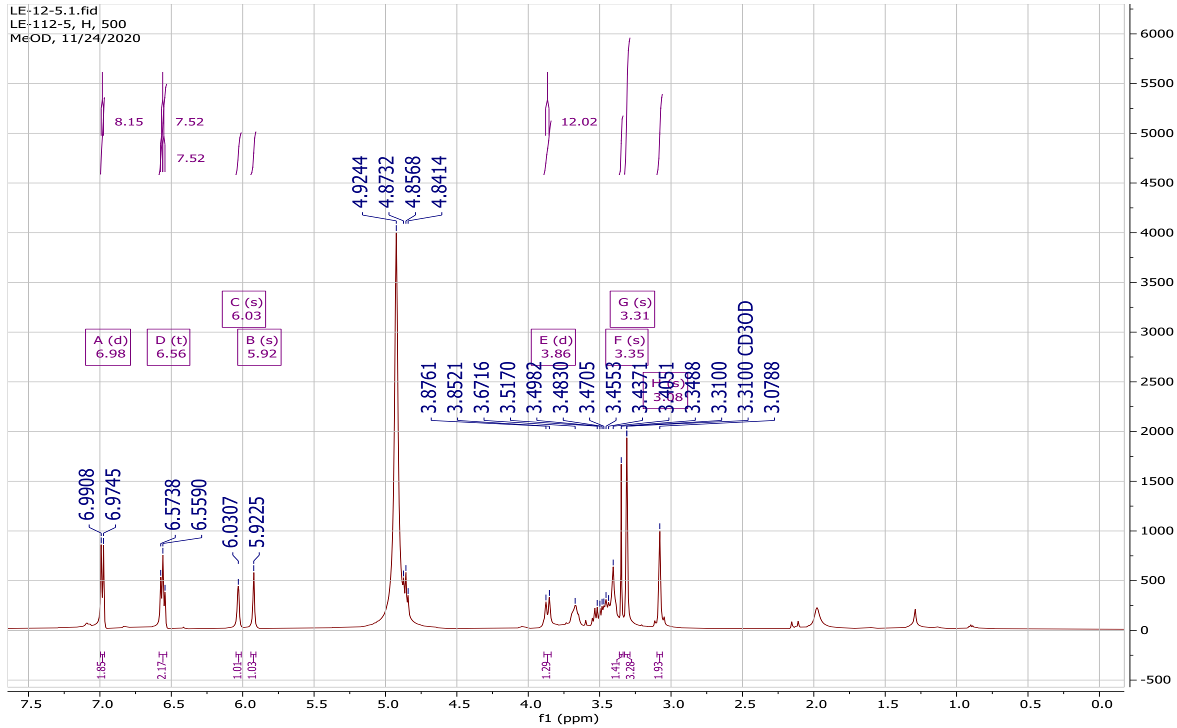

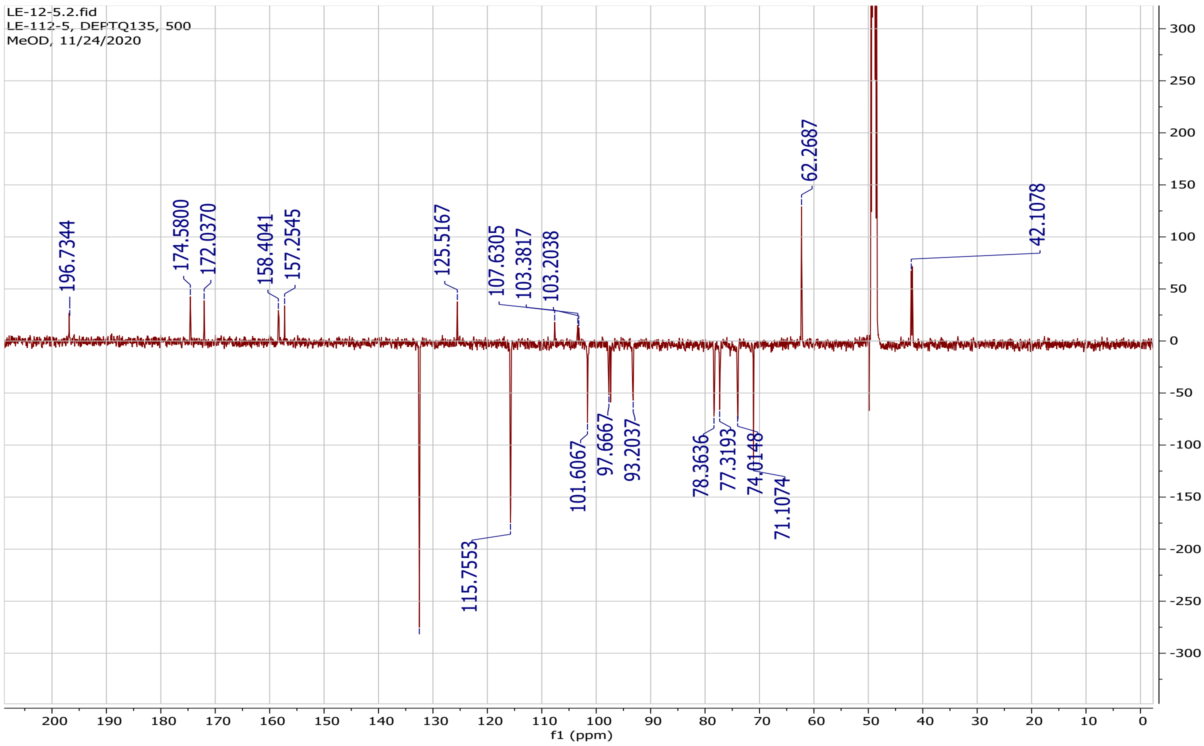

Fig. 15-a ^{1}H NMR and DEPT Q-135 spectra of compound 4

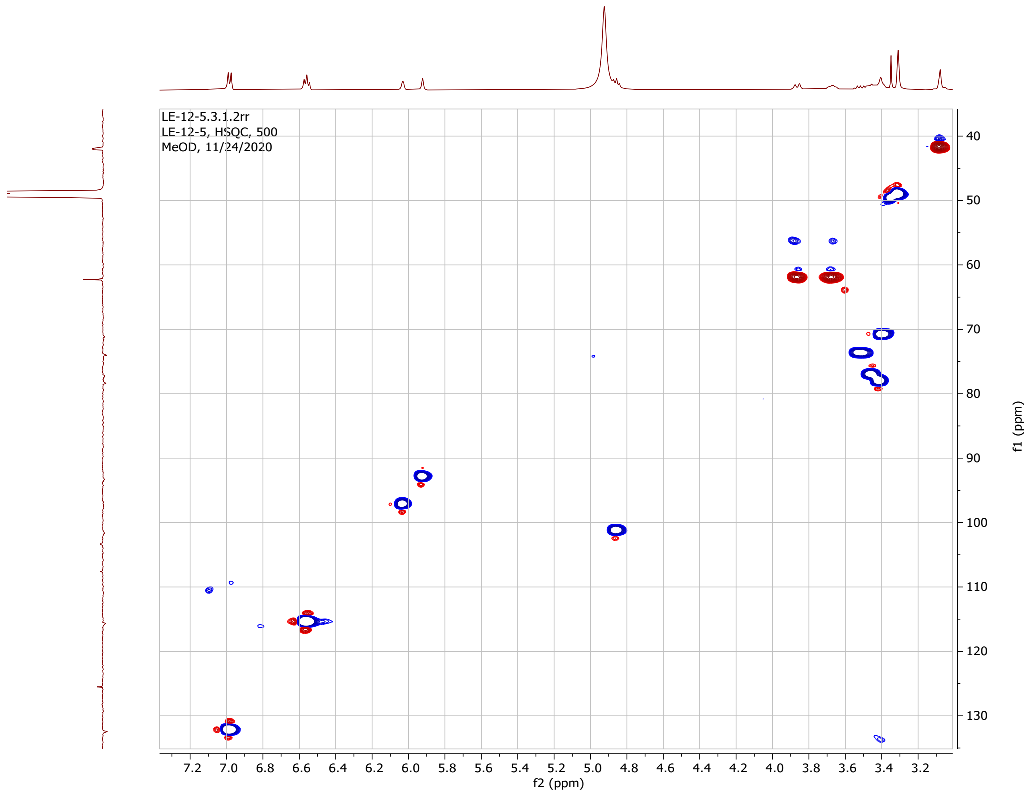

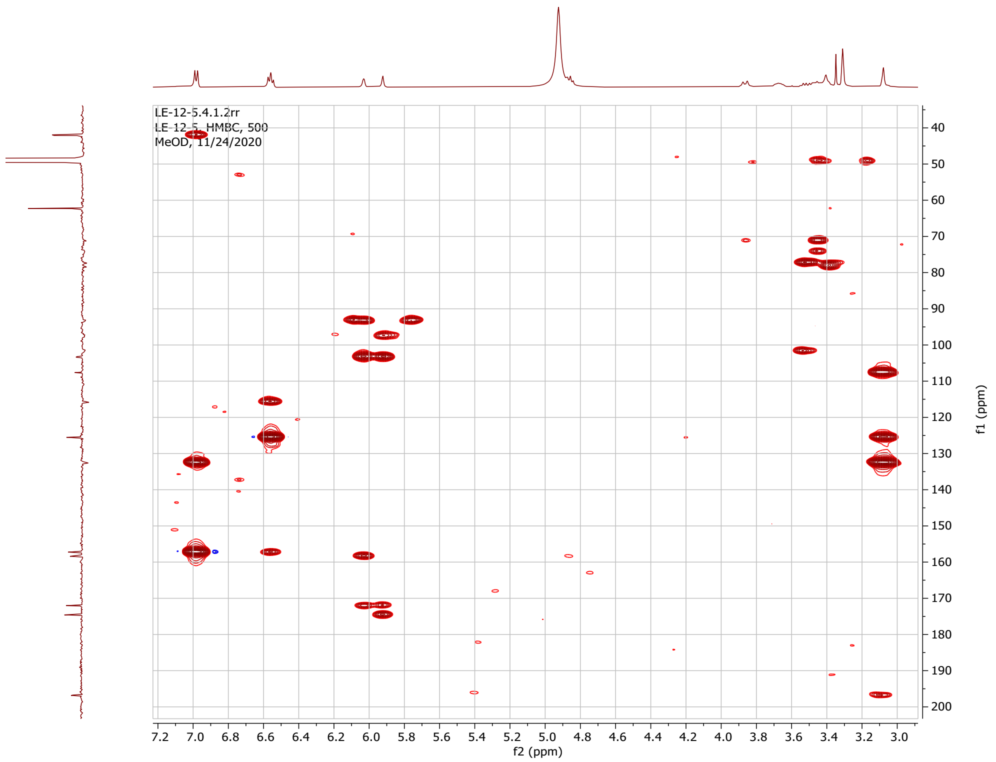

Fig. 15-b HSQC and HMBC spectra of compound 4

Compound 6

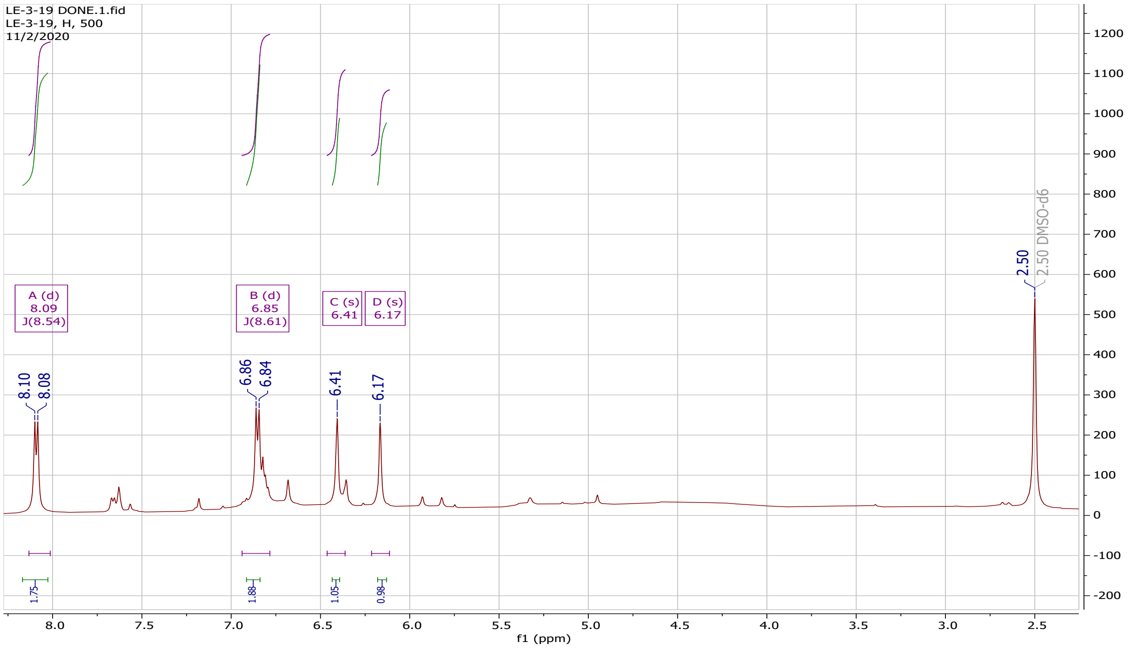

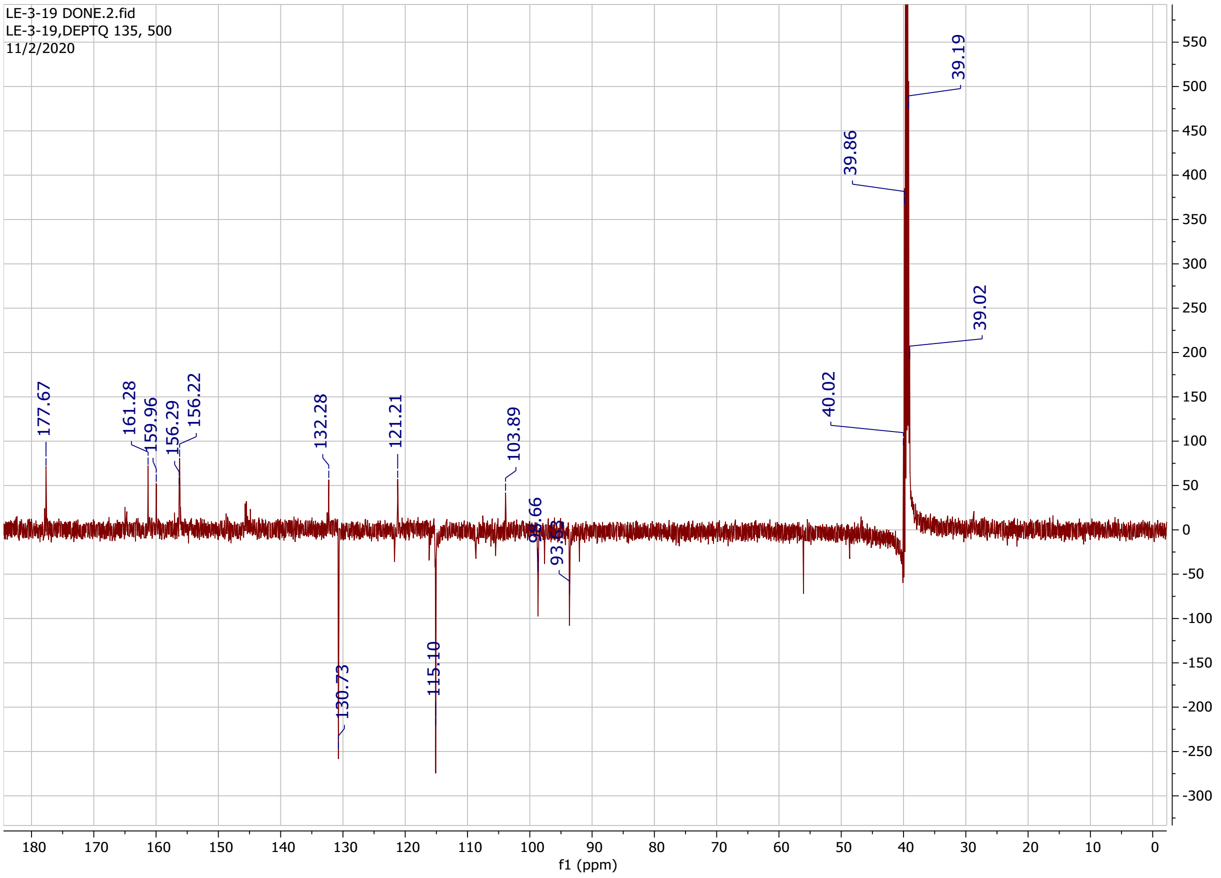

Fig. 16-a ^{1}H NMR and DEPT Q-135 spectra of compound 6

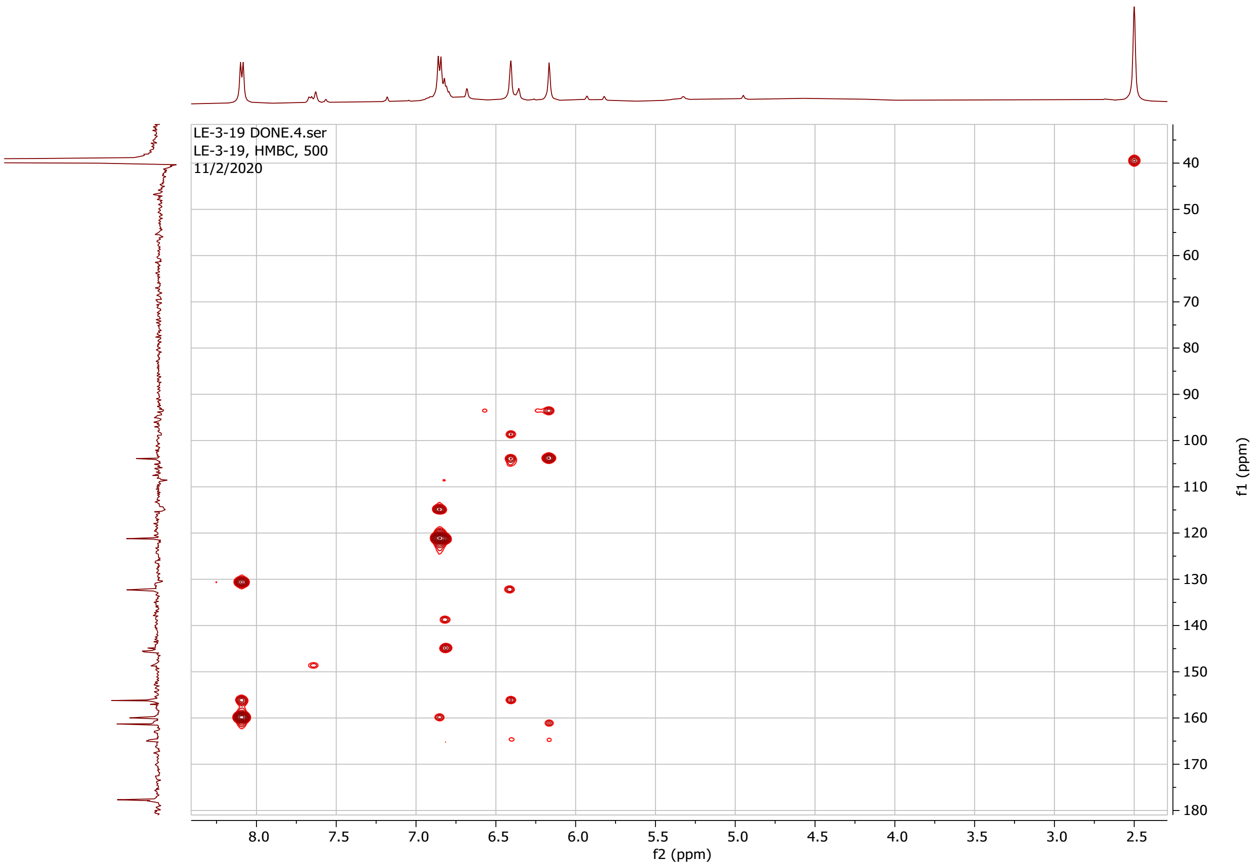

Fig. 16-b HMBC spectrum of compound 6

Compound 7

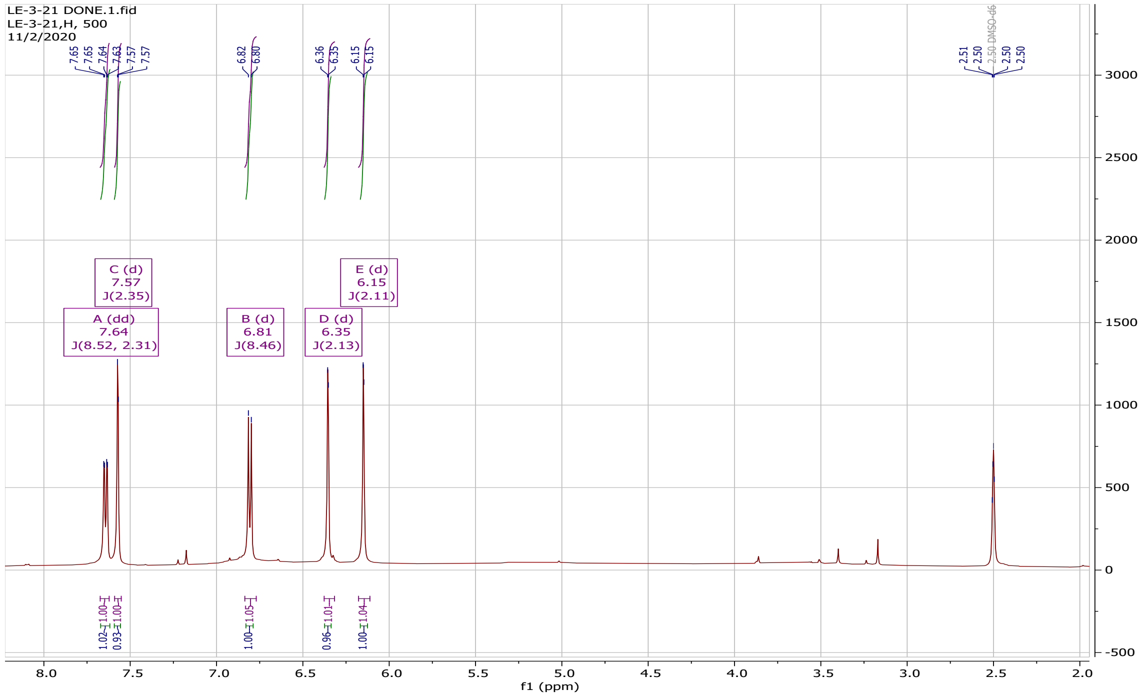

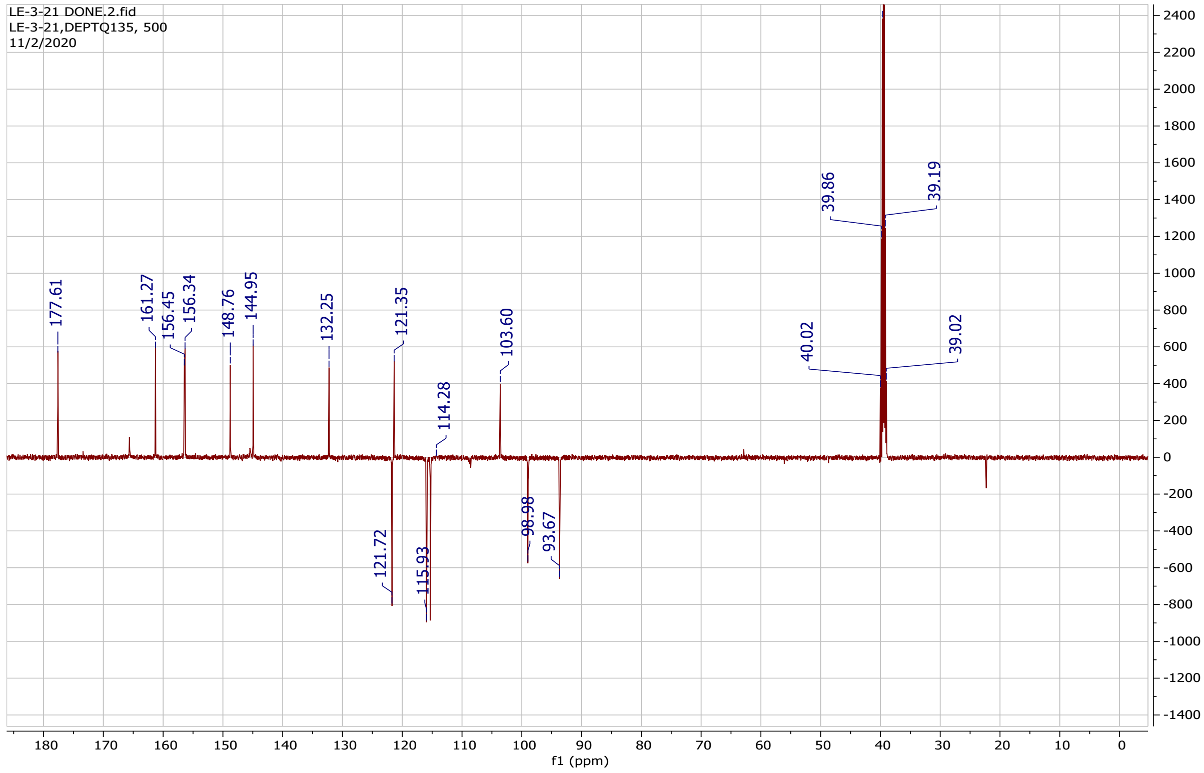

Fig. 17 ^{1}H NMR and DEPT Q-135 spectra of compound 7

Compound 8

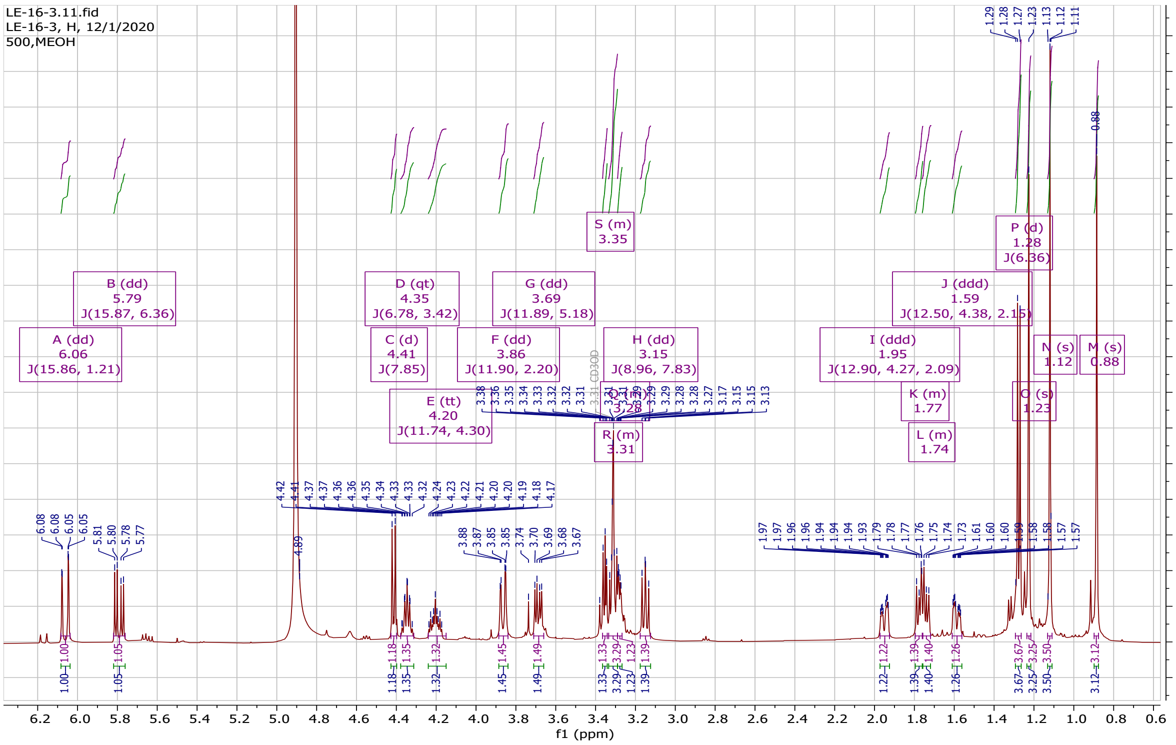

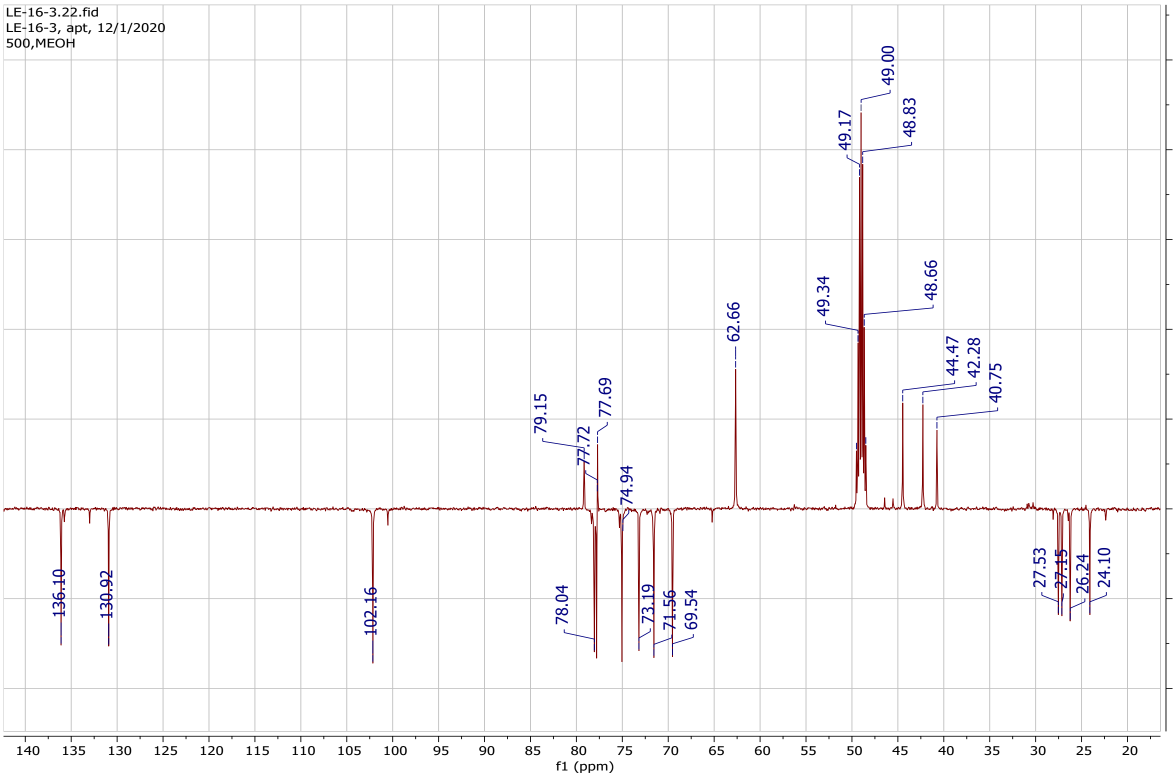

Fig. 18-a ^{1}H NMR and APT spectra of compound 8

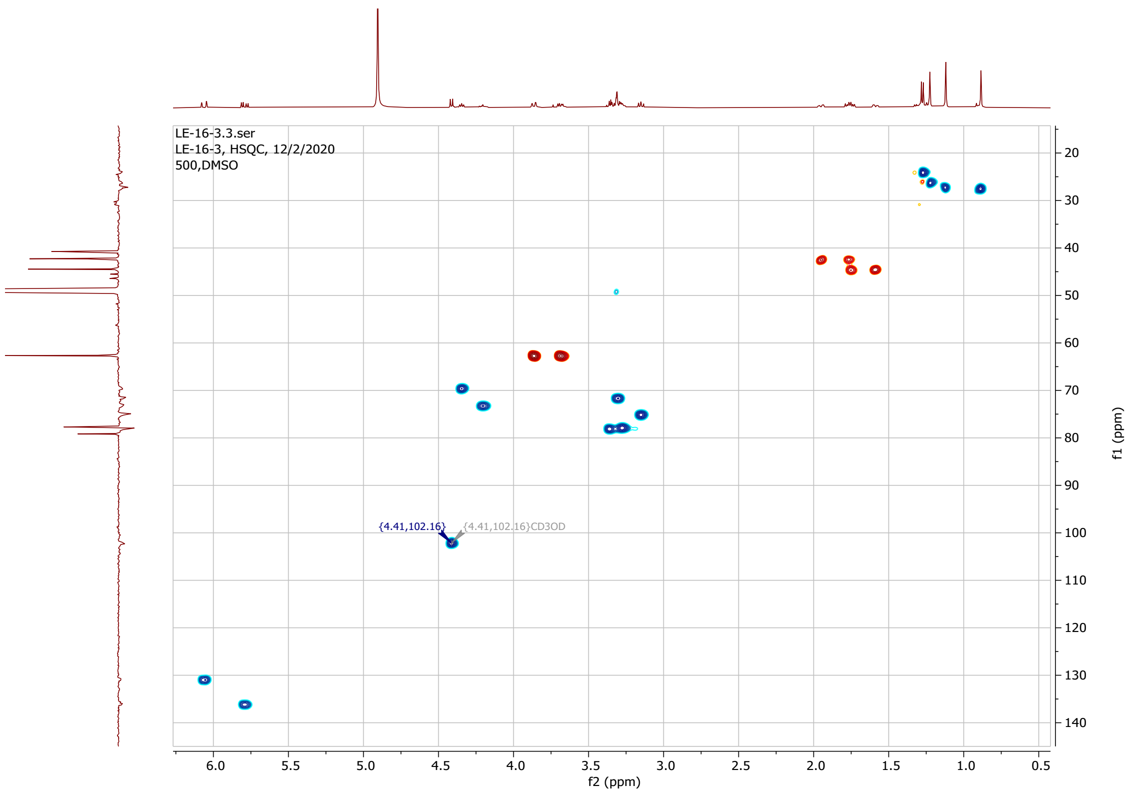

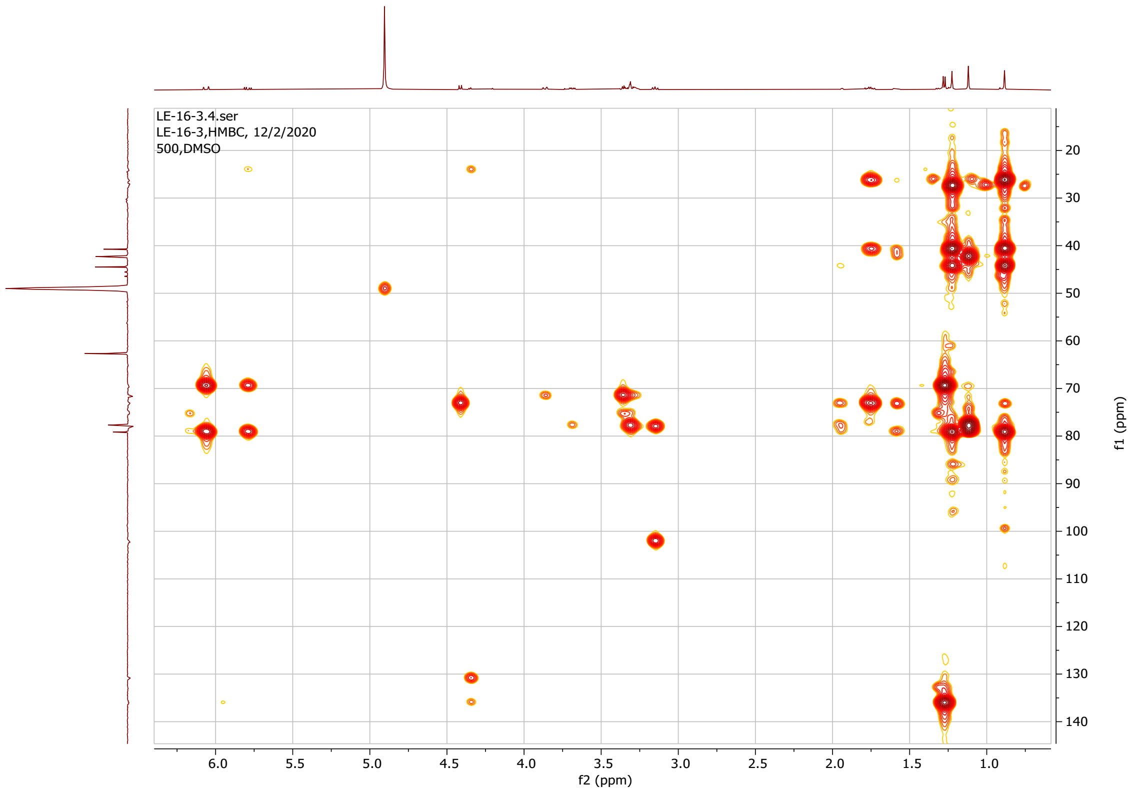

Fig. 18-b HSQC and HMBC spectra of compound 8

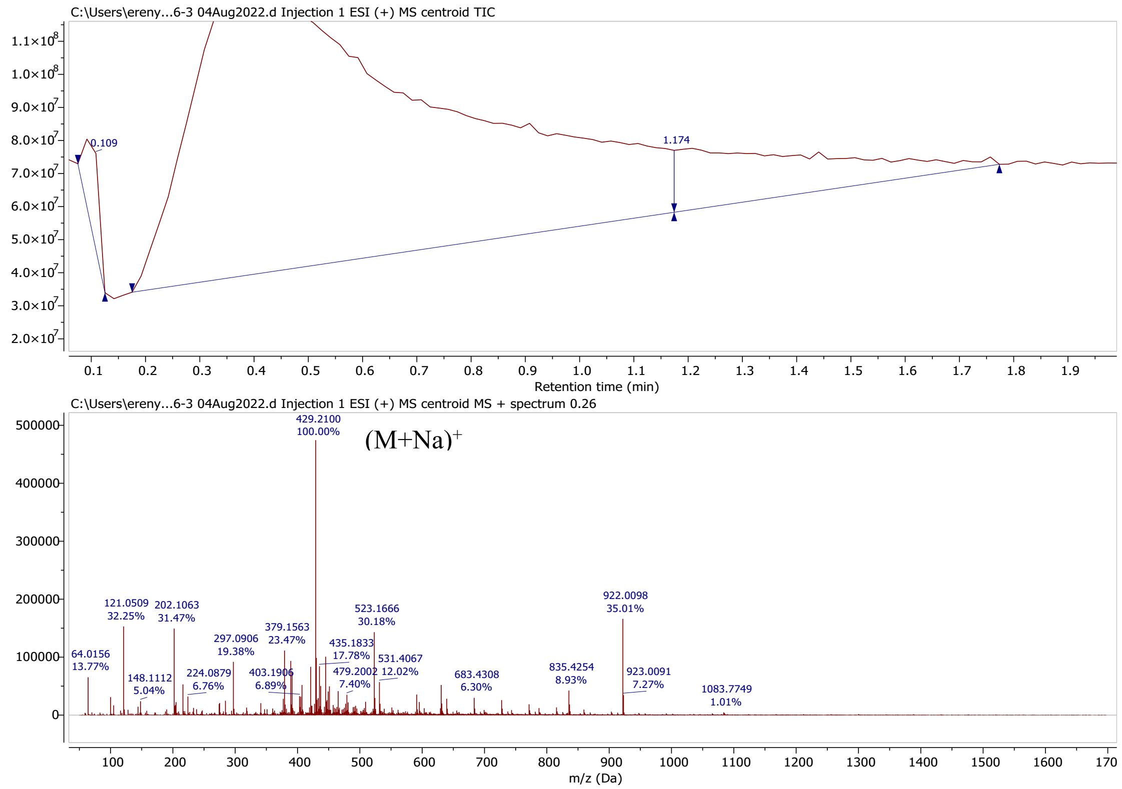

Fig. 18-c HRESIMS spectrum of compound 8 in positive mode

Compound 9

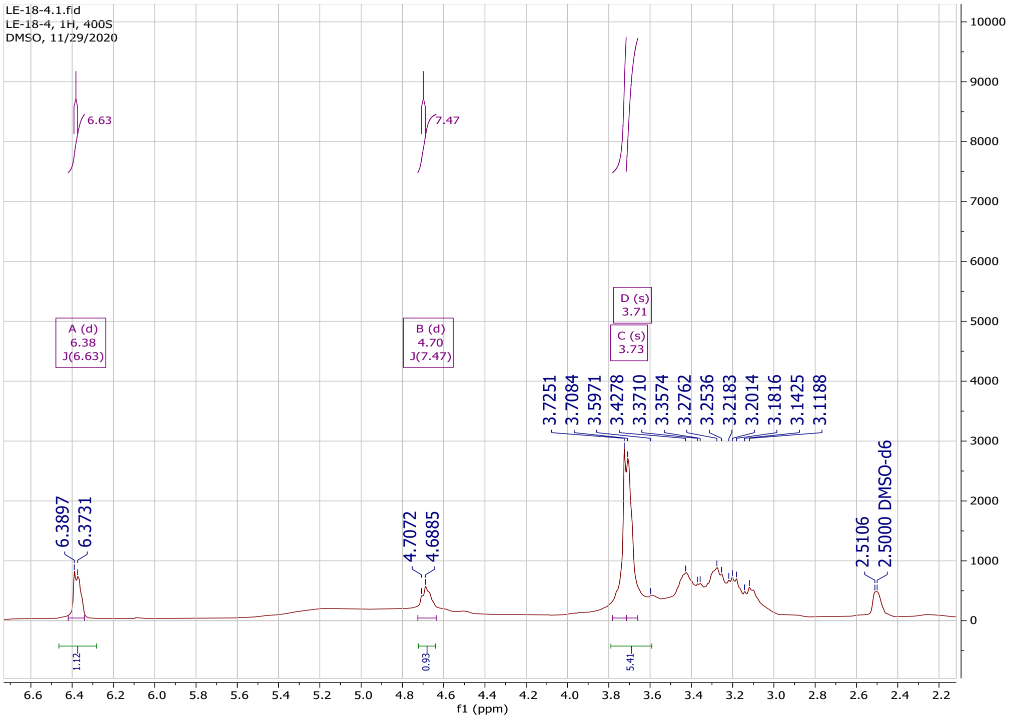

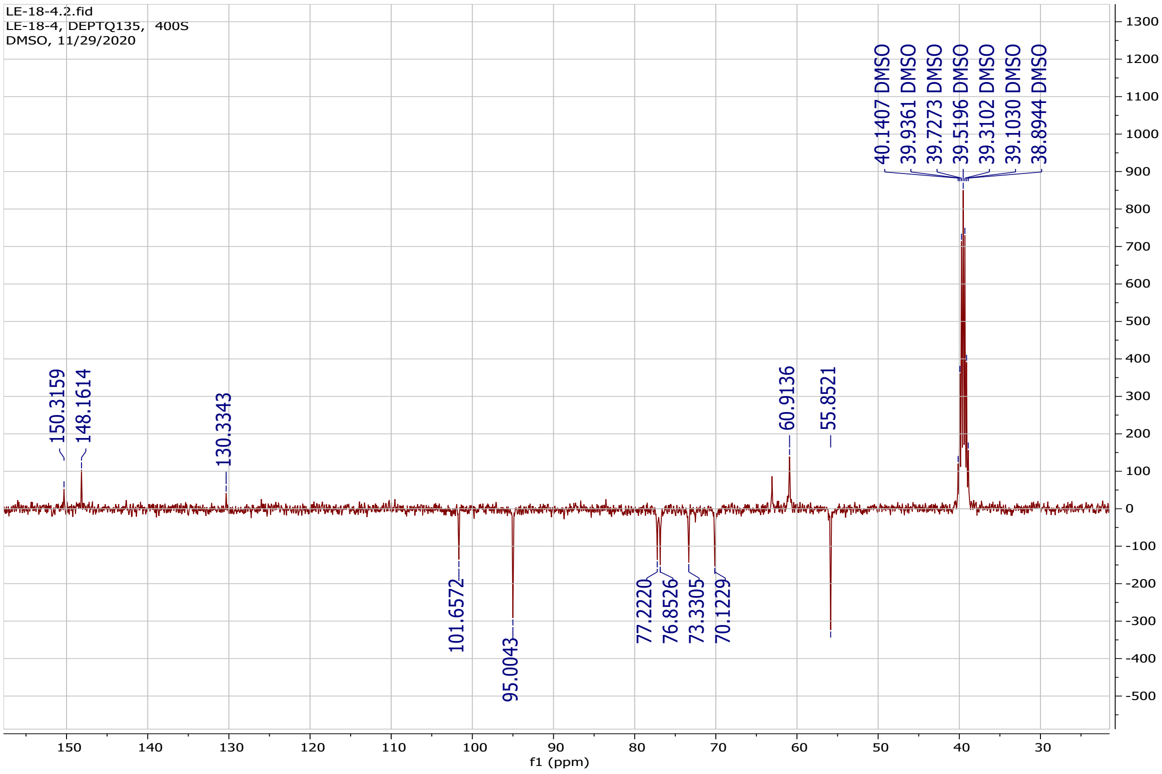

Fig. 19-a ^{1}H NMR and DEPT Q-135 spectra of compound 9

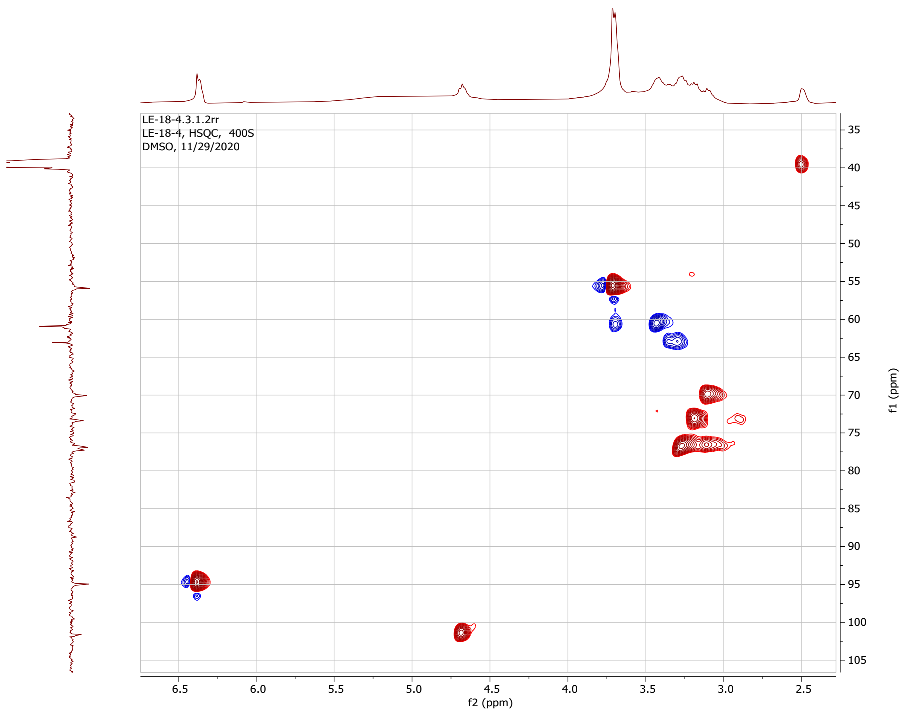

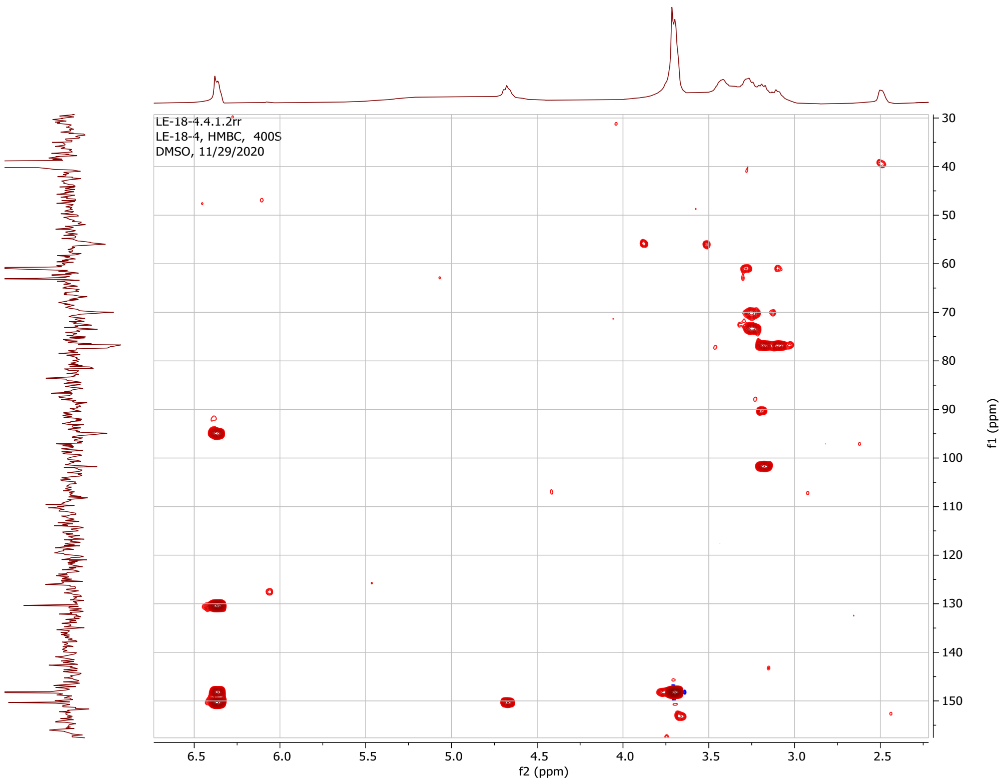

Fig. 19-b HSQC and HMBC spectra of compound 9

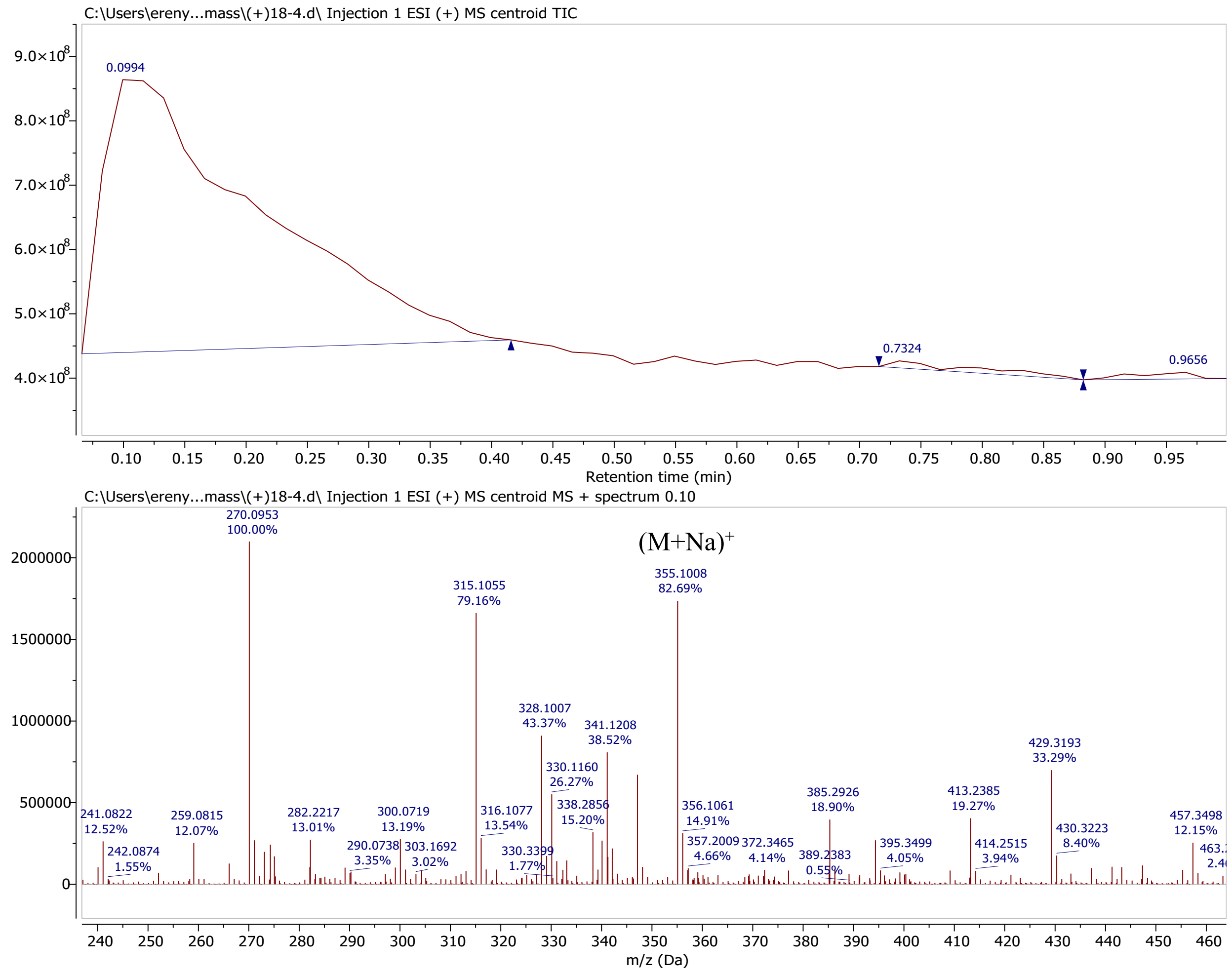

Fig. 19-c HRESIMS spectrum of compound 9 in positive mode

Mixture of Compounds 10 a and 10 b

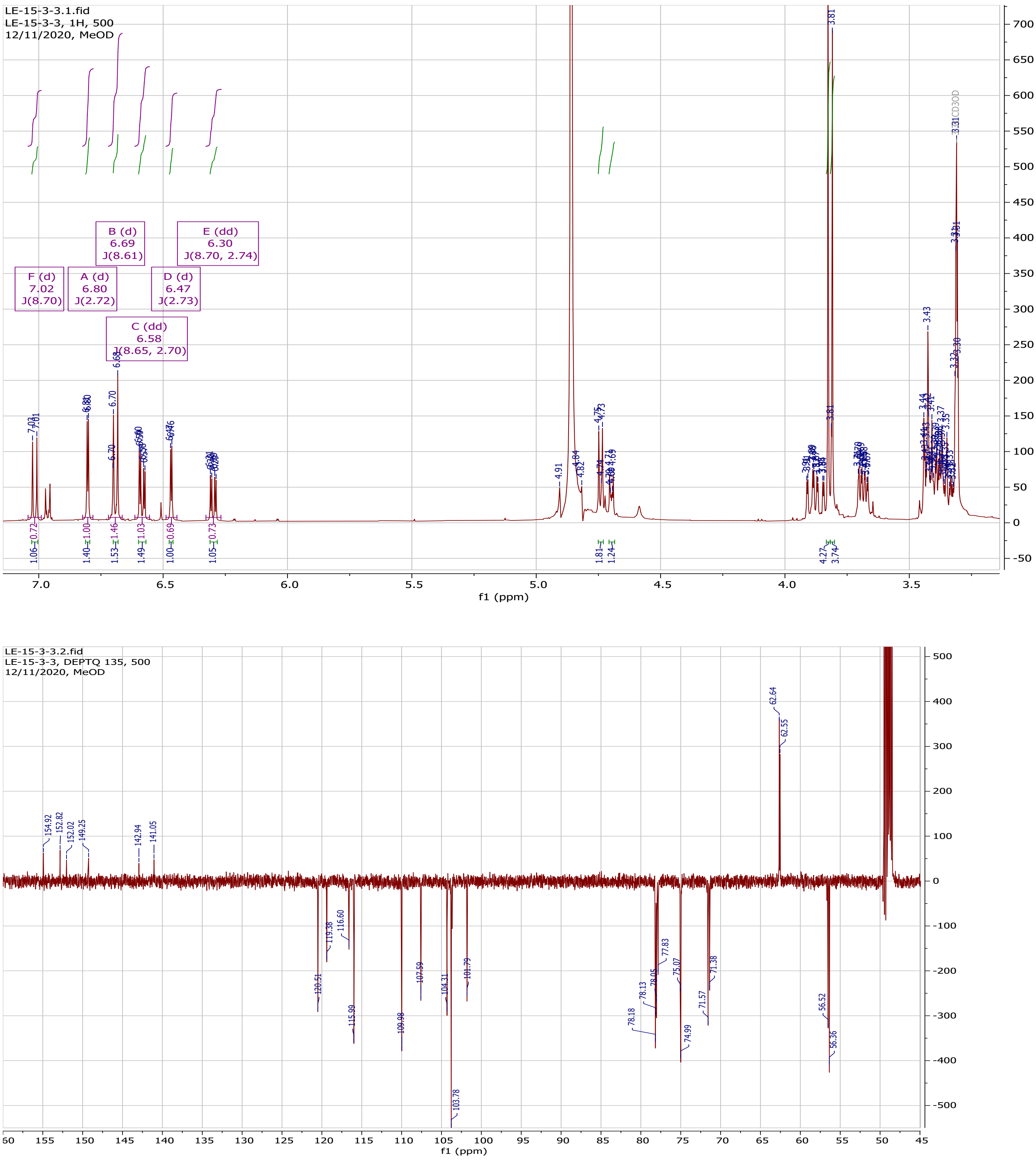

Fig. 20-a ^{1}H NMR, DEPT Q-135 spectra of compounds 10a and 10b

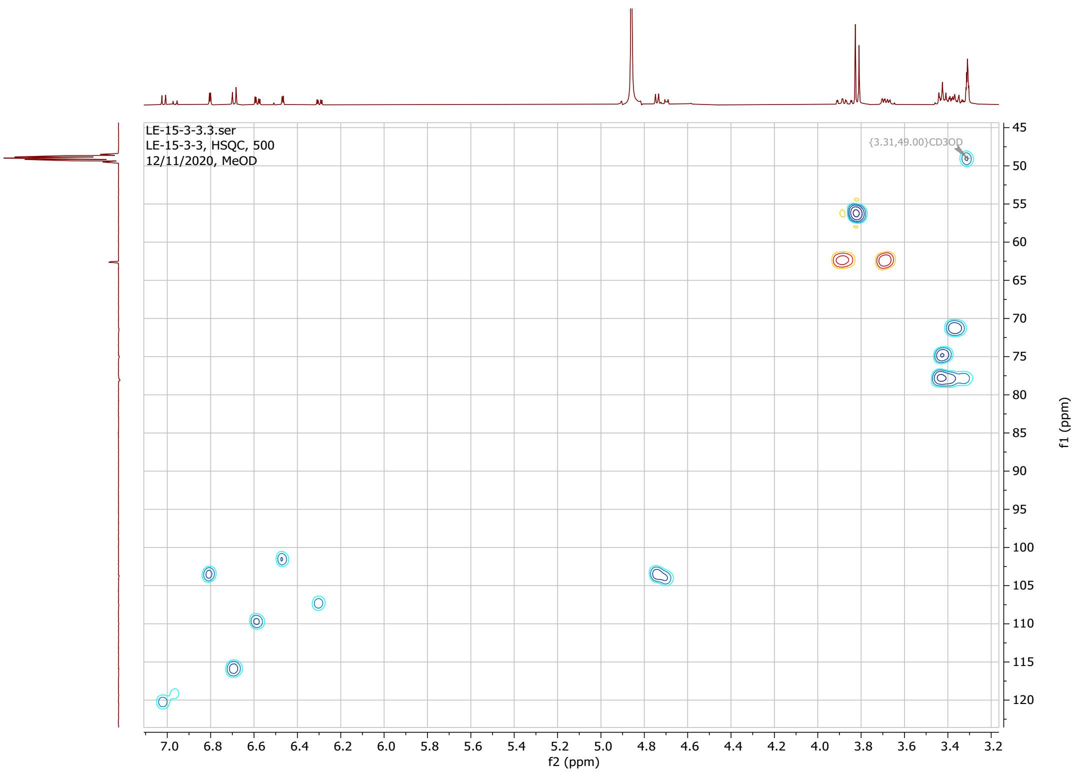

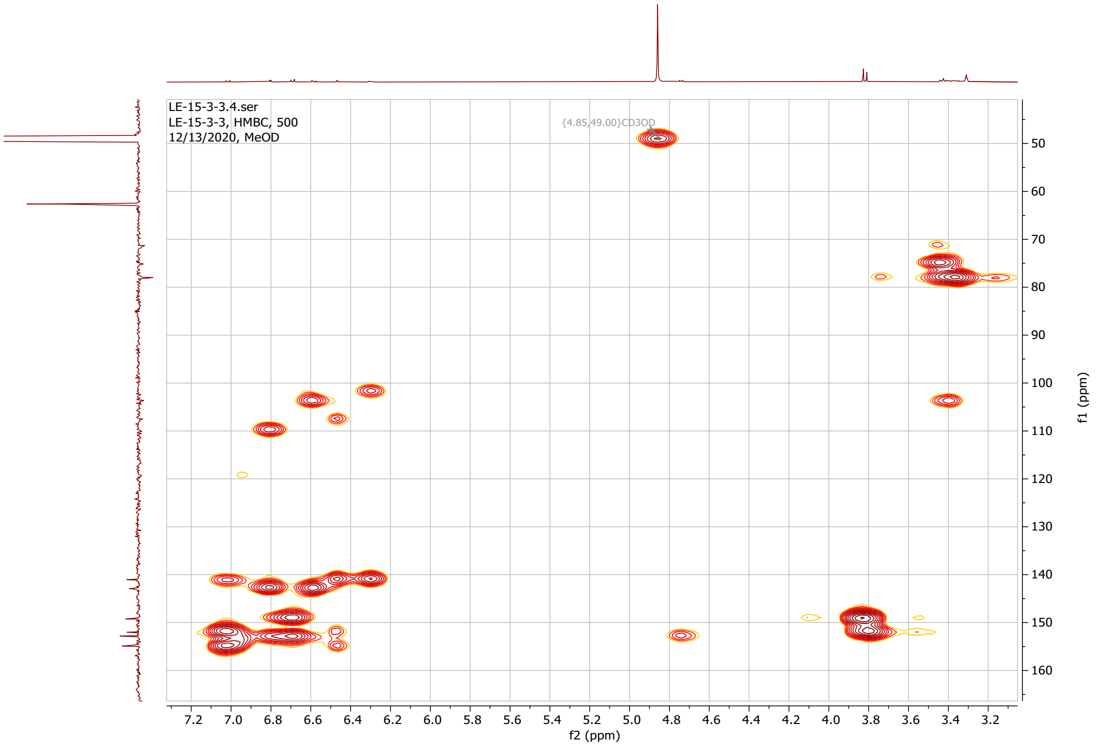

Fig. 20-b HSQC and HMBC spectra of compounds 10a and 10b

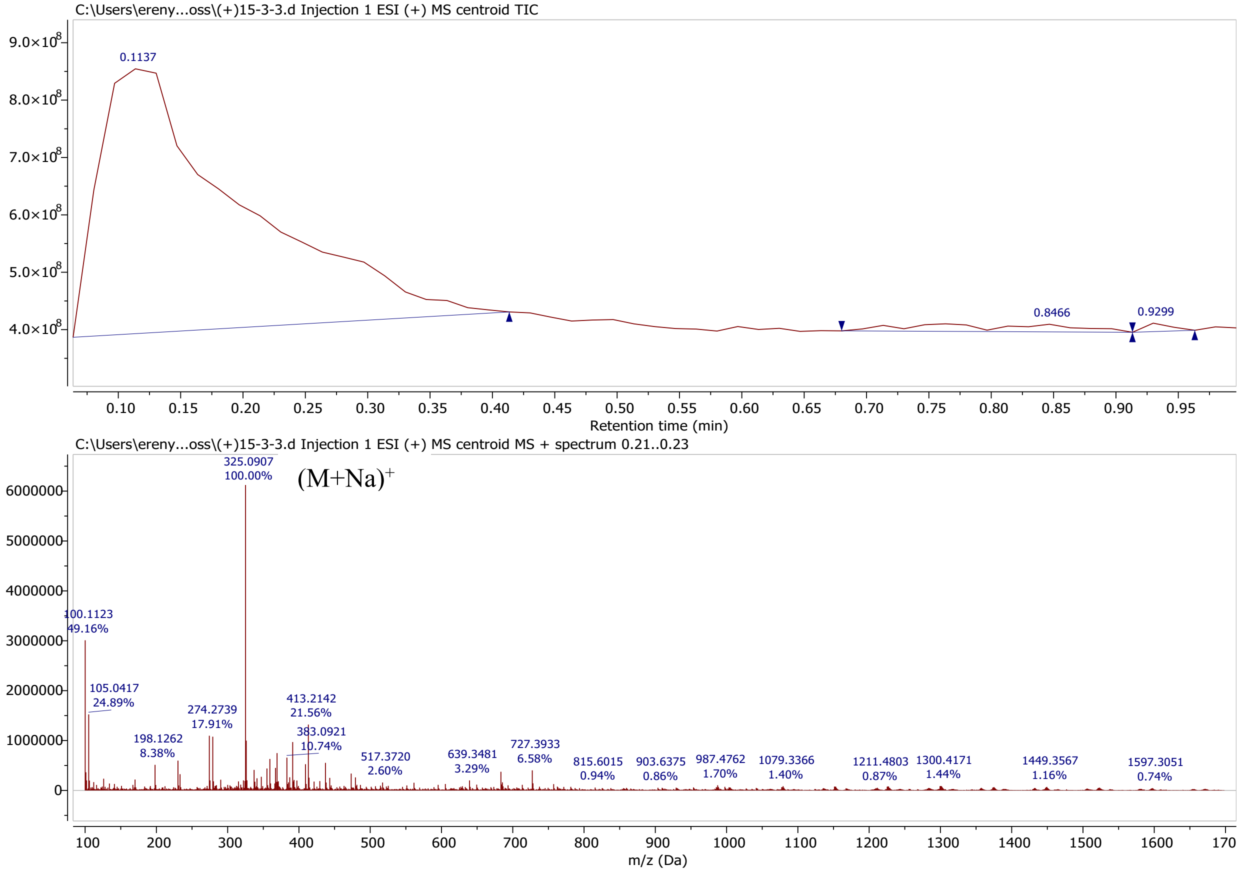

Fig. 20-c HRESIMS spectrum of compounds 10a and 10b in positive mode

Compound 11

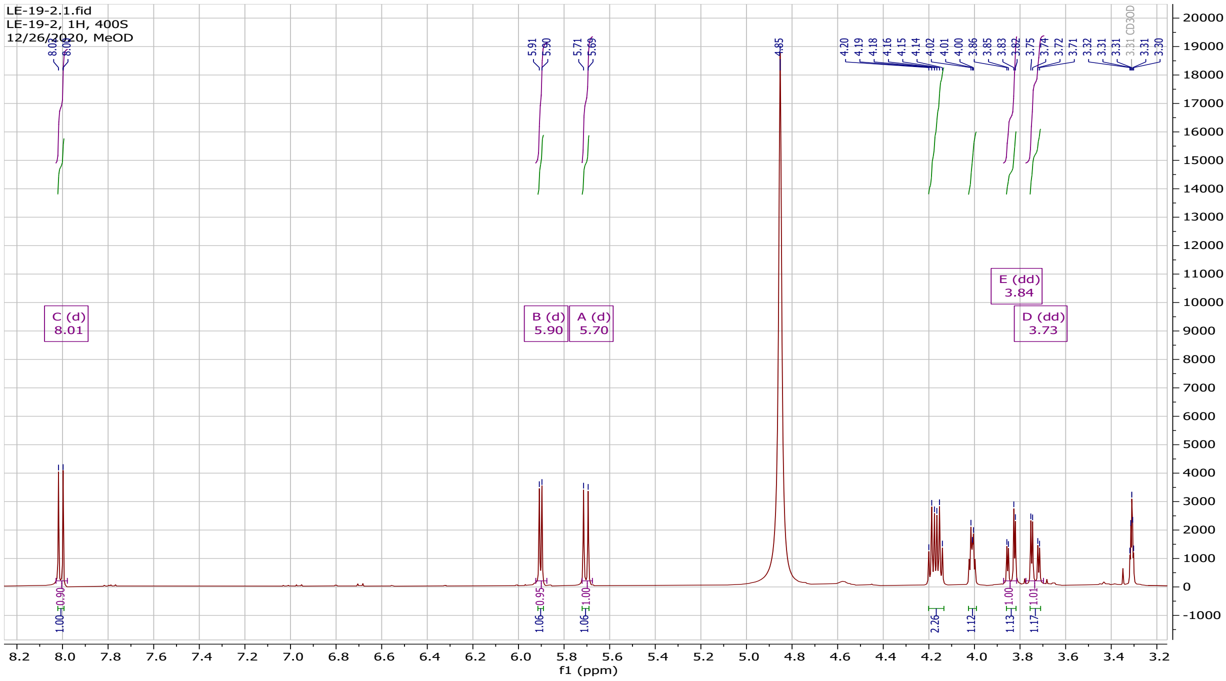

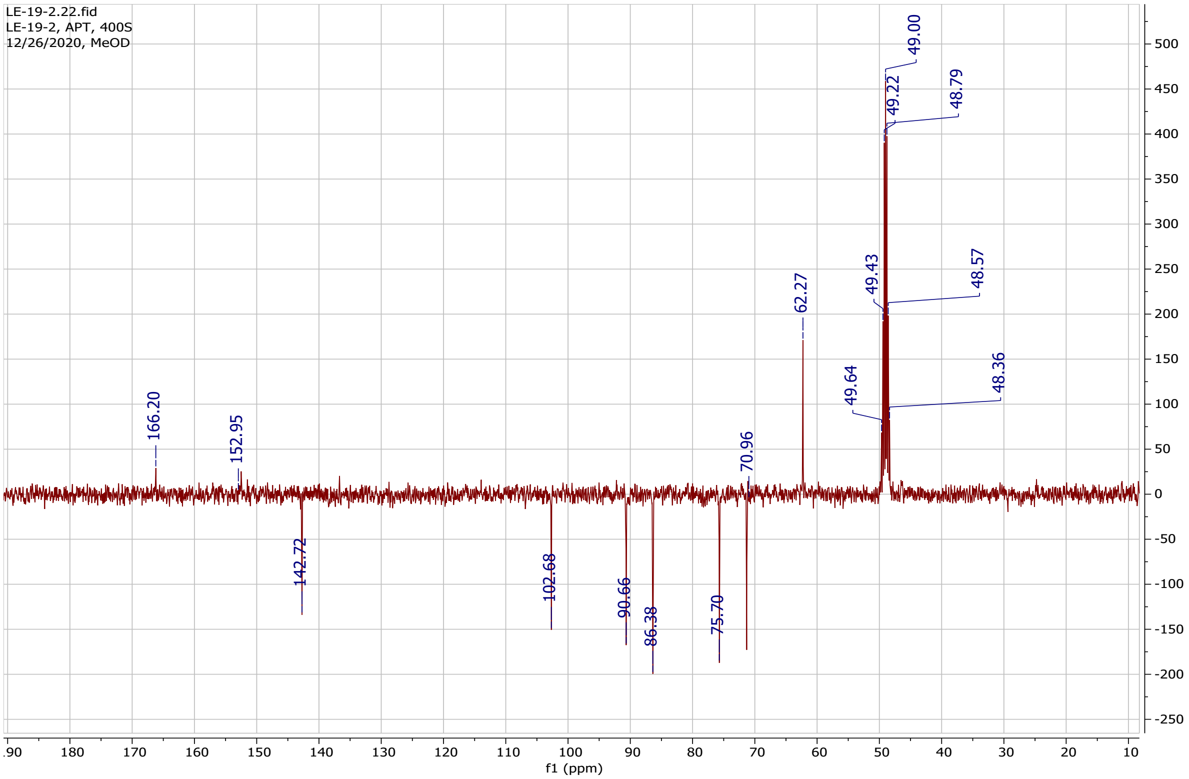

Fig. 21-a ^{1}H NMR and DEPT Q-135 spectra of compound 11

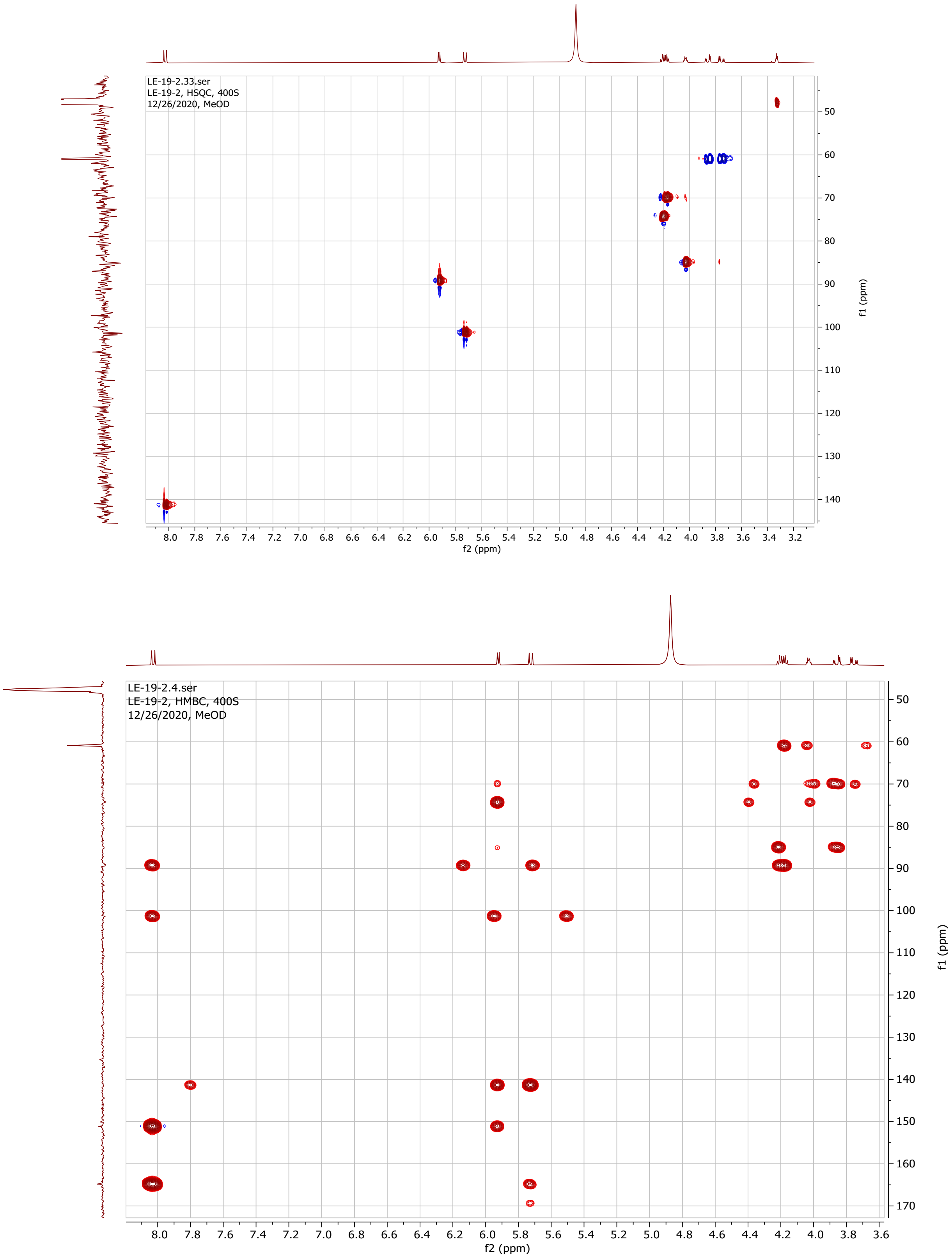

Fig. 21-b HSQC and HMBC spectra of compound 11

Compound 12

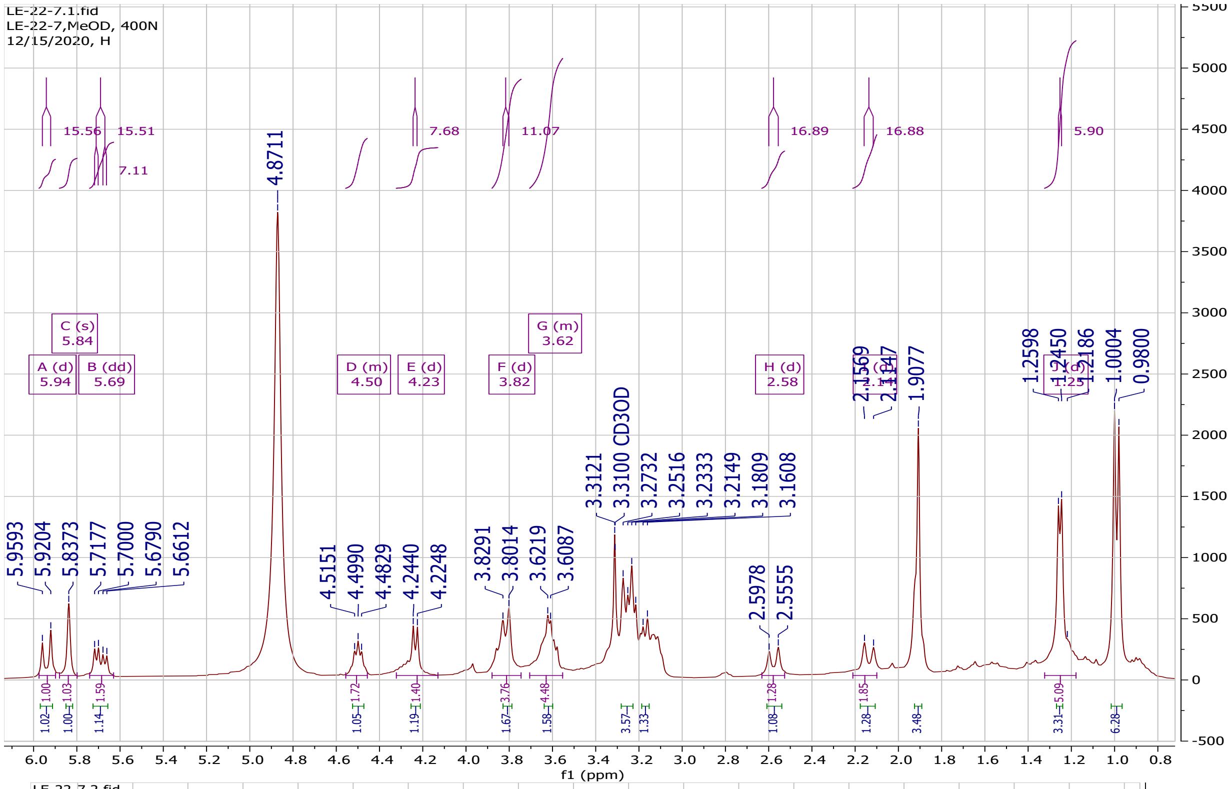

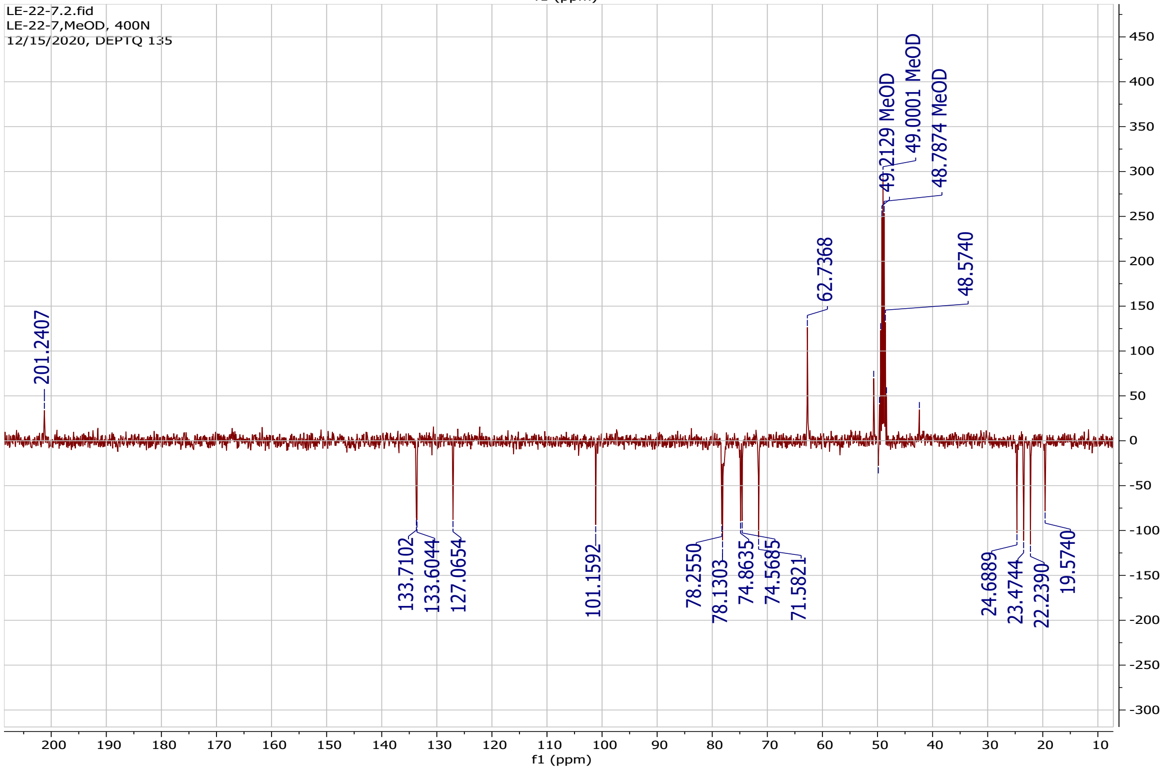

Fig. 22-a ^{1}H NMR and DEPT Q-135 spectra of compound 12

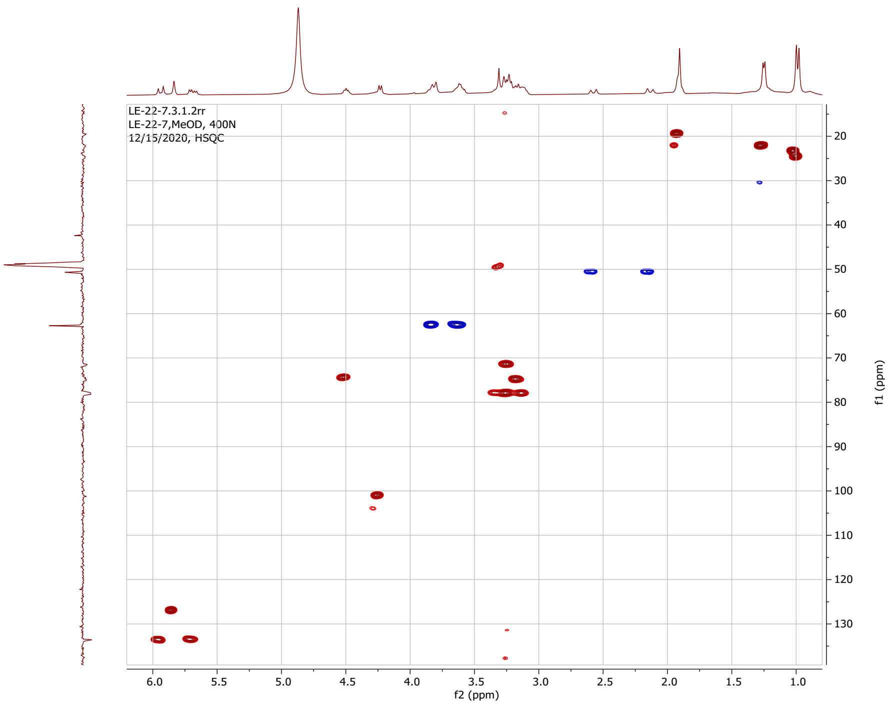

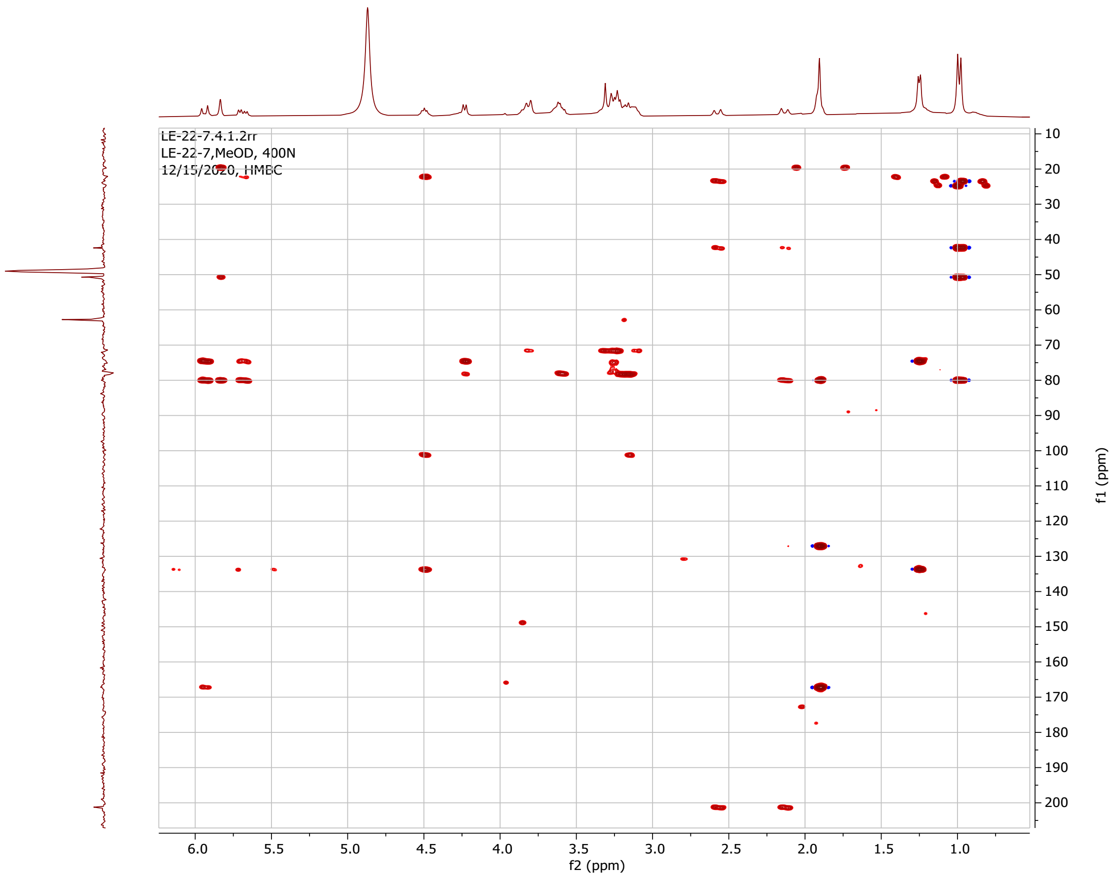

Fig. 22-b HSQC and HMBC spectra of compound 12

II- Molecular docking

To explore the binding patterns, the isolated compounds were docked into LSD1 and CviR protein active sites using a Molecular Operating Environment (MOE; Chemical Computing Group ULC, Montreal, Canada. 2020.09). The crystal structure of tested proteins (Protein Data Bank code: 5YJB) comes from the Research Collaboratory for Structural Bioinformatics Brookhaven Protein Data Bank (https://www.rcsb.org/). Before loading into MOE, the water molecules and nonessential atoms in the protein were removed. Then hydrogenation, protonation and energy minimization of selected proteins were carried out. During the docking process, the compounds were placed using the Triangle Matcher method. To validate the docking protocol, self-docking of the native ligands in the active sites was first performed. Cocrystalized ligands re-docking revealed a docking pose with RMSD of 0.645 and 0.906 Å, respectively. The binding structures were evaluated by Alpha HB Scoring and the final docking conformations were selected according to the docking score and their similarity with the cocrystalized ligands.

1. Docking on LSD1

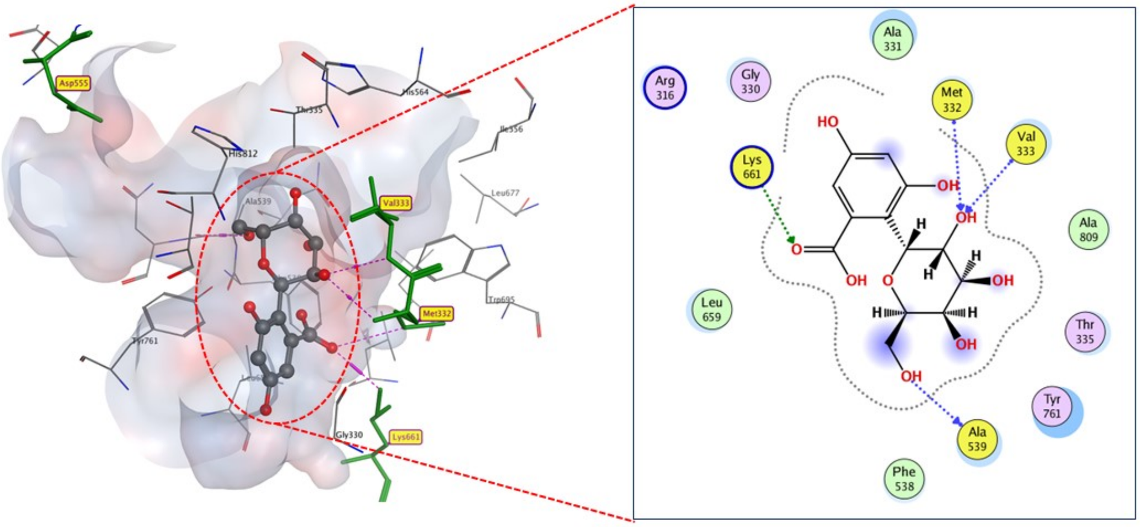

Fig. 23. 2D and 3D display of docking interactions of compound 2 against LSD1.

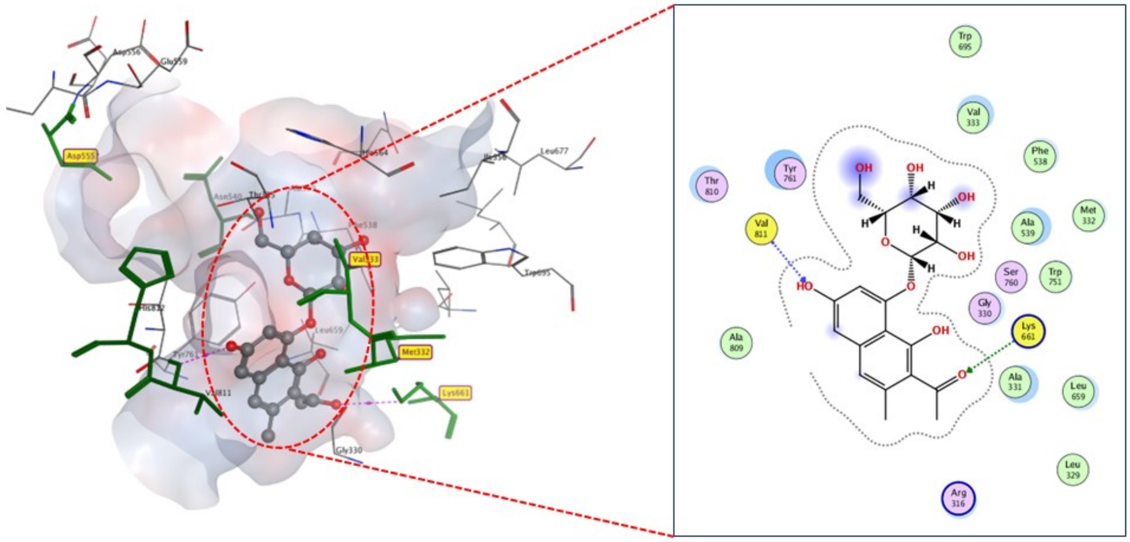

Fig. 24. 2D and 3D display of docking interactions of compound 3 against LSD1.

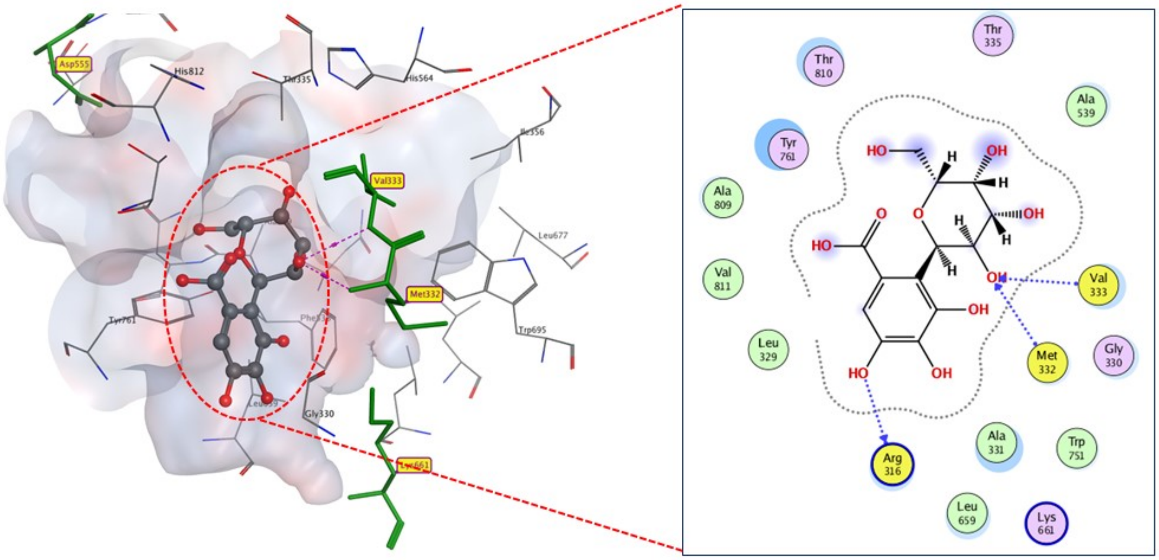

Fig. 25. 2D and 3D display of docking interactions of compound 5 against LSD1.

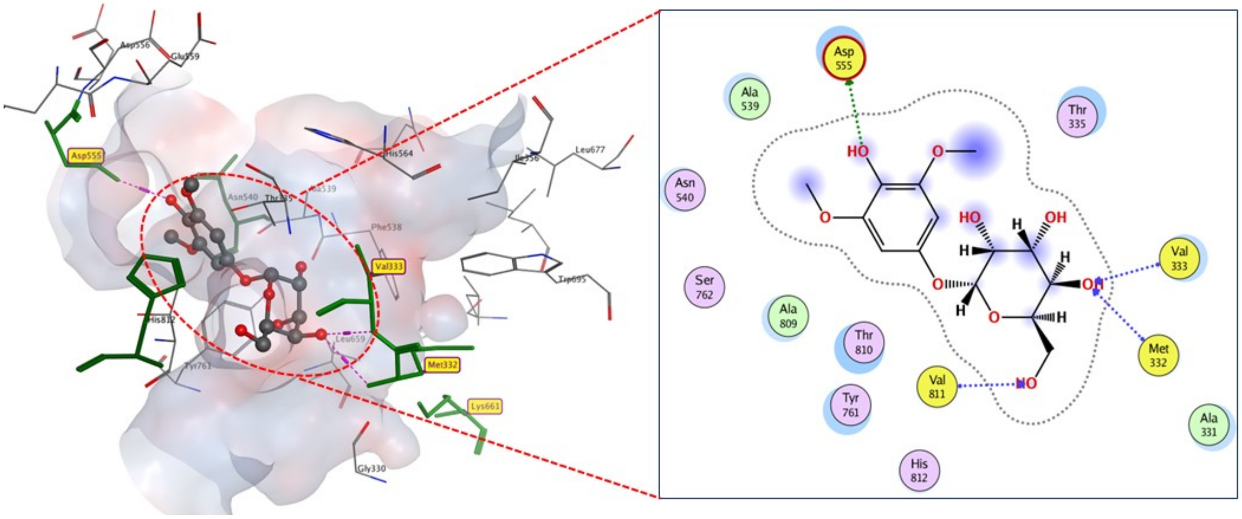

Fig. 26. 2D and 3D display of docking interactions of compound 9 against LSD1.

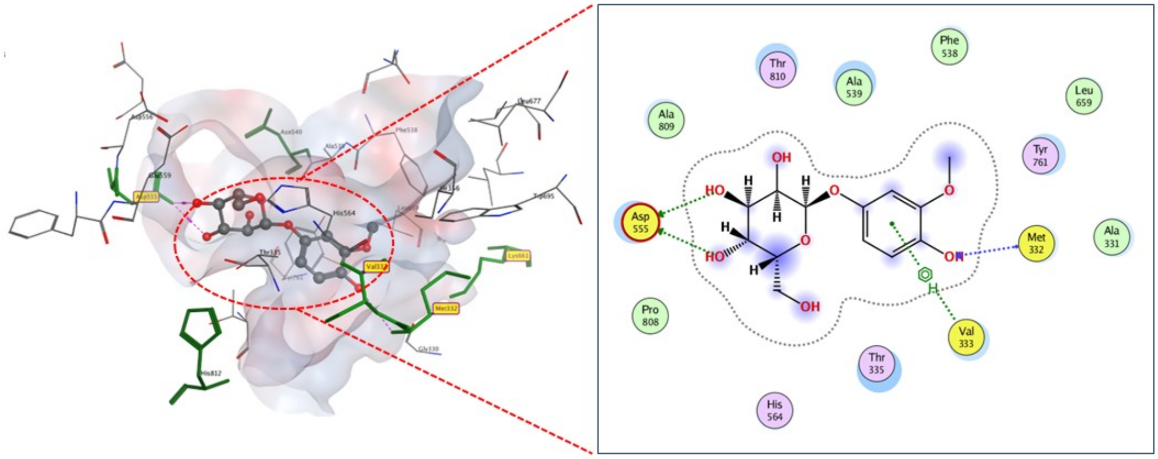

Fig. 27. 2D and 3D display of docking interactions of compound (10-a) against LSD1.

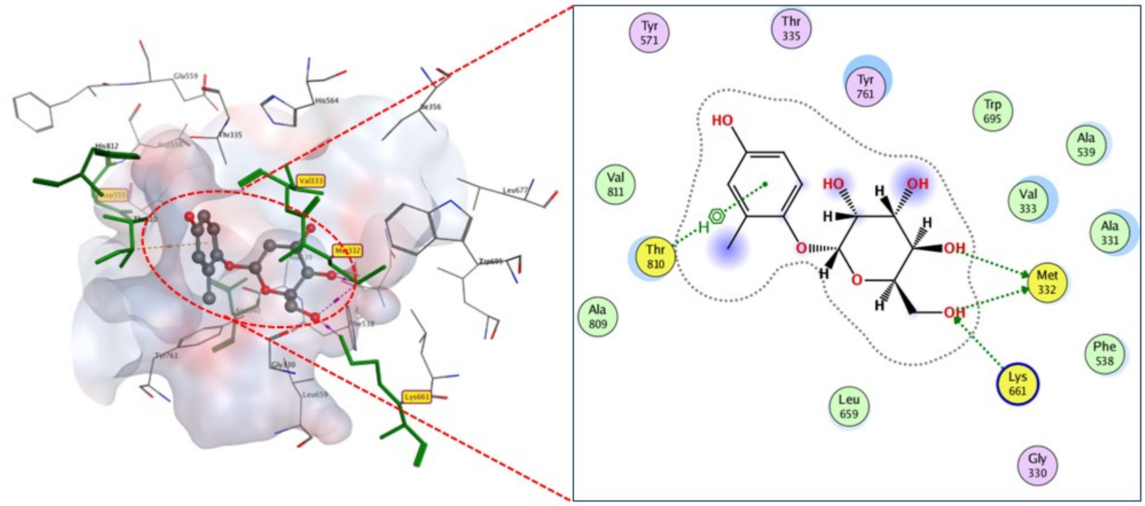

Fig. 28. 2D and 3D display of docking interactions of compound (10-b) against LSD1.

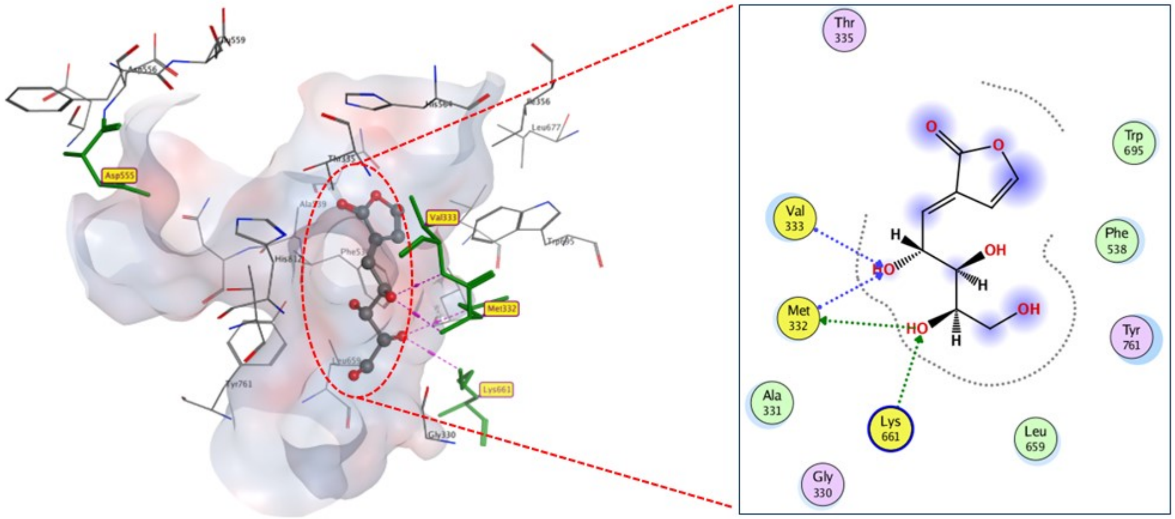

Fig. 29. 2D and 3D display of docking interactions of compound 11 against LSD1.

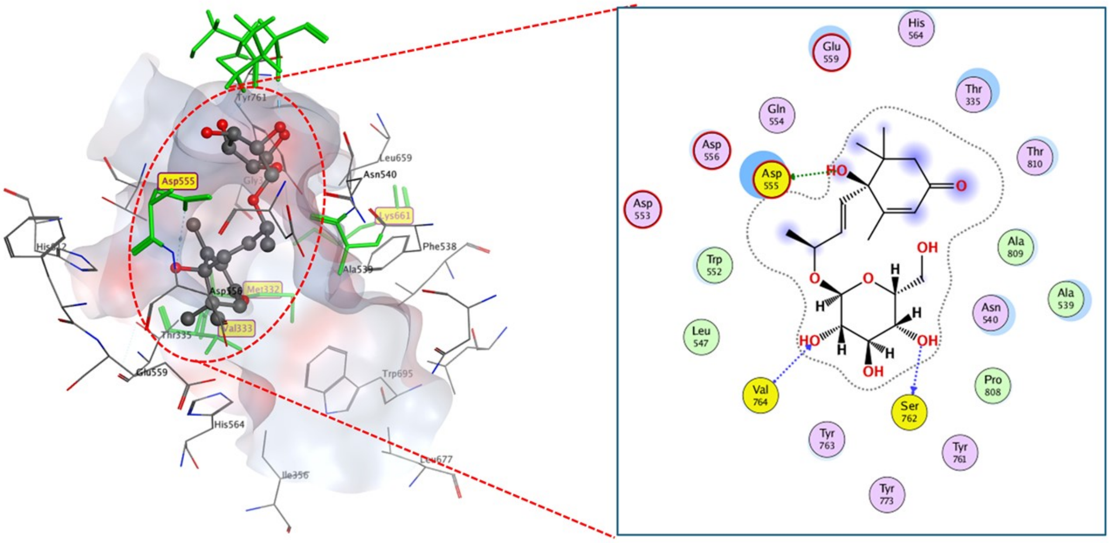

Fig. 30. 2D and 3D display of docking interactions of compound 12 against LSD1.

2. Docking on CviR

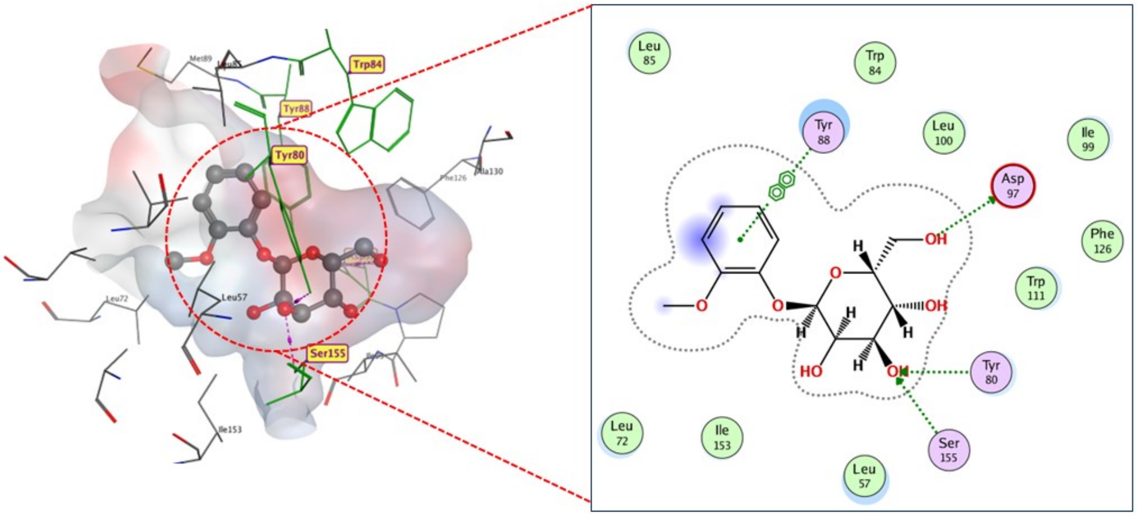

Fig. 31. 2D and 3D display of docking interactions of compound 1 against CviR

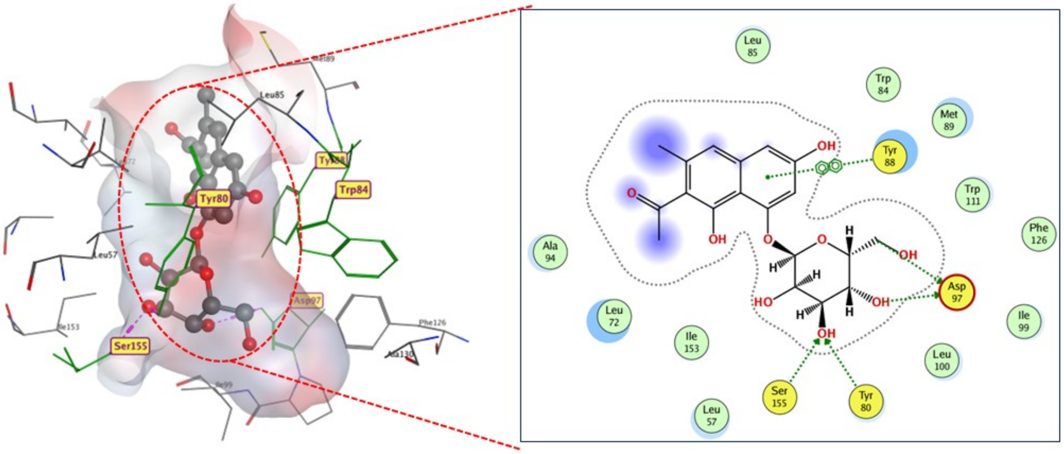

Fig. 32. 2D and 3D display of docking interactions of compound 3 against CviR

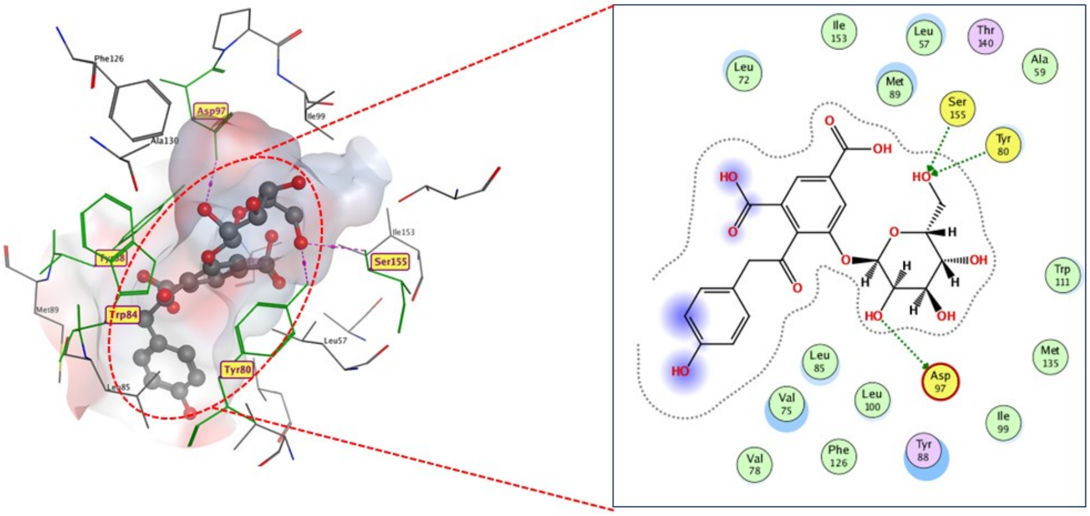

Fig. 33. 2D and 3D display of docking interactions of compound 4 against CviR

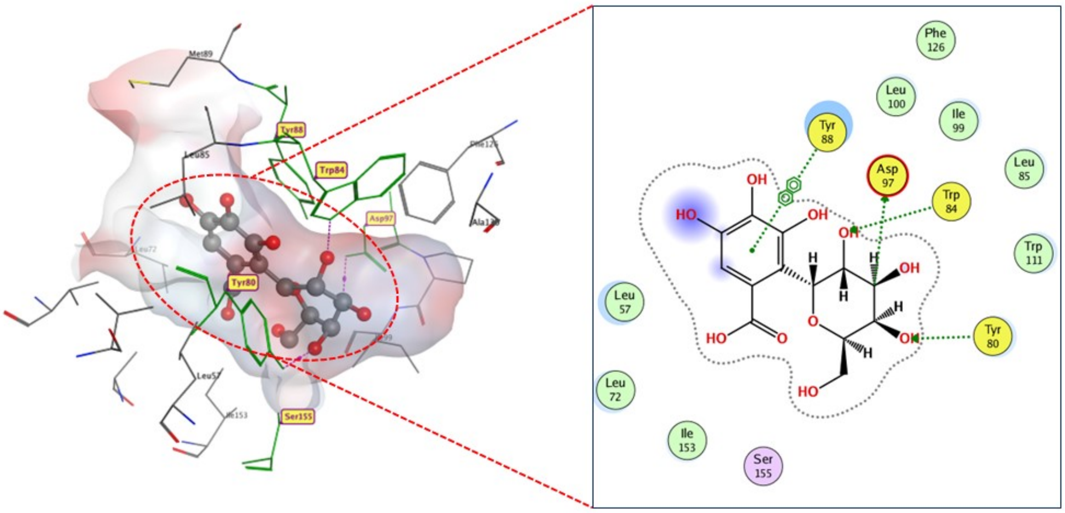

Fig. 34. 2D and 3D display of docking interactions of compound 5 against CviR

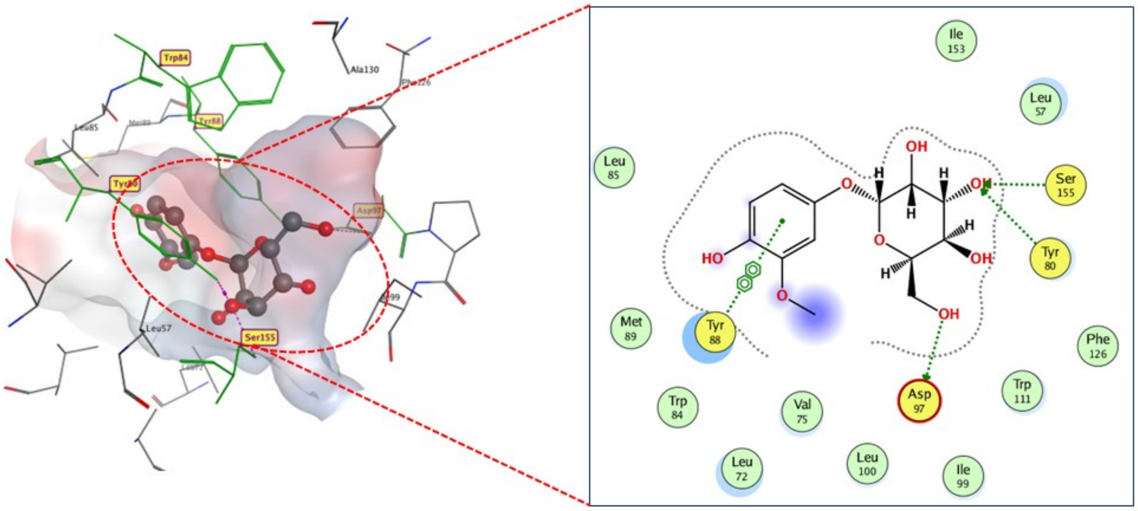

Fig. 35. 2D and 3D display of docking interactions of compound 10-a against CviR

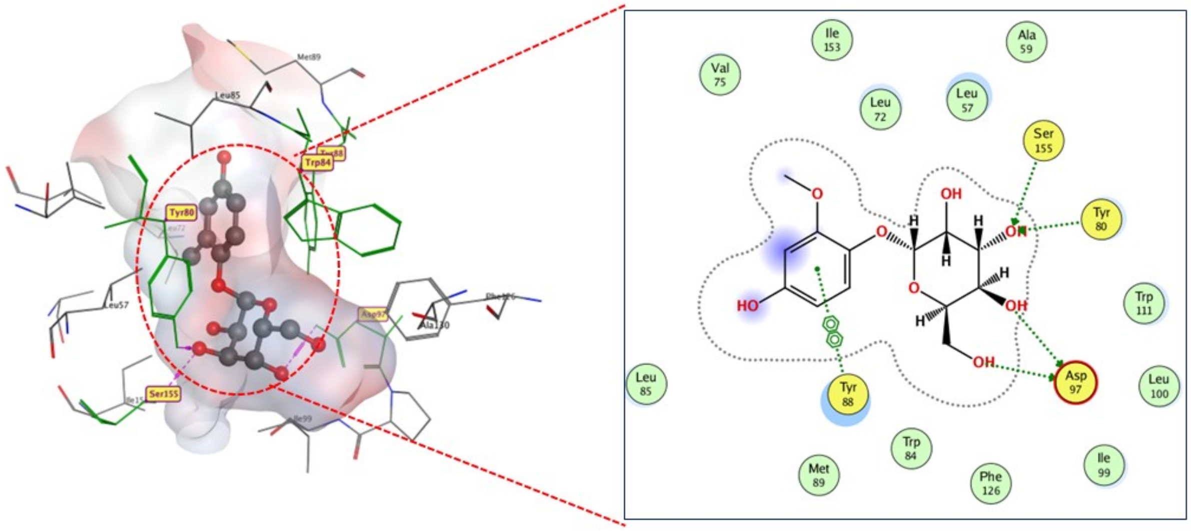

Fig. 36. 2D and 3D display of docking interactions of compound 10-b against CviR

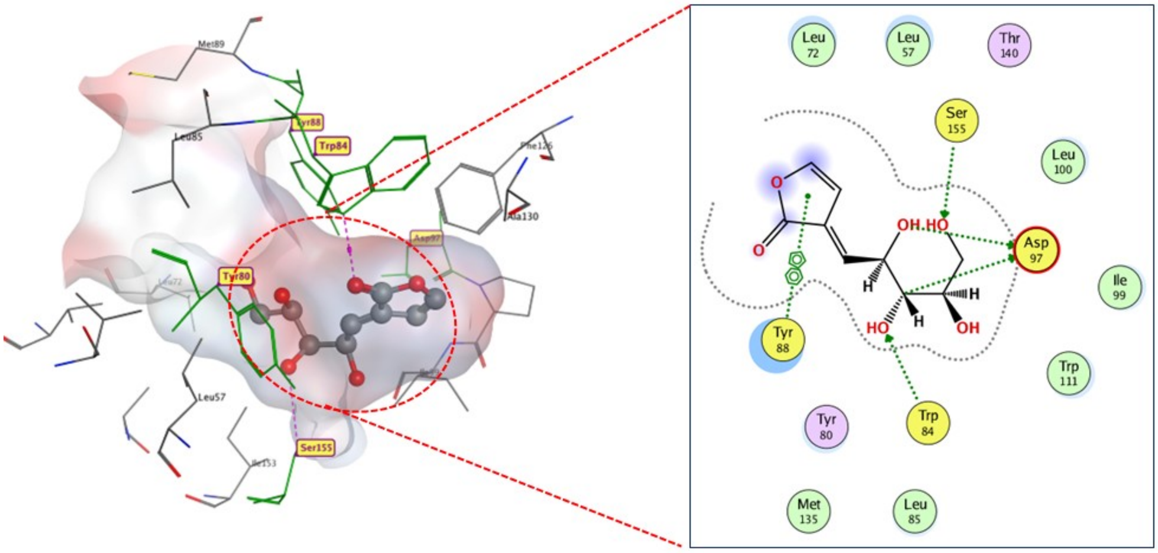

Fig. 37. 2D and 3D display of docking interactions of compound 11 against CviR

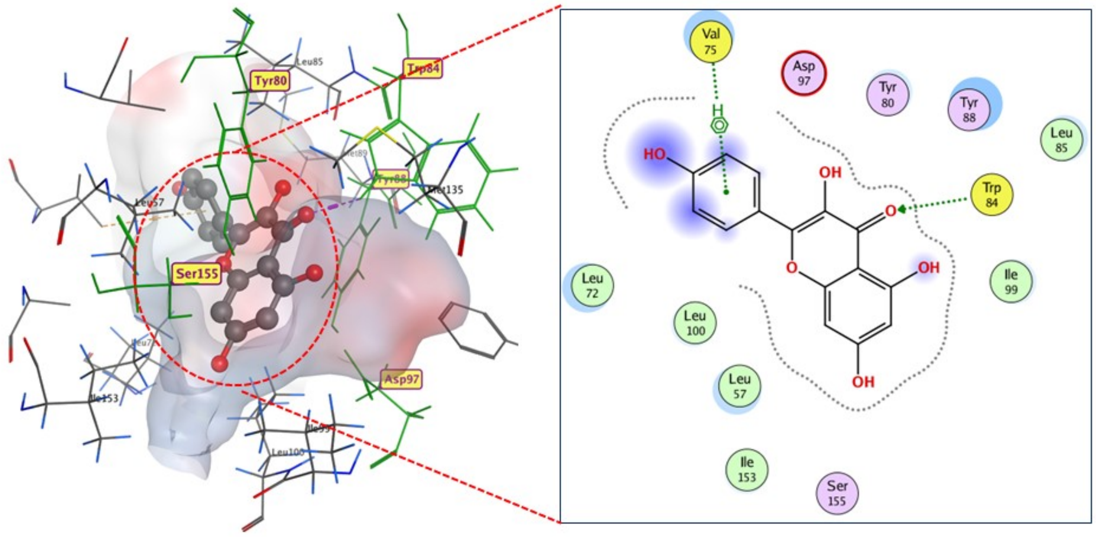

Fig. 38. 2D and 3D display of docking interactions of compound 6 against CviR

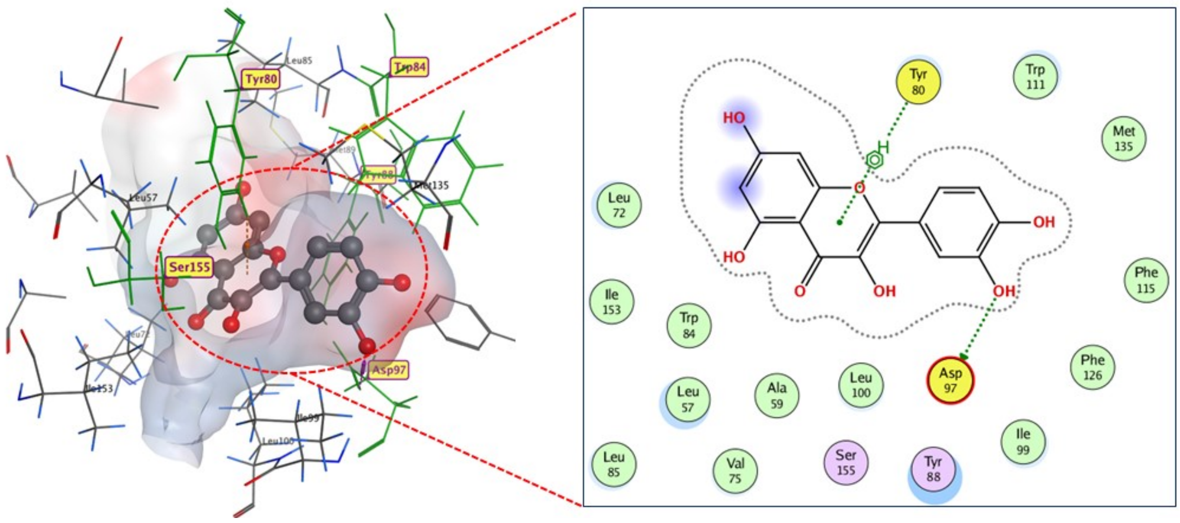

Fig. 39. 2D and 3D display of docking interactions of compound 7 against CviR

III- IR data of compounds 2 and 5

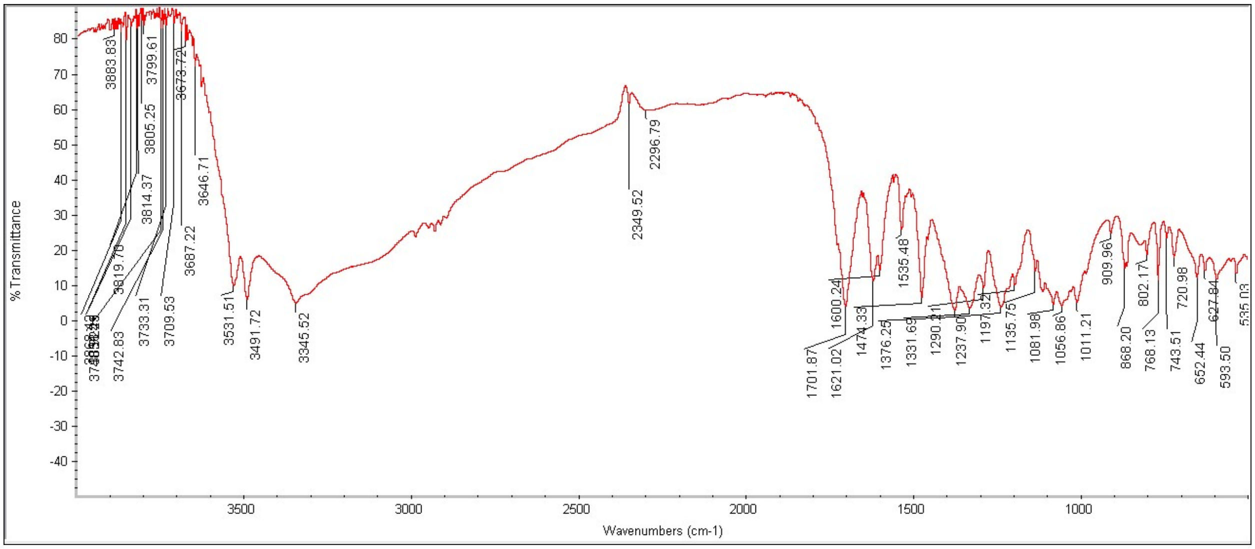

Fig. 40. IR spectrum of compound 2

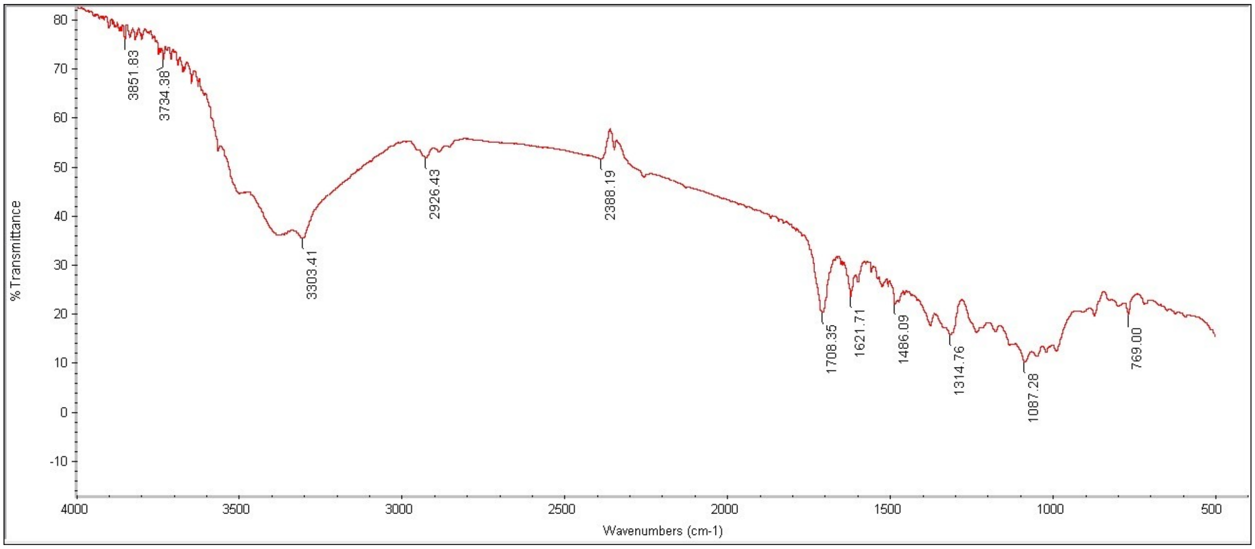

Fig. 41. IR spectrum of compound 5
